# Data-driven classification of individual cells by their non-Markovian motion

**DOI:** 10.1016/j.bpj.2024.03.023

**Published:** 2024-03-21

**Authors:** Anton Klimek, Debasmita Mondal, Stephan Block, Prerna Sharma, Roland R. Netz

**Affiliations:** 1 Fachbereich Physik, Freie Universität Berlin, Berlin, Germany; 2 Institut für Chemie und Biochemie, Freie Universität Berlin, Berlin, Germany; 3 Department of Physics, Indian Institute of Science, Bangalore, India; 4 Department of Bioengineering, Indian Institute of Science, Bangalore, India; 5 James Franck Institute, University of Chicago, Chicago, Illinois

## Abstract

We present a method to differentiate organisms solely by their motion based on the generalized Langevin equation (GLE) and use it to distinguish two different swimming modes of strongly confined unicellular microalgae *Chlamydomonas reinhardtii*. The GLE is a general model for active or passive motion of organisms and particles that can be derived from a time-dependent general many-body Hamiltonian and in particular includes non-Markovian effects (i.e., the trajectory memory of its past). We extract all GLE parameters from individual cell trajectories and perform an unbiased cluster analysis to group them into different classes. For the specific cell population employed in the experiments, the GLE-based assignment into the two different swimming modes works perfectly, as checked by control experiments. The classification and sorting of single cells and organisms is important in different areas; our method, which is based on motion trajectories, offers wide-ranging applications in biology and medicine.

## Significance

Classification of cells is a common task in biology and medicine. We introduce the framework to accomplish such classification based on cell-center trajectories. Our method is based on the systematic theory for the dynamics of coarse-grained variables and extracts the underlying parameters describing active and passive cell motion. We apply our methodology to confined unicellular microalgae that exhibit two different swimming modes and show that we can accurately distinguish the two populations solely based on their motion pattern. Our method can be applied to time-series data of general observables from unicellular and multicellular organisms. We anticipate numerous applications in biology and medicine that require the label-free distinction and analysis of individual cells and organisms.

## Introduction

Classifying individual cells or organisms is a challenging task that has been approached in many different ways and has ample applications. Distinguishing different types of cancer cells (1,2), foodborne pathogens (3), sperm cells (4), or types of neurons (5) are just a few examples. Different techniques have been introduced to distinguish and classify organisms on the multi-cell down to the single-cell level. One approach involves markers that bind cell specifically (2,6,7). Since individual cells contain unique genetic and epigenetic information, it is also possible to distinguish cells by their specific DNA or RNA content. Indeed, biotechnological advances enable single-cell RNA sequencing (8,9), which can be used in combination with machine-learning approaches (10,11), to efficiently distinguish single cells. However, RNA sequencing, as well as usage of markers, requires cell perturbation or even destruction for data acquisition. In many cases, it is desirable to classify cells without perturbing them, which requires label-free techniques such as spectroscopic approaches (3,12) or microscopy (13). In this way, cell information can be extracted almost instantaneously (14) from living organisms (1). Spectroscopic and microscopic images can be processed using machine learning to classify cells; however, the outcomes can be hard to interpret and require massive training data. One way to simplify the processing of cell-image data is to reduce the parameter space. This can be achieved by feature selection (15) or by projection onto important parameters, such as by principal-component analysis (11). A prime feature of mobile cells is their positional trajectory, which is relatively easy to obtain in experiments and contains hidden information on the motion-generating processes within the cell (16,17,18,19,20). Machine-learning algorithms have been proposed to classify trajectories (21) and some have been applied to single-cell trajectories (15). These approaches mostly focus on classification and not on the interpretation of the motion patterns. To yield a mechanistic interpretation, some specific model is usually assumed (22,23,24,25,26,27), which makes the interpretation model dependent. A general model that describes the motion of a particle in a complex environment, captures the stochasticity of its motion and can be derived from first principles is the GLE, which has been shown to accurately describe the motion of different cell types (28,29,30,31,32). In fact, the GLE is not an *ad hoc* model but can be derived from the underlying general many-body Hamiltonian (33,34,35). Living cells are intrinsically out of equilibrium (36), a fact that can be properly accounted for by the GLE used to describe the cell motion (37). In fact, there are many other models besides the GLE that have been successfully used to describe active and passive stochastic motion (38,39,40,41,42). The advantage of the GLE is that it makes minimal assumptions on the type of motion and encompasses many previously introduced models, such as the run-and-tumble model used to describe bacterial motion (43), as has been shown recently (44).

**Figure 1:**
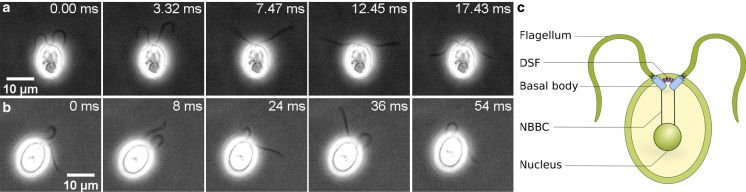
Unicellular microalgae microscopy. Sequences of phase-contrast microscopy images of CR algae exhibiting (*a*) synchro and (*b*) wobbler-type flagellar motion. The white halo around the cells is typical for phase-contrast microscopy (46). (*c*) Sketch of a CR cell: the distal striated fiber (DSF) connects the two basal bodies (47), which anchor the flagella and are connected to the nucleus by nuclear basal-body connectors (NBBCs) (45,48). To see this figure in color, go online.

Here, we present a method to classify individual organisms based on the GLE parameters extracted from their motion trajectories. We apply our methods to experimental trajectories of individual unicellular biflagellate algae *Chlamydomonas reinhardtii* (CR) (45) and find two distinct groups of swimmers, which are illustrated in Fig. 1 *a* and *b*. In contrast to other existing methods for cell sorting and classification, our method requires only trajectories as input, does not need any training of a network, and avoids human bias in the selection of relevant features. Additionally, our approach allows us to interpret motion characteristics in terms of simple mechanistic models derived from the GLE parameters. In the case of CR cells, the data suggest some type of elastic coupling that presumably involves the anchoring of the flagella (45,47,48), as schematically depicted in Fig. 1 *c*, or a chemical feedback loop. Our approach is applicable to any kind of cell or organism motility data with sufficiently long trajectories and sufficiently fine temporal discretization if the coordinate describing the motion corresponds to a Gaussian process, as will be explained in detail further below.

## Materials and methods

### Cell growth and sample preparation

Wild-type CR cell cultures (strain, CC-1690) are grown in tris-acetate-phosphate (TAP + P) medium by alternating light:dark (12:12 h) cycles for 3 days. We collect the cell suspension in their actively growing phase (between third and sixth day of culture) 2–3 h after the beginning of the light cycle and re-suspend it in fresh TAP + P medium. After 30 to 40 min of equilibration to recover from the mechanosensitive shock during re-suspension (49), we inject the cells inside a rectangular quasi-2D microfluidic chamber of height 10μm and area 18×6mm. This chamber is assembled by using a glass slide and coverslip sandwiched with a 10−μm double-sided tape (Nitto Denko corporation) as spacer. The glass surfaces are pre-cleaned and coated with a polyacrylamide brush to suppress nonspecific adhesion of cell body and flagella (50). The chamber height is determined as 10.88±0.68μm across different samples (51).

### Recording of trajectories

The cells in the chamber are placed under red light illumination (>610nm) to prevent phototaxis (52) and flagellar adhesion (53) of CR (51). We use high-speed video microscopy (Olympus IX83/IX73) at 500 frames per second with a 40× phase-contrast objective (Olympus, 0.65 NA, Plan N, PH2) connected to a metal oxide semiconductor (CMOS) camera (Phantom Miro C110, Vision Research, pixel size = 5.6μm) for imaging the mid-plane between the confining glass plates. This setup enables us to simultaneously image cell position and flagellar shape. To capture very long trajectories to probe the long-time diffusive behavior in the supporting material, we use a 10× bright-field objective (Olympus, 0.25 NA, PlanC N) connected to a high-speed CMOS camera of higher pixel length (pco.1200hs, pixel size = 12μm) at 50 frames per second. We determine cell trajectories by binarizing the image sequences with appropriate threshold parameters and tracking their centers using standard MATLAB routines (54).

### Velocity autocorrelation function

Fourier transformation of Eqs. 23 and 24 leads to

(Equation 1) $$\tilde{v}\left(\omega \right)=\frac{{\tilde{F}}_{R}\left(\omega \right)}{{\tilde{\Gamma }}_{v}^{+}\left(\omega \right)+i\omega }$$

with the single-sided Fourier transform defined as ${\tilde{\Gamma }}_{v}^{+}\left(\omega \right)=\int_{0}^{\infty }e^{-i\omega t}\Gamma_{v}\left(t\right)dt$ and

(Equation 2) $$\left\langle {\tilde{F}}_{R}\left(\omega \right){\tilde{F}}_{R}\left(\omega^{\prime }\right)\right\rangle =2\pi B\delta \left(\omega +\omega^{\prime }\right){\tilde{\Gamma }}_{R}\left(\omega^{\prime }\right)\;\text{.}$$

From the Fourier transform of the velocity autocorrelation function (VACF)

(Equation 3) $$\begin{array}{ll} {\tilde{C}}_{vv}\left(\omega \right) & =\int_{-\infty }^{\infty }dte^{-i\omega t}\left\langle v\left(0\right)v\left(t\right)\right\rangle \\ & =\int_{-\infty }^{\infty }e^{-i\omega t}dt\int_{\infty }^{\infty }e^{i\omega t}\frac{d\omega }{2\pi }\int_{-\infty }^{\infty }\frac{d\omega }{2\pi }\left\langle \tilde{v}\left(\omega \right)\tilde{v}\left(\omega^{\prime }\right)\right\rangle \end{array}$$

we obtain by inserting Eqs. 1 and 2

(Equation 4) $${\tilde{C}}_{vv}\left(\omega \right)=\frac{B{\tilde{\Gamma }}_{R}\left(\omega \right)}{\left({\tilde{\Gamma }}_{v}^{+}\left(\omega \right)+i\omega \right)\left({\tilde{\Gamma }}_{v}^{+}\left(-\omega \right)-i\omega \right)}\;\text{.}$$

Equating the nonequilibrium and the surrogate VACF in Eqs. 26 and 27 leads to

(Equation 5) $$\frac{{\tilde{\Gamma }}_{R}\left(\omega \right)}{\left({\tilde{\Gamma }}_{v}^{+}\left(\omega \right)+i\omega \right)\left({\tilde{\Gamma }}_{v}^{+}\left(-\omega \right)-i\omega \right)}=\frac{\tilde{\Gamma }\left(\omega \right)}{|{\tilde{\Gamma }}^{+}\left(\omega \right)+i\omega {|}^{2}}\;\text{.}$$

In the supporting material, we show that, for every correlation function $C_{vv}\left(t\right)$, we can determine a unique Γ(t).

### Memory kernel extraction

Multiplying the GLE Eq. 23 by $\dot{x}\left(t_{0}\right)$, averaging over the random force and integrating from $t_{0}$ to *t* leads to

(Equation 6) $$\left(C_{vv}\left(t\right)-C_{vv}\left(0\right)\right)=-\int_{0}^{t}C_{vv}\left(s\right)G\left(t-s\right)ds\text{,}$$

where we used that $\left\langle \dot{x}\left(t_{0}\right)F_{R}\left(t\right)\right\rangle =0$ (33,34,35), set $t_{0}=0$, and introduced the integral kernel

(Equation 7) $$G\left(t\right)=\int_{0}^{t}\Gamma \left(s\right)ds\;\text{.}$$

To invert Eq. 6, we discretize it. Since $C_{vv}\left(t\right)$ is even but G(t) is odd, we discretize G(t) on half steps and $C_{vv}\left(t\right)$ on full steps and obtain (30)

(Equation 8) $$G_{i+1/2}=\frac{2\left(C_{vv}^{0}-C_{vv}^{i+1}\right)}{\Delta \left(C_{vv}^{1}+C_{vv}^{0}\right)}-\sum_{j=1}^{i}G_{i-j+1/2}\frac{C_{vv}^{j+1}+C_{vv}^{j}}{C_{vv}^{1}+C_{vv}^{0}}\text{.}$$

The kernel $\Gamma_{i}$ is obtained by the discrete derivative $\Gamma_{i}=\frac{G_{i+1/2}+G_{i-1/2}}{\Delta }$ with the initial value $\Gamma_{0}=2G_{1/2}/\Delta$.

### Two-point velocity distribution

A stationary Gaussian process is completely described by its two-point probability distribution as shown in the supporting material. Here we show that the joint and conditional velocity distributions only depend on the VACF. The joint probability to observe $v_{2}$ at time $t_{2}$ and $v_{1}$ at time $t_{1}$ can be written in terms of the velocity vector $\vec{v}={\left(v_{1},v_{2}\right)}^{T}$ as

(Equation 9) $$p\left(v_{2},t_{2};v_{1},t_{1}\right)=\frac{\;\exp\;\left(-{\vec{v}}^{T}\Sigma^{-1}\left(t_{2}-t_{1}\right)\vec{v}/2\right)}{\sqrt{2\pi \left|\Sigma \left(t_{2}-t_{1}\right)\right|}}$$

with $|\Sigma \left(t_{2}-t_{1}\right)|$ denoting the determinant of the covariance matrix

(Equation 10) $$\begin{array}{cc} \Sigma & =\left(\begin{array}{cc} \left\langle v\left(t_{1}\right)v\left(t_{1}\right)\right\rangle & \left\langle v\left(t_{1}\right)v\left(t_{2}\right)\right\rangle \\ \left\langle v\left(t_{1}\right)v\left(t_{2}\right)\right\rangle & \left\langle v\left(t_{2}\right)v\left(t_{2}\right)\right\rangle \end{array}\right)\\ & =\left(\begin{array}{cc} C_{vv}\left(0\right) & C_{vv}\left(t_{2}-t_{1}\right)\\ C_{vv}\left(t_{2}-t_{1}\right) & C_{vv}\left(0\right) \end{array}\right)\text{.} \end{array}$$

Using the normal velocity distribution

(Equation 11) $$p\left(v\right)=\frac{\;\exp\;\left(\frac{-v^{2}}{2C_{vv}\left(0\right)}\right)}{\sqrt{2\pi C_{vv}\left(0\right)}}\text{,}$$

we obtain the conditional probability that $v\left(t_{2}\right)=v_{2}$ given $v\left(t_{1}\right)=v_{1}$, as

(Equation 12) $$\begin{array}{cc} p\left(v_{2},t_{2}|v_{1},t_{1}\right) & =\frac{p\left(v_{2},t_{2};v_{1},t_{1}\right)}{p\left(v_{1},t_{1}\right)}\\ & =\frac{\;\exp\;\left(-\frac{{\left[v_{2}-v_{1}\left(C_{vv}\left(t_{2}-t_{1}\right)\right)/C_{vv}\left(0\right)\right]}^{2}}{\left(C_{vv}\left(0\right)-C_{vv}^{2}\left(t_{2}-t_{1}\right)/C_{vv}\left(0\right)\right)}\right)}{\sqrt{2\pi \left(C_{vv}\left(0\right)-C_{vv}^{2}\left(t_{2}-t_{1}\right)/C_{vv}\left(0\right)\right)}}\;\text{.} \end{array}$$

Thus, the joint and conditional distributions only depend on the VACF $C_{vv}\left(t\right)$.

### Fitting of friction kernel

The extracted friction kernel of each individual cell is fitted to Eq. 29 by least-square minimization (using the curve fit function of python’s *scipy*) of the first 0.2s of the data, which allows to estimate the parameter standard deviation by the diagonal of the parameter covariance. The *δ*-peak in Eq. 29 leads to the initial kernel value $\Gamma_{0}=2a/\Delta +b$ where Δ denotes the discretization time (see section “memory kernel extraction”). Since single cells exhibit large variations of the friction kernels, we constrain *a* and *b* in Eq. 29 to be between 0.1% and 99.9% of $\Gamma_{0}$. The decay time *τ* is constrained to be between 0.05 and 3s and the frequency Ω is constrained between 20 and $250\;s^{-1}$.

### Discretized VACF including localization noise

To test whether the cell motion is actually described by the friction kernel (Eq. 29) and does not originate from the experimental finite time step or noise, we fit the experimental VACF of individual cells with an analytical model that accounts for finite time discretization and noise (30). Here, we explain the fitting procedure; the analytical expression for the mean-squared displacement (MSD) using a friction kernel in the form of Eq. 29 is derived in the supporting material.

We denote discrete values of a function f(t) as $f\left(i\Delta \right)=f_{i}=f^{i}$ and the discretization time step as Δ. After smoothing the data by averaging over consecutive positions to reduce the localization noise, as discussed in detail in the supporting material, the velocities at half time steps follow as

(Equation 13) $$v_{i+\frac{1}{2}}=\frac{x_{i+1}-x_{i}}{\Delta }\;\text{.}$$

From the velocities, the VACF defined by Eq. 25 is calculated according to

(Equation 14) $$C_{vv}^{i}=\frac{1}{N+1-i}\sum_{j=0}^{N-i}v_{j+\frac{1}{2}}v_{j+i+\frac{1}{2}}\text{,}$$

with *N* being the number of trajectory steps. To account for localization noise, we assume Gaussian uncorrelated noise of width $\sigma_{loc}$ at every time step, which gives the noisy MSD as (30)

(Equation 15) $$C_{\text{MSD}}^{\text{noise}}\left(t\right)=C_{\text{MSD}}^{\text{theo}}\left(t\right)+2\left(1-\delta_{t0}\right)\sigma_{\text{loc}}^{2}\text{,}$$

where $C_{\text{MSD}}^{\text{theo}}\left(t\right)$ is the theoretical expression for the model MSD given in the supporting material, Eq. S51, and $\delta_{t0}$ is the Kronecker delta reflecting the uncorrelated nature of the localization noise. Since the observed trajectories are sampled with a finite time step Δ, we discretize the relation

(Equation 16) $$C_{vv}\left(t\right)=\frac{1}{2}\frac{d^{2}}{dt^{2}}C_{\text{MSD}}\left(t\right)\text{,}$$

which leads to

(Equation 17) $$C_{vv}^{\text{fit}}\left(i\Delta \right)=\frac{C_{\text{MSD}}^{\text{noise}}\left(\left(i+1\right)\Delta \right)-2C_{\text{MSD}}^{\text{noise}}\left(i\Delta \right)+C_{\text{MSD}}^{\text{noise}}\left(\left(i-1\right)\Delta \right)}{2\Delta^{2}}\text{.}$$

Finally, fits are performed by minimizing the cost function

(Equation 18) $$E_{\text{cost}}=\sum_{i=0}^{n}{\left(C_{vv}^{\exp}\left(i\Delta \right)-C_{vv}^{\text{fit}}\left(i\Delta \right)\right)}^{2}$$

with SciPy’s least squares function in python and using Eq. 17 to determine $C_{vv}^{\text{fit}}\left(t\right)$ at discrete time points. As the MSD and VACF follow from the GLE Eq. 23 and the friction kernel (Eq. 29), the parameters to optimize are the kernel parameters *a*, *b*, *τ*, Ω, the mean-squared velocity *B*, and the localization noise width $\sigma_{\text{loc}}$. The data is fitted up to 0.2s to disregard the noisy part of the VACF (see the supporting material for details). The cluster analysis is performed on the reduced parameter sets *a*, *b*, *τ*, Ω, *B*, which are obtained from the direct fit of Eq. 29 to the extracted kernel from the data.

### Cluster analysis

The friction kernel in Eq. 29 contains four parameters; together with the mean-squared velocity *B*, each individual cell is characterized by five parameters. We perform an X-means cluster analysis (55), which is a generalization of the k-means algorithm (56). The k-means algorithm assigns unlabeled data to a predetermined number of *k* clusters by minimizing distances to the cluster centers. In the X-means algorithm, the number of clusters is not predetermined; we allow cluster numbers from 2 to 20. The algorithm starts with the minimal number of clusters and finds the cluster centers using k-means. It then splits every cluster into two subclusters whose centers are again determined by k-means. New subclusters are accepted if they improve the clustering quality accounting for the increased number of parameters. For this, we use the minimal noiseless description length criterion (57,58). We use an implementation of the X-means algorithm in Python (59) and use individual cell parameters as initial cluster centers (60). The X-means algorithm can converge to different final results depending on the initial cluster centers. Thus, we use all 59·58/2 possible combinations of initial cluster centers and use the result that occurs most often. We rescale each parameter by the median of its distribution.

### Markovian embedding

A similar kernel to Eq. 29, namely

(Equation 19) $$\Gamma \left(t\right)=2a\delta \left(t\right)+{be}^{-t/\tau }\left(\cos\left(\Omega t\right)+\frac{1}{\tau \Omega }\sin\left(\Omega t\right)\right)\text{,}$$

can be derived from a system of harmonically coupled degrees of freedom. In fact, Eq. 19 becomes equivalent to Eq. 29 if the oscillation period 1/Ω is much smaller than the decay time *τ*, which is the case for the extracted algae kernels. The Hamiltonian describing the coupled degrees of freedom takes the form

(Equation 20) $$H=\frac{m}{2}v^{2}+\frac{m_{y}}{2}v_{y}^{2}+\frac{K}{2}{\left(x-y\right)}^{2}\text{,}$$

where $m_{i}$ are the effective masses of the two degrees of freedom and K=bm is the harmonic coupling strength. In the presence of friction, quantified by friction coefficients $\gamma_{i}$, and coupling the degrees of freedom to a heat bath at temperature *T*, the coupled equations of motion are given by

(Equation 21) $$\begin{array}{l} \dot{x}\left(t\right)=v\left(t\right)\\ m\dot{v}\left(t\right)=-\gamma_{x}v+bm\left(y\left(t\right)-x\left(t\right)\right)+F_{Rx}\left(t\right)\\ \dot{y}\left(t\right)=v_{y}\left(t\right)\\ m_{y}{\dot{v}}_{y}\left(t\right)=-\gamma_{y}v_{y}+bm\left(x\left(t\right)-y\left(t\right)\right)+F_{Ry}\left(t\right)\text{,} \end{array}$$

where $F_{Rx}\left(t\right)$ and $F_{Ry}\left(t\right)$ are random forces with zero mean and second moment $\left\langle F_{Ri}\left(0\right)F_{Rj}\left(t\right)\right\rangle =\delta_{ij}2\gamma_{i}k_{B}T\delta \left(t\right)$. In the supporting material, it is shown that the coupled equations of motion Eq. 21 are equivalent to a GLE in the form of Eq. 23 for the coordinate x(t) with a memory kernel Γ(t) given by Eq. 19 (61). The friction of the first degree of freedom leads to the *δ*-contribution of Γ(t) and the harmonic coupling to the second degree of freedom leads to the oscillating exponentially decaying contribution. The parameters of Eq. 21 translate into the parameters of Eq. 19 as

(Equation 22) $$\begin{array}{l} a=\frac{\gamma_{x}}{m}\\ \tau =2\frac{m_{y}}{\gamma_{y}}\\ \Omega =\sqrt{\frac{bm}{m_{y}}-\frac{1}{\tau^{2}}}\;\text{.} \end{array}$$

## Results

### Experimental trajectories

Our analysis is based on videos of 59 CR cells that are strongly confined between two glass plates separated by a distance similar to the cell diameter ∼10μm, which resembles the natural habitat of CR in soil (45) and simplifies the recording of long two-dimensional trajectories, as the cells cannot move out of the image plane of the microscope objective (see sections “cell growth and sample preparation” and “recording of trajectories” for experimental details). Videos that resolve the flagella motion are shown in the supporting material and reveal two different types of flagellar motion (51). In one type, the flagella move synchronously as in a breaststroke called “synchro” (Fig. 1 *a* and Video S1); in the other type, the flagella move asynchronously, which results in a wobbling cell motion called “wobbler” (Fig. 1 *b* and Video S2). The emergence of two different swimming modes reflects cell-size variation and constitutes the tactile cell response to the confining surfaces (51), where the synchros tend to exhibit slightly larger cell bodies and therefore are more strongly confined. The confining surfaces are coated with an anti-adhesive polymer brush to prevent sticking of cells to the surfaces. The strong confinement leads to cell-surface friction, which is fully accounted for by the GLE analysis. In fact, unconfined CR cells do not exhibit distinct synchro and wobbler swimming modes (51,52). Switching events between synchronous and asynchronous flagella motion are never observed and thus are negligible. We use the classification into synchros and wobblers based on the flagella motion in the high-resolution video data as a test of our classification method that is based on the cell-center trajectories.

Video S1. A representative synchro High-speed video microscopy at 500 frames per second obtained by phase-contrast imaging of a synchro CR cell showing the planar and synchronous breaststroke motion of the flagella. Between t ≈ 330–390 ms the synchronous beat of the flagella exhibits a phase slip, meaning the synchronicity of the flagella is disturbed in that short time interval.

Video S2. A representative wobbler High-speed video microscopy at 500 frames per second obtained by phase-contrast imaging of a CR cell that paddles the flagella in an asynchronous and irregular manner, resulting in the wobbling motion of the cell body.

### Theoretical trajectory model

The experiments yield two-dimensional trajectories x(t),y(t) for the cell center position, which we describe by the GLE

(Equation 23) $$\ddot{x}\left(t\right)=-\int_{t_{0}}^{t}\Gamma_{v}\left(t-t^{\prime }\right)\dot{x}\left(t^{\prime }\right)dt^{\prime }+F_{R}\left(t\right)$$

with an identical equation for y(t). Here, $\ddot{x}\left(t\right)=\dot{v}\left(t\right)$ denotes the acceleration of the cell position, $\Gamma_{v}\left(t\right)$ is a memory kernel that describes how the acceleration at time *t* depends on the cell velocity $\dot{x}\left(t^{\prime }\right)=v\left(t^{\prime }\right)$ at previous times and therefore accounts for non-Markovian friction effects, and $F_{R}\left(t\right)$ is a random force that describes interactions with the surrounding and within the interior of the cell. Since the experimental system is isotropic and homogeneous in space, no deterministic force term appears in the GLE. In fact, the GLE in Eq. 23 can be derived by projection at time $t_{0}$ from the underlying many-body Hamiltonian even in the presence of nonequilibrium effects, which obviously are present for living organisms (33,34,35,37).

If the cell motion can be described as a Gaussian process, which for CR cells is suggested by the fact that the single-cell velocity distributions are perfectly Gaussian, as will be demonstrated further below, the random force is a Gaussian process with correlations given by

(Equation 24) $$\left\langle F_{R}\left(t\right)F_{R}\left(0\right)\right\rangle =B\Gamma_{R}\left(t\right)\text{,}$$

where $B=\left\langle v^{2}\right\rangle$ denotes the mean-squared cell velocity and the symmetric random-force kernel is denoted as $\Gamma_{R}\left(t\right)$. In this case, the equation of motion is linear and there is no coupling between the motion in *x* and *y* direction, and we thus average all cell-trajectory data over the two directions.

For an equilibrium system, the fluctuation dissipation theorem (FDT) predicts $\Gamma_{R}\left(t\right)=\Gamma_{v}\left(\left|t\right|\right)$ with the mean-squared velocity given by $B=k_{B}T/m$ according to the equipartition theorem, where *m* is the mass of the moving object and $k_{B}T$ denotes the thermal energy (33,34). For living cells, both FDT and equipartition theorem do not hold in general and thus there is no a priori reason why $\Gamma_{v}\left(\left|t\right|\right)$ and $\Gamma_{R}\left(t\right)$ should be equal (36,62). Nevertheless, one can construct a surrogate model with an effective kernel $\Gamma \left(\left|t\right|\right)=\Gamma_{R}\left(t\right)=\Gamma_{v}\left(\left|t\right|\right)$ that exactly reproduces the dynamics described by the nonequilibrium GLE with $\Gamma_{R}\left(t\right)\neq \Gamma_{v}\left(\left|t\right|\right)$. This can be most easily seen by considering the VACF defined by

(Equation 25) $$C_{vv}\left(t\right)=\left\langle v\left(0\right)v\left(t\right)\right\rangle \text{,}$$

whose Fourier transform ${\tilde{C}}_{vv}\left(\omega \right)=\int_{-\infty }^{\infty }e^{-i\omega t}C_{vv}\left(t\right)dt$ follows from Eqs. 23 and 24 as (30)

(Equation 26) $${\tilde{C}}_{vv}\left(\omega \right)=\frac{B{\tilde{\Gamma }}_{R}\left(\omega \right)}{\left({\tilde{\Gamma }}_{v}^{+}\left(\omega \right)+i\omega \right)\left({\tilde{\Gamma }}_{v}^{+}\left(-\omega \right)-i\omega \right)}\text{,}$$

where ${\tilde{\Gamma }}_{v}^{+}\left(\omega \right)$ denotes the single-sided Fourier transform of $\Gamma_{v}\left(t\right)$ (see section Velocity autocorrelation function for the derivation). The VACF of the surrogate model with $\Gamma \left(\left|t\right|\right)=\Gamma_{R}\left(t\right)=\Gamma_{v}\left(\left|t\right|\right)$ follows from Eq. 26 as

(Equation 27) $${\tilde{C}}_{vv}^{\text{sur}}\left(\omega \right)=\frac{B\tilde{\Gamma }\left(\omega \right)}{\left({\tilde{\Gamma }}^{+}\left(\omega \right)+i\omega \right)\left({\tilde{\Gamma }}^{+}\left(-\omega \right)-i\omega \right)}\text{.}$$

For each combination of $\Gamma_{R}\left(t\right)$ and $\Gamma_{v}\left(t\right)$, there is a unique Γ(t) that produces the same VACF; i.e., for which ${\tilde{C}}_{vv}^{\text{sur}}\left(\omega \right)={\tilde{C}}_{vv}\left(\omega \right)$ holds (see the supporting material for the derivation) and which can be uniquely extracted from trajectories via the VACF (as shown in section “memory kernel extraction”). Since the VACF completely determines the dynamics of a Gaussian system, as shown in section “two-point velocity distribution” and in more detail in the supporting material, this implies that the extracted effective kernel Γ(t) not only describes the VACF exactly but also characterizes the system completely (63). In fact, recent work, where the nonequilibrium GLE is derived from a suitably chosen time-dependent Hamiltonian, shows that, for Gaussian nonequilibrium observables, the condition $\Gamma_{R}\left(t\right)=\Gamma_{v}\left(\left|t\right|\right)$ is actually satisfied (37), in line with our method that is based on extracting an effective kernel Γ(t).

### Trajectories and velocity distributions

**Figure 2:**
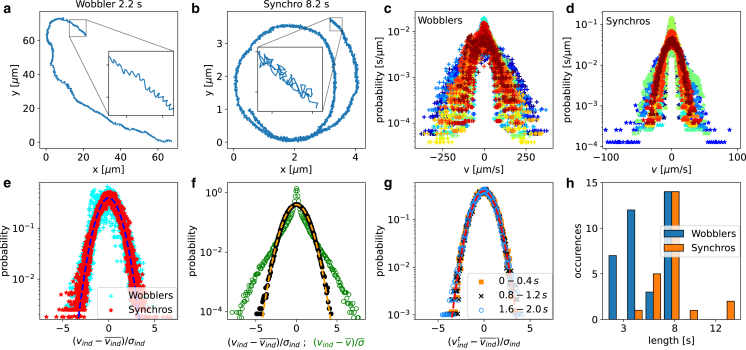
Cell-center trajectories. Exemplary cell-center trajectory (54) (*a*) of a wobbler of duration 2.2s and (*b*) of a synchro of duration 8.2s. The insets show trajectory fragments of duration 0.2s each. Velocity distributions of (*c*) wobblers and (*d*) synchros, individual cells are distinguished by color. (*e*) Velocity distributions of individual cells rescaled by subtracting the mean velocity of individual cells $\bar{v_{\text{ind}}}$ and dividing by their standard deviation $\sigma_{\text{ind}}$ for wobblers (*cyan crosses*) and synchros (*red stars*). The dashed line is the normal distribution. (*f*) Mean velocity distribution averaged over all cells (wobblers and synchros). For the green circles, the cell velocities are rescaled by subtracting the ensemble mean velocity $\bar{v}$ and dividing by the ensemble standard deviation $\bar{\sigma }$; for the black circles, the cell velocities are rescaled by subtracting the mean velocity of individual cells $\bar{v_{\text{ind}}}$ and dividing by their standard deviation $\sigma_{\text{ind}}$ as in (*e*). The normal distribution is indicated by a dashed line. (*g*) Individually rescaled velocity distributions of all cells for three different time windows. (*h*) Distribution of recorded trajectory lengths for wobblers and synchros. To see this figure in color, go online.

Due to the asynchronous flagella motion of the wobblers, the cells turn in the flagella-beating rhythm and exhibit monotonically forward-moving wiggly trajectories, as shown in Fig. 2 *a*. In contrast, the synchronous flagella beating of synchros leads to fast switching between forward and backward motion, as shown in Fig. 2 *b*. As a consequence, synchros exhibit much slower net-forward motion than wobblers, as seen in Fig. 2 *a* and *b*, where trajectories with total duration 2.2s (wobbler) and 8.2s (synchro) are compared. Synchros also exhibit a somewhat narrower instantaneous velocity distribution, as seen in Fig. 2 *c* and *d*. However, a differentiation of the two cell types solely based on their speed does not work, as we will show later.

Even though individual cells exhibit pronounced variations in their velocity distributions, as seen from the large spread in Fig. 2 *c* and *d*, their velocity distributions are Gaussian, as demonstrated in Fig. 2 *e*: When subtracting from the cell velocities the mean velocity of each individual cell and dividing by the corresponding velocity standard deviation, $\left(v_{\text{ind}}-\bar{v_{\text{ind}}}\right)/\sigma_{\text{ind}}$, all individual velocity distributions collapse onto the Gaussian (normal) distribution (dashed line in Fig. 2 *e*). In contrast, when subtracting from the cell velocities the cell-ensemble mean velocity and dividing by the cell-ensemble standard deviation, $\left(v_{\text{ind}}-\bar{v}\right)/\sigma$, the velocity distribution averaged over all cells deviates strongly from a Gaussian (green circles in Fig. 2 *f*). In contrast, the individually rescaled velocity distribution averaged over all cells (black dots) perfectly agrees with the Gaussian normal distribution (dashed line in Fig. 2 *f*). Thus, single cells exhibit perfectly Gaussian velocity distributions, which suggests that the GLE with Gaussian noise is appropriate to analyze experimental single-cell trajectories and that the condition $\Gamma_{R}\left(t\right)=\Gamma_{v}\left(\left|t\right|\right)$ holds (37).

The GLE in Eq. 23 features time-independent parameters and thus describes a stationary process. That the cell velocity distribution does not change over the observational time is demonstrated in Fig. 2 *g*, where velocity distributions in three consecutive time intervals are compared (again subtracting the individual cell mean velocities and dividing by the corresponding velocity deviations of the entire trajectory). This suggests that the motion of individual CR algae can indeed be modeled by the GLE in Eq. 23. In this context, it is to be noted that wobblers are relatively fast and tend to move out of the camera window more quickly than synchros, leading to slightly shorter wobbler trajectories, as shown in Fig. 2 *h*.

### Trajectory analysis and friction-kernel extraction

Trajectories are standardly characterized by the VACF or by the MSD

(Equation 28) $$C_{\text{MSD}}\left(t\right)=\left\langle {\left(x\left(0\right)-x\left(t\right)\right)}^{2}\right\rangle \;\text{.}$$

**Figure 3:**
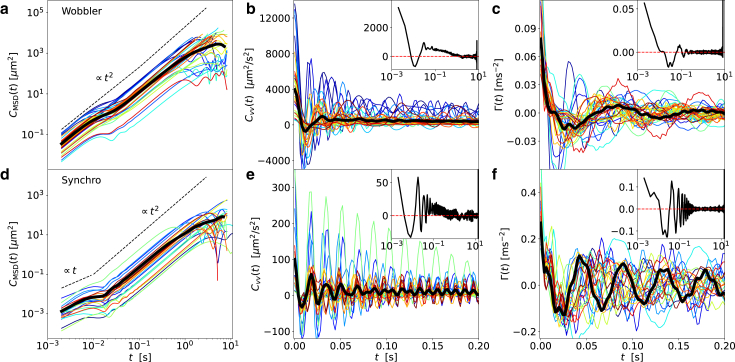
Results for the MSD, the VACF, and the friction kernel for wobblers and synchros. Results for the MSD, $C_{\text{MSD}}\left(t\right)$ defined in Eq. 28; the VACF, $C_{vv}\left(t\right)$ defined in Eq. 24; and the friction kernel Γ(t), extracted according to Eq. 8, for wobblers in (*a*)–(*c*) and synchros in (*d*)–(*f*). Different colors represent results for individual cells; the black lines in (*a*), (*b*), (*d*), and (*e*) denote the average over all cells. For the friction kernels, the black line is computed from the average VACF. The dashed lines in (*a*) and (*d*) indicate ballistic and diffusive scaling. Insets show the long-time behavior of the average quantities on a lin-log scale. To see this figure in color, go online.

Although the VACF is simply the curvature of the MSD, $C_{vv}\left(t\right)=\frac{1}{2}\frac{d^{2}}{dt^{2}}C_{\text{MSD}}\left(t\right)$, the MSD and the VACF highlight different aspects of the trajectories. In fact, the different propulsion modes of wobblers and synchros lead to drastically different MSDs: the wobblers exhibit ballistic behavior $C_{\text{MSD}}\left(t\right)\propto t^{2}$ on both short and long timescales (Fig. 3 *a*) with an intermediate crossover at t∼0.02s. In contrast, the forward-backward motion of the synchros leads to short-time diffusive behavior $C_{\text{MSD}}\left(t\right)\propto t$ up to t∼0.01s followed by a long-time ballistic regime for t>0.02s (Fig. 3 *d*). The transition to the intermediate ballistic regime occurs for both synchros and wobblers around the flagella oscillation period of the order of t∼0.02s; the MSD for shorter times is dominated by the flagella motion and for longer times by the ballistic net-forward motion. The transition to asymptotic diffusive behavior, expected for long times, is for the synchros observed for t>2s in extended low-resolution microscopy data, whereas wobblers stay in the ballistic regime for the entire observation time of tens of seconds (see the supporting material). As wobblers move faster than synchros, their absolute VACF values are higher compared to the synchros, as seen in Fig. 3 *b* and *e*. The synchros exhibit less variation of the VACF among individual cells, which leads to slowly decaying oscillations in the VACF averaged over all cells (black line in Fig. 3 *e* compared to Fig. 3 *b*). These oscillations reflect the flagella-beating cycle.

We extract effective friction kernels Γ(t) from the VACF of individual cells, as described in section “memory kernel extraction.” The results, shown as colored lines in Fig. 3 *c* and *f*, demonstrate that the algal motion deviates strongly from the simple persistent random walk model, which is widely used to describe the motion of cells (28,29,32) and which in the GLE formulation would correspond to Γ(t) exhibiting a delta peak at t=0 and otherwise being zero. The extracted memory kernels also reveal a substantially higher friction for synchros compared to wobblers, in line with the fact that synchros are larger and thus interact more strongly with the confining surfaces.

Comparing Fig. 3 *b* with Fig. 3 *c* or Fig. 3 *e* with Fig. 3 *f*, one notes that the oscillation period of the friction kernel is substantially longer than that of the VACF. The complex relation between the kernel and the VACF is discussed in the supporting material, where it is shown that the extracted values of the kernel decay time and oscillation amplitude achieve high directionality and speed of CR cells.

**Figure 4:**
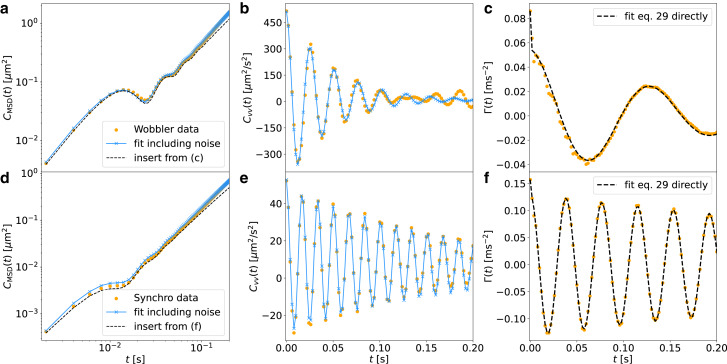
Check the accuracy of the GLE for a single wobbler and synchro. Results for the MSD, $C_{\text{MSD}}\left(t\right)$; VACF, $C_{vv}\left(t\right)$; and friction kernel Γ(t), of a single (*a*–*c*) wobbler and (*d*–*f*) synchro (*orange dots*). The black dashed lines in (*c*) and (*f*) denote fits of Eq. 29 to the extracted friction kernel determining the individual cell parameters shown in Fig. 5. The black dashed lines in (*a*) and (*d*) denote the analytical result Eq. S51 obtained from the friction-kernel fit in (*c*) and (*f*) and the mean-squared velocity $B=C_{vv}\left(0\right)$. Blue crosses in (*b*) and (e) denote a fit of the discretized expression for the VACF, including localization noise, (Eq. 17); blue crosses in (*a*) and (*d*) denote the corresponding prediction for the MSD, Eq. 15, using the same parameters as in (*b*) and (*e*); blue crosses are connected by blue straight lines. To see this figure in color, go online.

For both wobblers and synchros, the friction kernels exhibit an initial sharp peak followed by a decaying oscillation and are well described by

(Equation 29) $$\Gamma \left(t\right)=2a\delta \left(t\right)+{be}^{-t/\tau }\;\cos\left(\Omega t\right)\text{,}$$

with *δ*-peak amplitude *a*, oscillation amplitude *b*, exponential decay time *τ*, and oscillation frequency Ω. This is demonstrated in Fig. 4 *c* and *f* for one exemplary synchro and wobbler, where we compare the extracted memory kernels with fits according to Eq. 29, see section “fitting of friction kernel” for details. From these fits, we thus obtain four memory kernel parameters for each cell.

Before further analysis of the obtained individual cell parameters, we test whether the GLE Eq. 23 actually describes the cell motion. We thus compare the experimental MSD of a single wobbler and synchro in Fig. 4 *a* and *d* (orange dots) with the analytical prediction based on the GLE using the fit result for the friction kernel and the mean-squared velocity $B=C_{vv}\left(0\right)$ (black broken lines, the derivation of the analytical MSD expression is given in the supporting material). The agreement is very good, meaning that the memory extraction works well, which demonstrates that the GLE is an accurate model for the motion of organisms.

The MSD and VACF calculated from the GLE neglect the finite experimental recording time step of 0.002s, and they also neglect the localization noise of the cell position, due to the finite spatial resolution of the microscopy images and the projection of a three dimensional object onto a two-dimensional point position (30) (see section “discretized VACF including localization noise” for details). The blue crosses in Fig. 4 *b* and *e* represent fits of the GLE-based analytical expression for the VACF, which includes localization noise and discretization effects, given in Eq. 17, to the experimental data. The blue crosses in Fig. 4 *a* and *d* show the corresponding MSD results with the same parameters according to Eq. 15. The agreement between experimental data and the discretized model is perfect, and the fitted localization noise strength, defined in Eq. 15, is of the order of $\sigma_{\text{loc}}∼0.02\;\mu m$, similar to the pixel size, as expected (see discussion in the supporting material). This means that temporal and spatial discretization effects in the experimental data can be straightforwardly incorporated in the GLE model.

### Clustering of single-cell parameters

**Figure 5:**
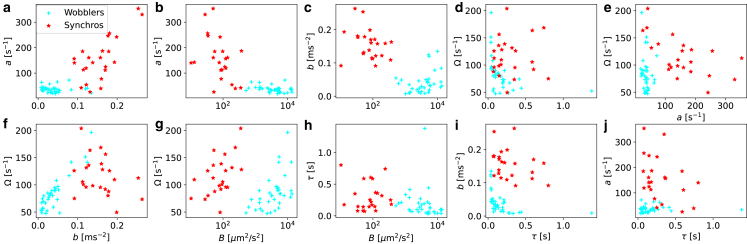
Scatter correlation plots of individual CR cell parameters. These consist of the friction-kernel parameters (*a*, *b*, *τ*, Ω) defined in Eq. 29 and the mean-squared velocity *B*. All parameters except *B* are presented on a linear scale. Synchros are shown in red and wobblers in cyan according to our cluster analysis, which perfectly matches a categorization based on the visual analysis of flagella motion. Error estimates of the extracted parameters are omitted here for clarity and shown in Fig. S5. To see this figure in color, go online.

The GLE Eq. 23 in conjunction with the random-force strength *B* defined in Eq. 24 and the effective friction kernel Eq. 29 has five parameters. This gives rise to 10 distinct two-dimensional projections, which are shown in Fig. 5. Each data point corresponds to a single cell. The parameters exhibit substantial spread among individual cells, but an unambiguous separation between wobblers and synchros, here colored in blue and red, is not obvious. As can already be seen in Fig. 3 *c* and *f*, the friction amplitudes *a* and *b* are larger for the synchros, whereas the mean-squared velocity *B* is larger for wobblers, which leads to a separation of the two populations in Fig. 5 *b* and *c*. Each flagellar beating cycle leads to a net forward cell motion, which is reflected by the positive correlation between memory oscillation frequency Ω and mean-squared velocity *B* in Fig. 5 *g* for each cell type. The uncertainty of the parameters in Fig. 5, estimated from the diagonal fit covariances, is rather low except for the decay time *τ*, as seen in Fig. S5 in the supporting material. Since the parameter *τ* contributes only marginally to the clustering confidence, as demonstrated by the results in Figs. 5 and S4, we conclude that our cluster analysis is not affected by parameter uncertainties.

We perform an unbiased cluster analysis using the X-means algorithm (55), which is a general version of the k-means algorithm (56) that self-consistently determines the optimal number of clusters (details are given in section “cluster analysis”). Applying this unbiased cluster analysis to the single-cell parameters in five-dimensional space, we obtain two distinct groups, which perfectly coincide with the assignment into wobblers and synchros from visual analysis of the flagellar motion in the video data (51). This means that we can classify the cells by just knowing their center of mass trajectories with an accuracy of 100%. In comparison, a cluster analysis solely based on the mean-squared velocity *B* leads to an accuracy of only 69%, whereas a cluster analysis using the first two principal-component analysis components leads to an accuracy of 90% (see the supporting material). Using only the four friction-kernel parameters for a cluster analysis without the mean-squared velocity *B* still reaches an accuracy of 92%. This clearly demonstrates that the GLE model, which parameterizes cell motion based on friction-kernel parameters in Eq. 29 together with the mean-squared velocity *B*, allows for accurate classification of cells based only on their motion.

## Discussion and conclusions

We demonstrate that the rather complex motion of individual CR algae can be accurately described by the GLE Eq. 23 and extract all GLE model parameters for individual algae from their cell-center trajectories in a data-driven manner. Based on the extracted GLE parameters, we detect two distinct algae classes by an unbiased cluster analysis; this unsupervised clustering result is confirmed by comparison with a categorization based on visual inspection of the flagella beating patterns. Our method is applicable to any kind of cells and even higher organisms if the motion is a Gaussian process. Cell and animal motion often exhibits aging effects (64), which in the GLE framework would show up as very slowly decaying contributions to the memory function. To accurately extract slowly decaying memory functions from data, very long trajectories would be needed. Since the GLE Eq. 23 is not restricted to positional degrees of freedom, our approach can be applied to any observable (for instance, cell extension or deformation). As the only inputs needed are trajectories, our method requires minimal interaction with the organisms and can be easily used as a stand-alone tool or to improve existing machine-learning algorithms for cell classification.

Additionally, our approach allows for a mechanistic interpretation of cell-motion characteristics. In fact, a friction-kernel model that is very similar to Eq. 29 and describes the data equally well can be derived from the equation of motion of two elastically coupled objects (see section “markovian embedding” and the supporting material for details). Without further experimental input, our approach does not reveal what these objects are, so we can only speculate that the elastic coupling between the cell body and the flagella, which presumably involves the connection between the flagellar basal bodies by the distal striated fiber (schematically shown in Fig. 1 *c*), causes the slowly decaying oscillations in the memory kernel. This seems in line with previous models for CR algae motion and flagella synchronization (65,66). Alternatively, the kernel oscillations could also be caused by some chemical or hydrodynamic feedback loop. Clearly, more experiments that resolve the relative motion of the cell center and the flagella are needed to resolve these issues.

Our extraction of GLE parameters from cell trajectories yields a single effective memory kernel and does not allow detection of the nonequilibrium character of cell motion, in agreement with recent general arguments (63). Conversely, based on an explicit nonequilibrium model for cell motion, it is rather straightforward to derive the GLE Eq. 23 and the functional form of the extracted kernel (Eq. 29), as shown in the supporting material. A similar GLE model can also be derived for motion in a confining potential to describe confined neurons (5) or bacteria moving in mucus (67) (see the supporting material for details).

As a final note, we mention that the angular orientation of CR algae can be accurately extracted from their cell-center trajectory, as shown in the supporting material. Thus, the orientational cell dynamics is included in our GLE model.

In summary, our approach allows for cell classification by positional cell-center trajectories or any other kind of time-series data and at the same time for interpretation of the motion pattern in terms of intracellular interactions. We anticipate numerous applications in biology and medicine that require the label-free distinction of individual cells and organisms.

## Author contributions

D.M. and P.S. designed and performed the experiments. S.B. contributed analytic tools and analyzed data. A.K. and R.R.N. designed the models, analyzed the experimental data, and wrote the manuscript.

## Supporting Material

Document S1. Figures S1–S9 and Equations S1–S91

Document S2. Article plus Supporting Material

## References

[1] Gessert N., Bengs M., Ellebrecht D.B. (2019). Deep transfer learning methods for colon cancer classification in confocal laser microscopy images. Int. J. Comput. Assist. Radiol. Surg..

[2] Teng M.W.L., Ngiow S.F., Smyth M.J. (2015). Classifying cancers based on T-cell infiltration and PD-L1. Cancer Res..

[3] Kang R., Park B., Chen K. (2020). Single-cell classification of foodborne pathogens using hyperspectral microscope imaging coupled with deep learning frameworks. Sensor. Actuator. B Chem..

[4] Davis R.O., Siemers R.J. (1995). Derivation and reliability of kinematic measures of sperm motion. Reprod. Fertil. Dev..

[5] Zeng H., Sanes J.R. (2017). Neuronal cell-type classification: challenges, opportunities and the path forward. Nat. Rev. Neurosci..

[6] Zhang L., Wang F., Zhu Y. (2020). Classifying Cell Types with DNA-Encoded Ligand–Receptor Interactions on the Cell Membrane. Nano Lett..

[7] Sanz I., Wei C., Lee F.E.-H. (2019). Challenges and opportunities for consistent classification of human B cell and plasma cell populations. Front. Immunol..

[8] Saliba A.-E., Westermann A.J., Vogel J. (2014). Single-cell RNA-seq: advances and future challenges. Nucleic Acids Res..

[9] Kolodziejczyk A.A., Kim J.K., Teichmann S.A. (2015). The technology and biology of single-cell RNA sequencing. Mol. Cell.

[10] Papalexi E., Satija R. (2018). Single-cell RNA sequencing to explore immune cell heterogeneity. Nat. Rev. Immunol..

[11] Qi R., Ma A., Zou Q. (2020). Clustering and classification methods for single-cell RNA-sequencing data. Briefings Bioinf..

[12] Chen C.L., Mahjoubfar A., Jalali B. (2016). Deep learning in label-free cell classification. Sci. Rep..

[13] Shen S., Syal K., Wang S. (2015). Note: An automated image analysis method for high-throughput classification of surface-bound bacterial cell motions. Rev. Sci. Instrum..

[14] Liu X., Chang Y.-T. (2022). Fluorescent probe strategy for live cell distinction. Chem. Soc. Rev..

[15] Sbalzarini I.F., Theriot J., Koumoutsakos P. (2002). Studying Turbulence Using Numerical Simulation Databases-IX: Proceedings of the 2002 Summer Program.

[16] Boedeker H.U., Beta C., Bodenschatz E. (2010). Quantitative analysis of random ameboid motion. Europhys. Lett..

[17] Pohl O., Hintsche M., Stark H. (2017). Inferring the chemotactic strategy of P. putida and E. coli using modified Kramers-Moyal coefficients. PLoS Comput. Biol..

[18] Maiuri P., Rupprecht J.-F., Voituriez R. (2015). Actin flows mediate a universal coupling between cell speed and cell persistence. Cell.

[19] Heuzé M.L., Vargas P., non-Lennon-Duménil A.M. (2013). Migration of dendritic cells: physical principles, molecular mechanisms, and functional implications. Immunol. Rev..

[20] Liu Y.-J., Le Berre M., Piel M. (2015). Confinement and low adhesion induce fast amoeboid migration of slow mesenchymal cells. Cell.

[21] da Silva C.L., Petry L.M., Bogorny V. (2019). 2019 8th Brazilian Conference on Intelligent Systems (BRACIS).

[22] Amselem G., Theves M., Beta C. (2012). A stochastic description of Dictyostelium chemotaxis. PLoS One.

[23] Selmeczi D., Li L., Flyvbjerg H. (2008). Cell motility as random motion: A review: Cell motility as random motion. Eur. Phys. J. Spec. Top..

[24] Selmeczi D., Mosler S., Flyvbjerg H. (2005). Cell motility as persistent random motion: theories from experiments. Biophys. J..

[25] Codling E.A., Plank M.J., Benhamou S. (2008). Random walk models in biology. J. R. Soc. Interface.

[26] Pedersen J.N., Li L., Flyvbjerg H. (2016). How to connect time-lapse recorded trajectories of motile microorganisms with dynamical models in continuous time. Phys. Rev. E.

[27] Dieterich P., Klages R., Schwab A. (2008). Anomalous dynamics of cell migration. Proc. Natl. Acad. Sci. USA.

[28] Gail M.H., Boone C.W. (1970). The locomotion of mouse fibroblasts in tissue culture. Biophys. J..

[29] Wright A., Li Y.-H., Zhu C. (2008). The differential effect of endothelial cell factors on in vitro motility of malignant and non-malignant cells. Ann. Biomed. Eng..

[30] Mitterwallner B.G., Schreiber C., Netz R.R. (2020). Non-Markovian data-driven modeling of single-cell motility. Phys. Rev. E.

[31] Li L., Cox E.C., Flyvbjerg H. (2011). ‘Dicty dynamics’: Dictyostelium motility as persistent random motion. Phys. Biol..

[32] Li L., Nørrelykke S.F., Cox E.C. (2008). Persistent cell motion in the absence of external signals: a search strategy for eukaryotic cells. PLoS One.

[33] Mori H. (1965). Transport, Collective Motion, and Brownian Motion. Prog. Theor. Phys..

[34] Zwanzig R. (1961). Memory effects in irreversible thermodynamics. Phys. Rev..

[35] Ayaz C., Scalfi L., Netz R.R. (2022). Generalized Langevin equation with a nonlinear potential of mean force and nonlinear memory friction from a hybrid projection scheme. Phys. Rev. E.

[36] Mizuno D., Tardin C., MacKintosh F.C. (2007). Nonequilibrium mechanics of active cytoskeletal networks. Science.

[37] Netz R.R. (2023). Derivation of the non-equilibrium generalized Langevin equation from a generic time-dependent Hamiltonian. arXiv.

[38] Bechinger C., Di Leonardo R., Volpe G. (2016). Active particles in complex and crowded environments. Rev. Mod. Phys..

[39] Viswanathan G.M., Da Luz M.G., Stanley H.E. (2011).

[40] Romanczuk P., Bär M., Schimansky-Geier L. (2012). Active Brownian particles: From individual to collective stochastic dynamics. Eur. Phys. J. Spec. Top..

[41] Ramaswamy S. (2010). The mechanics and statistics of active matter. Annu. Rev. Condens. Matter Phys..

[42] Brockmann D., Hufnagel L., Geisel T. (2006). The scaling laws of human travel. Nature.

[43] Tailleur J., Cates M.E. (2008). Statistical mechanics of interacting run-and-tumble bacteria. Phys. Rev. Lett..

[44] Mitterwallner B.G., Lavacchi L., Netz R.R. (2020). Negative friction memory induces persistent motion. Eur. Phys. J. E Soft Matter.

[45] Jeanneret R., Contino M., Polin M. (2016). A brief introduction to the model microswimmer Chlamydomonas reinhardtii. Eur. Phys. J. Spec. Top..

[46] Nguyen T.H., Kandel M., Popescu G. (2017). Halo-free phase contrast microscopy. Sci. Rep..

[47] Dutcher S.K., O’Toole E.T. (2016). The basal bodies of Chlamydomonas reinhardtii. Cilia.

[48] Wan K.Y., Goldstein R.E. (2016). Coordinated beating of algal flagella is mediated by basal coupling. Proc. Natl. Acad. Sci. USA.

[49] Wakabayashi K.-i., Ide T., Kamiya R. (2009). Calcium-dependent flagellar motility activation in Chlamydomonas reinhardtii in response to mechanical agitation. Cell Motil Cytoskeleton.

[50] Mondal D., Adhikari R., Sharma P. (2020). Internal friction controls active ciliary oscillations near the instability threshold. Sci. Adv..

[51] Mondal D., Prabhune A.G., Sharma P. (2021). Strong confinement of active microalgae leads to inversion of vortex flow and enhanced mixing. Elife.

[52] Goldstein R.E. (2015). Green Algae as Model Organisms for Biological Fluid Dynamics. Annu. Rev. Fluid Mech..

[53] Kreis C.T., Le Blay M., Bäumchen O. (2018). Adhesion of Chlamydomonas microalgae to surfaces is switchable by light. Nat. Phys..

[54] Blair D., Dufresne E. (2008). http://site.physics.georgetown.edu/matlab/code.html.

[55] Pelleg D., Moore A.W. (2000). X-means: Extending k-means with efficient estimation of the number of clusters. Icml.

[56] Bock H.-H. (2007). Selected Contributions in Data Analysis and Classification.

[57] Beheshti S., Dahleh M.A. (2005). A new information-theoretic approach to signal denoising and best basis selection. IEEE Trans. Signal Process..

[58] Shahbaba M., Beheshti S. (2012). 2012 11th International Conference on Information Science, Signal Processing and Their Applications (ISSPA).

[59] Novikov A. (2019). PyClustering: Data Mining Library. J. Open Source Softw..

[60] Celebi M.E., Kingravi H.A., Vela P.A. (2013). A comparative study of efficient initialization methods for the k-means clustering algorithm. Expert Syst. Appl..

[61] Brünig F.N., Geburtig O., Netz R.R. (2022). Time-dependent friction effects on vibrational infrared frequencies and line shapes of liquid water. J. Phys. Chem. B.

[62] Netz R.R. (2018). Fluctuation-dissipation relation and stationary distribution of an exactly solvable many-particle model for active biomatter far from equilibrium. J. Chem. Phys..

[63] Netz R.R. (2023). Multi-point distribution for Gaussian non-equilibrium non-Markovian observables. https://arxiv.org/abs/2310.08886.

[64] Metzler R., Jeon J.-H., Barkai E. (2014). Anomalous diffusion models and their properties: non-stationarity, non-ergodicity, and ageing at the centenary of single particle tracking. Phys. Chem. Chem. Phys..

[65] Friedrich B.M., Jülicher F. (2012). Flagellar synchronization independent of hydrodynamic interactions. Phys. Rev. Lett..

[66] Quaranta G., Aubin-Tam M.-E., Tam D. (2015). Hydrodynamics versus intracellular coupling in the synchronization of eukaryotic flagella. Phys. Rev. Lett..

[67] Wang B.X., Wu C.M., Ribbeck K. (2021). Home, sweet home: how mucus accommodates our microbiota. FEBS J..

