# Supplementary material for: Data-driven classification of individual cells by their non-Markovian motion
Source: Biophys J. 2024 Mar 21;123(10):1173–83. doi: 10.1016/j.bpj.2024.03.023 (PMC11140416; doi:10.1016/j.bpj.2024.03.023)
Supplement: Document S1. Figures S1–S9 and Equations S1–S91 [file mmc1.pdf]

Biophysical Journal, Volume *123*

Supplemental information

Data-driven classification of individual cells by their non-Markovian motion

Anton Klimek, Debasmita Mondal, Stephan Block, Prerna Sharma, and Roland R. Netz

Supplementary information: Data-driven classification of single cells by their non-Markovian motion

Contents

I Video captions **2**

II Effective kernel follows uniquely from correlation functions **2**

III Green's function is given in terms of positional two-point correlation function **2**

IV Long-time MSDs **4**

V Relation between the oscillation periods of the friction kernel and the VACF **5**

VI Derivation of the analytical expression for the MSD **6**

VII Localization noise fit **9**

VIII Cluster analysis in lower dimensions **10**

IX Comparing friction kernel expressions eq. (29) **and eq.** (19) **10**

X Exemplary non-equilibrium model describing CR motion **11**

XI Information on the cell orientation is contained in the cell-center trajectories **13**

XII Effects of smoothing trajectory data **14**

XIII Markovian embedding of the friction kernel eq. (19) **16**

I Video captions

Video 1.: A representative synchro. High-speed video microscopy at 500 frames per second obtained by phase-contrast imaging of a synchro CR cell showing the planar and synchronous breaststroke motion of the flagella. Between t $\sim$ $330 - 390\,ms$ the synchronous beat of the flagella exhibits a phase slip, meaning the synchronicity of the flagella is disturbed in that short time interval.

Video 2.: A representative wobbler. High-speed video microscopy at 500 frames per second obtained by phase-contrast imaging of a CR cell which paddles the flagella in an asynchronous and irregular manner resulting in the wobbling motion of the cell body.

II Effective kernel follows uniquely from correlation functions

We can write the GLE with an arbitrary potential $U(x(t))$ as

$$\ddot{x}(t) = -\nabla U(x(t)) - \int_{t_0}^{t} \Gamma_v(t-t')\dot{x}(t')dt' + F_R(t)\,. \tag{S1}$$

Multiplying eq. (S1) by $v(t_0)$, inserting $\Gamma_v(t) = \Gamma(t)$ and averaging the entire equation leads to the Volterra equation

$$\frac{d}{dt}C_{vv}(t) = -C_{v\nabla U}(t) - \int_0^t \Gamma(t-s)C_{vv}(s)ds\,, \tag{S2}$$

using that the random force is not correlated with the initial velocity at the projection time, i.e. $\langle v(t_0)F_R(t)\rangle = 0$ and setting $t_0 = 0$. Volterra equations are well-behaved and for given functions $\Gamma(t)$ and $C_{v\nabla U}(t)$ one can find a solution of eq. (S2) in terms of $C_{vv}(t)$. Inversely, for given correlation functions $C_{vv}(t)$ and $C_{v\nabla U}(t)$ one can solve eq. (S2) in terms of the friction kernel $\Gamma(t)$ by Laplace transformation. With the definition of the Laplace transform of a function $f(t)$

$$\hat{f}(q) = \int_0^\infty f(t)e^{-qt}dt, \tag{S3}$$

the unique solution for $\Gamma(t)$ in Laplace space is given by

$$\hat{\Gamma}(q) = -\frac{1}{\hat{C}_{vv}(q)}\left(q\hat{C}_{vv}(q) - C_{vv}(0) + \hat{C}_{v\nabla U}(q)\right)\,. \tag{S4}$$

The existence of a unique friction kernel for any given input correlation functions $C_{vv}(t)$ and $C_{v\nabla U}(t)$ assures that one can always find an effective friction kernel $\Gamma(t)$ that, when employed in the GLE, reproduces the two-point correlation functions. Thus, every non-equilibrium model described by eq. (23) with $\Gamma_R(t) \neq \Gamma_v(|t|)$ can be mapped on an effective model with $\Gamma(|t|) = \Gamma_R(t) = \Gamma_v(|t|)$, because the Green's function, which completely describes a Gaussian process, is solely given in terms of the positional two-point correlation function, see SI Sec. III.

III Green's function is given in terms of positional two-point correlation function

In the following we map a non-equilibrium process described by the GLE eq. (S1) with the random force fluctuations given by eq. (24) and a harmonic potential $U(x) = Kx^2/2$ on an effective model with an effective friction kernel $\Gamma(t)$ fulfilling $\langle F_R(0)F_R(t)\rangle = k_BT\Gamma(|t|)$ and an effective harmonic potential $U_{\text{eff}}(x) = K_{\text{eff}}x^2/2$. For this we show that the Green's function for the non-equilibrium system eqs. (S1), (24) is given in terms of the positional autocorrelation function.

We define the average over the random force by the path integral

$$\langle X\rangle = \mathcal{N}\int \mathcal{D}\tilde{F}_R(\cdot)X\exp\left(-\frac{1}{4\pi B}\int_{-\infty}^{\infty}d\omega\int_{-\infty}^{\infty}d\omega'\frac{\tilde{F}_R(\omega)\delta(\omega+\omega')\tilde{F}_R(\omega')}{\tilde{\Gamma}_R(\omega)}\right)\,. \tag{S5}$$

We also define the generating functional as

$$\begin{aligned}\mathcal{Z}[\tilde{h}] =& \mathcal{N}\int \mathcal{D}\tilde{F}_R(\cdot)\exp\left(-\frac{1}{4\pi B}\int_{-\infty}^{\infty}d\omega\frac{\tilde{F}_R(\omega)\tilde{F}_R(-\omega)}{\tilde{\Gamma}_R(\omega)} + \int_{-\infty}^{\infty}d\omega\tilde{h}(-\omega)\tilde{F}_R(\omega)\right)\\ =& \exp\left(2\pi B\int_{-\infty}^{\infty}d\omega\tilde{h}(\omega)\tilde{h}(-\omega)\tilde{\Gamma}_R(\omega)\right)\end{aligned} \tag{S6}$$

where $\tilde{h}(\omega)$ is a generating field. Equation (S6) has the functional derivatives

$$\frac{\delta \mathcal{Z}[\tilde{h}]}{\delta \tilde{h}(\omega)} |_{\tilde{h}=0} = \langle \tilde{F}_R(\omega) \rangle = 0 \tag{S7}$$

$$\frac{\delta^2 \mathcal{Z}[\tilde{h}]}{\delta \tilde{h}(\omega) \delta \tilde{h}(\omega')} |_{\tilde{h}=0} = \langle \tilde{F}_R(\omega) \tilde{F}_R(\omega') \rangle = 2\pi B \delta(\omega + \omega') \tilde{\Gamma}_R(\omega) \,, \tag{S8}$$

where $\tilde{\Gamma}_R(\omega) = \tilde{\Gamma}_R(-\omega)$. For harmonic potential $U(x) = Kx^2/2$ we can Fourier-transform the GLE eq. (S1) by which we obtain the form of eq. (S28) with the response function given by

$$\tilde{\chi}(\omega) = \Big(K - \omega^2 + i\omega \tilde{\Gamma}_v^+(\omega) \Big)^{-1} \,. \tag{S9}$$

The Green's function can be written as

$$G(x, t \mid x', t') = \frac{P(x, t; x', t')}{P(x', t')}, \tag{S10}$$

which is the ratio of the joint two-point probability distribution to find x at t and x' at t' and the base probability to find x' at t'. Those probabilities can be expressed as

$$P(x', t') = \langle \delta(x' - x(t')) \rangle \,, \; P(x, t; x', t') = \langle \delta(x' - x(t')) \delta(x - x(t)) \rangle \,. \tag{S11}$$

From eq. (S28) we can write the solution of the GLE eq. (S1) using the linear response function $\chi(t)$ as

$$x(t) = \int_{-\infty}^{\infty} \frac{d\omega}{2\pi} e^{i\omega t} \tilde{\chi}(\omega) \tilde{F}_R(\omega). \tag{S12}$$

Inserting this result into the probability distributions eq. (S11) we obtain

$$P(x', t') = \int_{-\infty}^{\infty} \frac{dp}{2\pi} e^{-ipx'} \left\langle \exp\left(ip \int_{-\infty}^{\infty} \frac{d\omega}{2\pi} \tilde{\chi}(\omega) \tilde{F}_R(\omega) e^{i\omega t'} \right) \right\rangle \tag{S13}$$

and

$$\begin{aligned} P(x, t; x', t') = \int_{-\infty}^{\infty} \frac{dp}{2\pi} \int_{-\infty}^{\infty} \frac{dq}{2\pi} e^{-iqx - ipx'} \Bigg\langle \exp\Bigg(& iq \int_{-\infty}^{\infty} \frac{d\omega}{2\pi} \tilde{\chi}(\omega) \tilde{F}_R(\omega) e^{i\omega t} \\ & + ip \int_{-\infty}^{\infty} \frac{d\omega}{2\pi} \tilde{\chi}(\omega) \tilde{F}_R(\omega) e^{i\omega t'} \Bigg) \Bigg\rangle. \end{aligned} \tag{S14}$$

By comparing eqs. (S13), (S14) to eq. (S6) we find the generating field $\tilde{h}(\omega)$ for the base probability $P(x', t')$

$$\tilde{h}_b(-\omega) = \frac{ip}{2\pi} \tilde{\chi}(\omega) e^{i\omega t'} \tag{S15}$$

and for the joint probability $P(x, t; x', t')$ we find

$$\tilde{h}_j(-\omega) = \frac{i}{2\pi} \tilde{\chi}(\omega) \left(q e^{i\omega t} + p e^{i\omega t'} \right) \,, \tag{S16}$$

where the subscript b stands for the base probability and j for the joint probability. Using that the position correlation function can be written as

$$C_{xx}(t - t') = B \int_{-\infty}^{\infty} \frac{d\omega}{2\pi} \tilde{\chi}(\omega) \tilde{\Gamma}_R(\omega) \tilde{\chi}(-\omega) e^{i\omega(t-t')}, \tag{S17}$$

we arrive at the Gaussian form

$$\begin{aligned} P(x', t') &= \int_{-\infty}^{\infty} \frac{dp}{2\pi} \exp\left(-C_{xx}(0) p^2 - ipx' \right) \\ &= \sqrt{\frac{1}{2\pi C_{xx}(0)}} \exp\left(-\frac{x'^2}{2C_{xx}(0)} \right) \end{aligned} \tag{S18}$$

and

$$P(x,t;x',0) = \int_{-\infty}^{\infty}\frac{dp}{2\pi}\int_{-\infty}^{\infty}\frac{dq}{2\pi}\exp\left(-\frac{q^2+p^2}{2}C_{xx}(0) - qpC_{xx}(t) - iqx - ipx'\right)$$
$$=2\sqrt{\frac{1}{2(C_{xx}(0)^2 - C_{xx}(t)^2)}}\exp\left(-\frac{(x'+x)^2}{4(C_{xx}(0)+C_{xx}(t))} - \frac{(x'-x)^2}{4(C_{xx}(0)-C_{xx}(t))}\right). \tag{S19}$$

For simplicity we set $t' = 0$ as the position correlation function only depends on the time difference $t - t'$. Inserting eqs. (S18) (S19) into eq. (S10), we finally obtain the position Green's function as a Gaussian

$$G(x,t \mid x',0) = \frac{\exp(-\frac{(x-\mu)^2}{2\sigma^2})}{\sqrt{2\pi\sigma^2}} \tag{S20}$$

with mean and standard deviation

$$\mu = \mu(x',t) = \frac{C_{xx}(t)}{C_{xx}(0)}x', \; \sigma^2 = \sigma^2(t) = C_{xx}(0)\left(1 - \frac{C_{xx}(t)^2}{C_{xx}(0)^2}\right). \tag{S21}$$

Thus the Green's function is entirely described in terms of the two-point positional correlation function $C_{xx}(t)$, note that $C_{vv}(t) = -\frac{d^2}{dt^2}C_{xx}(t)$.

We next calculate the effective harmonic potential strength K_{eff}. The condition to find an effective description of the GLE eq. (S1) arises from setting eq. (S17) equal to its effective version

$$\tilde{\chi}(\omega)\tilde{\Gamma}_R(\omega)\tilde{\chi}(-\omega) = \tilde{\chi}_{\text{eff}}(\omega)\tilde{\Gamma}(\omega)\tilde{\chi}_{\text{eff}}(-\omega), \tag{S22}$$

which assures that the effective model has the same Green's function as the non-equilibrium model, as shown by eqs. (S20) and (S21). Here, $\tilde{\chi}_{\text{eff}}(\omega)$ is the effective response function and $\Gamma(t)$ is the effective kernel. Inserting eq. (S9) into eq. (S22) leads to

$$\frac{\tilde{\Gamma}_R(\omega)}{(K - \omega^2 + i\omega\tilde{\Gamma}_v^+(\omega))(K - \omega^2 - i\omega\tilde{\Gamma}_v^+(-\omega))} = \frac{\tilde{\Gamma}(\omega)}{(K_{\text{eff}} - \omega^2 + i\omega\tilde{\Gamma}^+(\omega))(K_{\text{eff}} - \omega^2 - i\omega\tilde{\Gamma}^+(-\omega))}. \tag{S23}$$

Defining the potential of mean force for the effective system according to

$$U_{\text{eff}}(x) = -B\ln(p(x)) = K_{\text{eff}}x^2/2, \tag{S24}$$

with $p(x)$ being the probability to observe x, and comparing eq. (S24) to the long time limit of the non-equilibrium distribution eq. (S20), namely

$$p(x) = \lim_{t\to\infty} G(x,t \mid x',0) = \frac{\exp(-\frac{x^2}{2C_{xx}(0)})}{\sqrt{2\pi C_{xx}(0)}}, \tag{S25}$$

we find the effective harmonic potential strength to be

$$K_{\text{eff}} = \frac{B}{C_{xx}(0)}. \tag{S26}$$

Note that the effective potential will in general deviate from the potential in the non-equilibrium GLE, i.e. $K_{\text{eff}} \neq K$. The present calculation can also be done for a general multi-point distribution (63).

IV Long-time MSDs

The trajectories analyzed in the main text (examples shown in Figs. 2a,b) are recorded with a 40 fold magnification at 500 frames per second, which leads to trajectories of lengths up to $14\,\text{s}$ (Fig. 2h). Here, we analyze a different data set to resolve the long time behavior of the MSD of the CR cells, which we obtain by using a lower magnification (tenfold) and longer time step of $0.02\,\text{s}$. A lower magnification results in a larger observation window, which in turn allows recording longer trajectories. The decreased localization precision and the longer time step do not allow to observe the details of the oscillatory features of the motion. Hence, we do not apply the cluster analysis relying on the extraction of the oscillating friction kernels,

but rather use an approximate distinction by the mean squared velocities B. We assign cells with $B > 110\,\mu\mathrm{m}^2/\mathrm{s}^2$ to be putative wobblers and cells with $B < 35\,\mu\mathrm{m}^2/\mathrm{s}^2$ to be putative synchros. In this way, we avoid the assignment of cells with intermediate mean squared velocities B, for which this approximate classification scheme is unreliable. For the slow cells we additionally exclude cells for which the MSD stays below $1\,\mu\mathrm{m}^2$ during the entire trajectory. These cells are most likely stuck or have some defects prohibiting them to move. This distinction into putative wobblers and synchros depends on our choice of the upper and lower bounds for B, which we choose according to the high resolution fastest synchro and slowest wobbler when using only every tenth step of the high resolution data, by which we obtain velocities with a time step of $0.02\,\mathrm{s}$. In Fig. S1 we demonstrate that the present approximate classification leads to extended MSDs (black lines) that match closely the MSDs shown in the main text based on the high-resolution videos (gray lines). The extended MSDs for synchros exhibit a transition from the ballistic to the long time diffusive regime at a time of about $t \approx 2$s, whereas for the wobblers the ballistic regime extends over more than ten seconds. The prefactor of the MSD in the diffusive regimes, i.e. the diffusivity D, is for the synchros in Fig. S1 given by $D = 9\,\mu\mathrm{m}^2/\mathrm{s}$. This transition to the long time diffusive regime is also indicated by the exemplary trajectories in Fig. S1c, where the synchro shows no directed motion on the time scale of the entire trajectory whereas the wobbler trajectory is still rather directed over the entire trajectory. Moreover, the higher speed of the wobblers is again reflected in higher MSDs for wobblers, as demonstrated in Fig. S1d.

V Relation between the oscillation periods of the friction kernel and the VACF

In Fig. 4 in the main text one can note a subtle difference between the oscillation period of the friction kernel and the oscillation period of the VACF when comparing Fig. 4b to 4c or Fig. 4e to 4f. Since we derive an analytical expression for the VACF in eq. (S52) for the friction kernel given in eq. (29), we can evaluate the dependence of the VACF oscillation frequency on the kernel frequency Ω. We are interested in the dominant oscillatory behavior at short times, therefore we consider the pole ω_i from eq. (S52) with the smallest real part, where we call this real part ω_{vv}. In Fig. S2a we show the dependence of ω_{vv} on Ω for different b and find the VACF frequency ω_{vv} to be constant for small kernel frequencies Ω followed by a transition to a regime where ω_{vv} equals Ω above a threshold value given by $\Omega_t = \omega_{vv}(\Omega \to 0)$. This threshold value depends on the kernel parameters a, b, τ and has the asymptotic behavior

$$\Omega_t = \begin{cases} \sqrt{b} & ,\text{for } b > \max(a^2/4, \tau^{-2}/4) \\ a/2 & ,\text{for } b < a^2/4 \wedge \tau > a^{-1} \\ \tau^{-1}/2 & ,\text{for } b < a^2/4 \wedge \tau < a^{-1}\,, \end{cases} \tag{S27}$$

which follows from Figs. S2a-c.

Since the median kernel frequency of the CR cells is of the order $\Omega \sim 100\,\tau^{-1}$ and the oscillation amplitude is of the order $b \sim 10^4\tau^{-2}$, the data lies in the transition regime between constant VACF frequency ω_{vv} and the linear regime $\omega_{vv} = \Omega$, as can be seen in Fig. S2a. From Fig. S2a we infer that the VACF frequency is always greater or equal to the kernel frequency, i.e. $\omega_{vv} \geq \Omega$. Moreover, the oscillation amplitudes of the CR cells usually fulfill $b > a^2/4$ and the inverse decay time τ^{-1} is small compared to the other kernel parameters, such that $a > \tau^{-1}$ and $b > \tau^{-2}/4$ are always fulfilled. Therefore, the cells exhibit parameters for which $b > \max(a^2/4, \tau^{-2}/4)$ holds. Eq. (S27) thus tells us that the threshold frequency is given by $\Omega_t = \sqrt{b}$. Combining this knowledge with $\omega_{vv} \geq \Omega$ leads to $\omega_{vv} \geq \sqrt{b}$. Since the CR data lies in the transition regime between $\omega_{vv} = \sqrt{b}$ and $\omega_{vv} = \Omega$, the VACF frequency ω_{vv} is strongly influenced by the oscillation amplitude b. In order to further estimate the exhibited parameter range, the reader is referred to Fig. 5 of the main text, which includes all parameters of all cells.

The fact that the VACF frequency ω_{vv} increases with increasing kernel oscillation amplitude b can be interpreted in the context of the Markovian embedding model in Sec. XIII, where the coupling strength of the harmonically coupled reaction coordinates is proportional to the oscillation amplitude b, as shown in eqs. (S75), (S77), which leads to faster oscillations for higher b. Since every oscillation leads to a net forward motion of the cell, it leads to fast net forward motion for cells if they exhibit a large kernel oscillation amplitude b compared to the squared inverse decay time τ^{-2} and the squared δ-amplitude a^2, which is the case in our data set (see Fig. 5). By realizing that the decay time τ is related to how long a cell 'remembers' its past trajectory, one can conclude that a fast decay of the oscillation would lead to a cell not being able to maintain its direction for a long time. Thus, it is useful to achieve fast net forward motion if the decay time is longer than the oscillation period, i.e. if $\tau\Omega > 1$. By net forward motion we mean directed motion on relevant time scales, since of course in the long-time limit the cells exhibit diffusion as shown in Fig. S1. Both criteria, the oscillation amplitude being large, i.e. $b > \max(\tau^{-2}, a^2)$ and the decay time being long compared to the oscillation period, i.e. $\tau\Omega > 1$, are fulfilled by the CR cell parameters shown in Fig. 5, which means that CR cells exhibit a range of kernel parameters that is suited to achieve fast net forward motion.

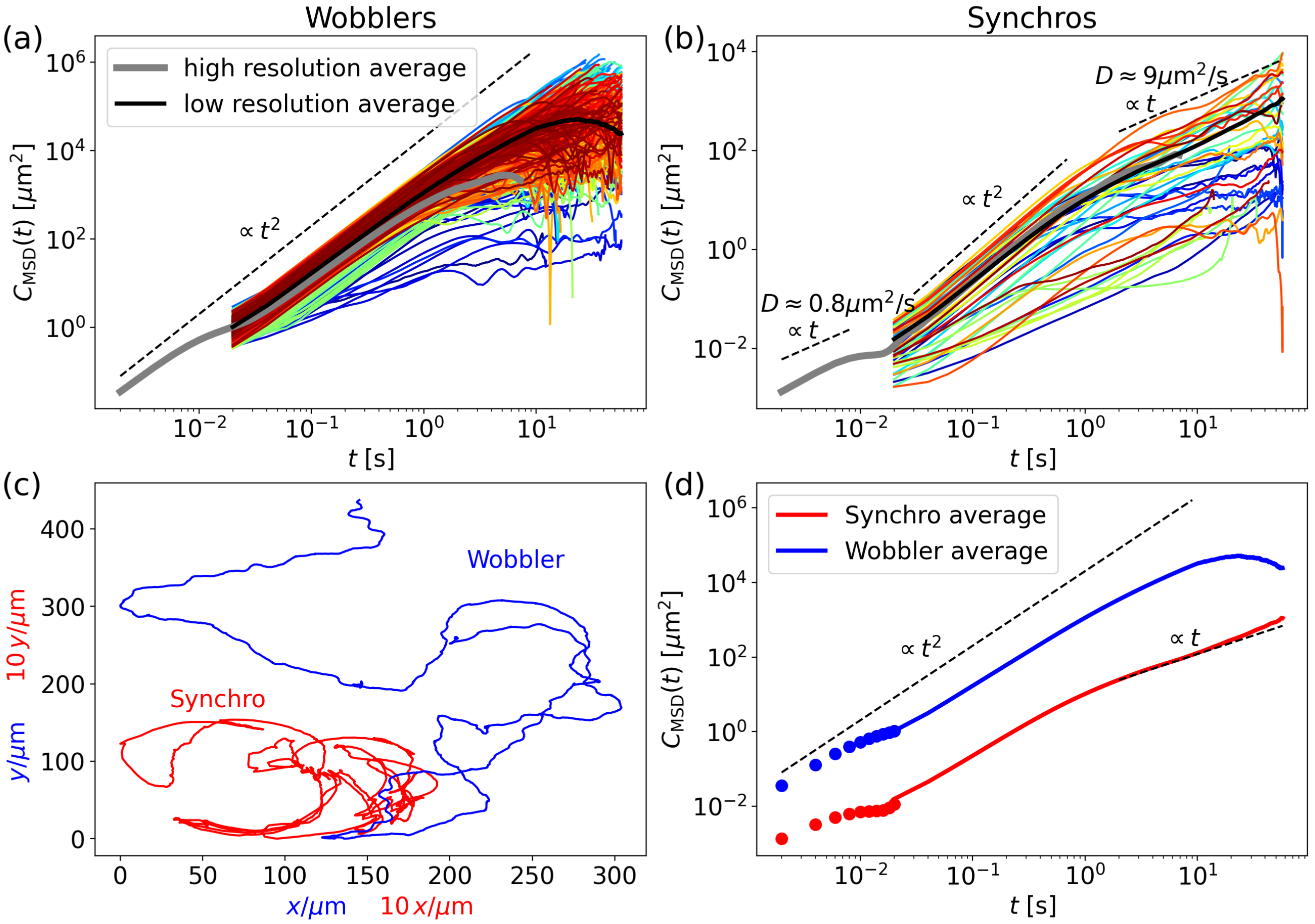

Figure S1: MSDs of individual cells (colored lines) from low-resolution data with 10x magnification and a time step of $\Delta = 0.02\,\mathrm{s}$ of (a) wobblers and (b) synchros according to an approximate classification scheme explained in the text. The average of the low-resolution MSD data is shown in black, the grey line represents the average of the MSD from Fig. 3 in the main text with higher temporal and spatial resolution of 40x and $\Delta = 0.002\,\mathrm{s}$. (c) Representative trajectories of the low resolution data of a wobbler in blue of length $57.1\,\mathrm{s}$ and of a synchro in red of length $50.9\,\mathrm{s}$, where the synchro positions are multiplied by a factor of ten to show them on the same scale with the wobbler trajectory. (d) The concatenated average MSD of short times from high resolution data as points and long times from low resolution data as solid lines for synchros in red and wobblers in blue.

VI Derivation of the analytical expression for the MSD

In Fourier space the GLE eq. (23) can be written as

$$\tilde{x}(\omega) = \tilde{\chi}(\omega)\tilde{F}_R(\omega)\,, \tag{S28}$$

where $\tilde{\chi}(\omega)$ is the Fourier transform of the position response function, which takes the form

$$\tilde{\chi}(\omega) = \left(-\omega^2 + i\omega\tilde{\Gamma}_v^+(\omega)\right)^{-1} \tag{S29}$$

with $\tilde{\Gamma}_v^+(\omega)$ being the half sided Fourier transform of the friction kernel defined via

$$\tilde{\Gamma}_v^+(\omega) = \int_0^\infty e^{-i\omega t}\Gamma_v(t)dt\,. \tag{S30}$$

The Fourier transform of the position correlation function $C_{xx}(t) = \langle x(0)x(t)\rangle$ can be written as

$$\tilde{C}_{xx}(\omega) = B\tilde{\chi}(\omega)\tilde{\Gamma}_R(\omega)\tilde{\chi}(-\omega)\,, \tag{S31}$$

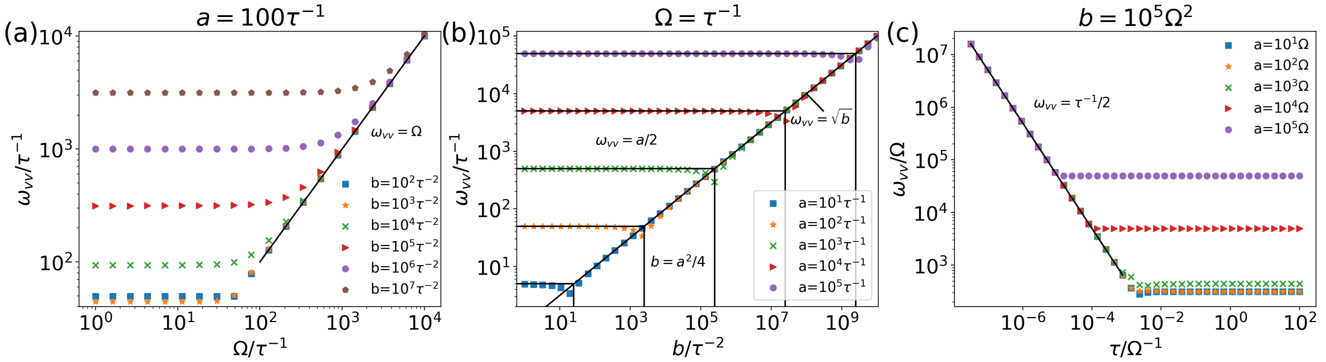

Figure S2: (a) The dependence of the VACF frequency ω_{vv} on the kernel frequency Ω of the kernel eq. (29) is shown for different kernel oscillation amplitudes b and fixed δ-peak amplitude $a = 100\tau^{-1}$. The black line indicates the linear behavior $\omega_{vv} = \Omega$. (b) The dependence of the VACF frequency ω_{vv} on the kernel oscillation amplitude b is shown for varying kernel δ-peak amplitude a and $\Omega = \tau^{-1}$, which represents the low kernel frequency plateau value shown in (a) for small Ω. The horizontal lines represent $\omega_{vv} = a/2$ for the respective values of a, the diagonal line represents $\omega_{vv} = \sqrt{b}$ and the vertical lines represent the intersection of the other lines, which mark a minimum in the parameter b positioned at $b = a^2/4$. (c) The dependence of the VACF frequency ω_{vv} on the kernel decay time τ shown for different δ-peak amplitudes a and fixed $b = 10^5\Omega^2$. The regime shown for τ/Ω^{-1} represents the low kernel frequency plateau value shown in (a) for small $\Omega\tau < 100$. The black line indicates the scaling $\omega_{vv} = \tau^{-1}/2$.

where we made use of

$$\begin{aligned}\tilde{C}_{xx}(\omega) &= \int_{-\infty}^{\infty} dt e^{-i\omega t}\langle x(0)x(t)\rangle \\ &= \int_{-\infty}^{\infty} e^{-i\omega t}dt \int_{\infty}^{\infty} e^{i\omega t}\frac{d\omega}{2\pi}\int_{-\infty}^{\infty}\frac{d\omega'}{2\pi}\langle\tilde{x}(\omega)\tilde{x}(\omega')\rangle\end{aligned} \tag{S32}$$

and

$$\langle \tilde{F}_R(\omega)\tilde{F}_R(\omega')\rangle = 2\pi B\delta(\omega+\omega')\tilde{\Gamma}_R(\omega')\,. \tag{S33}$$

Equation S31 can be rewritten as

$$\tilde{C}_{xx}(\omega) = \frac{B}{i\omega}\left(\tilde{\chi}(\omega) - \tilde{\chi}(-\omega)\right)\frac{\tilde{\Gamma}_R(\omega)}{\tilde{\Gamma}_v(\omega)}\,, \tag{S34}$$

which leads to the MSD

$$\begin{aligned}C_{\mathrm{MSD}}(t) &= 2(C_{xx}(0) - C_{xx}(t)) \\ &= B\int_{-\infty}^{\infty}\frac{d\omega}{\pi}\frac{e^{i\omega t}-1}{i\omega}\left(\tilde{\chi}(\omega)-\tilde{\chi}(-\omega)\right)\frac{\tilde{\Gamma}_R(\omega)}{\tilde{\Gamma}_v(\omega)}\,.\end{aligned} \tag{S35}$$

For the decaying oscillation model friction kernel of eq. (29) and using the effective form $\Gamma(|t|) = \Gamma_R(t) = \Gamma_v(|t|)$ one obtains the half-sided Fourier transform of the kernel as

$$\tilde{\Gamma}^{+}(\omega) = \int_0^{\infty}\left(2a\delta(t) + be^{-t/\tau}\cos(\Omega t)\right)e^{-i\omega t}dt = a + b\left(\frac{i\omega+\frac{1}{\tau}}{\Omega^2+(i\omega+\frac{1}{\tau})^2}\right)\,. \tag{S36}$$

Inserting the result of eq. (S36) into eq. (S29) we find the response function

$$\tilde{\chi}(\omega) = \left(-\omega^2 + i\omega a + i\omega b\left(\frac{i\omega+\frac{1}{\tau}}{\Omega^2+(i\omega+\frac{1}{\tau})^2}\right)\right)^{-1}\,, \tag{S37}$$

which by inserting into eq. (S35) leads to

$$C_{\mathrm{MSD}}(t) = B\int_{-\infty}^{\infty}\frac{d\omega}{\pi}\frac{(e^{i\omega t}-1)(k_1+k_2\omega^2+k_3\omega^4)}{\omega^2(c_1+c_2\omega^2+c_3\omega^4+\tau^4\omega^6)}\,, \tag{S38}$$

where the constants c_i and k_i are given by

$$\begin{aligned}
c_1 &= (a + b\tau + a\Omega^2\tau^2)^2 && \text{(S39)}\\
c_2 &= (1 + 2(a^2 - b + \Omega^2)\tau^2 + 2ab\tau^3 + (-2a^2\Omega^2 + (b + \Omega^2)^2)\tau^4) && \text{(S40)}\\
c_3 &= \tau^2(2 + (a^2 - 2(b + \Omega^2))\tau^2) && \text{(S41)}\\
k_1 &= -2(1 + \Omega^2\tau^2)(a + b\tau + a\Omega^2\tau^2) && \text{(S42)}\\
k_2 &= -2\tau^2(b\tau + a(2 - 2\Omega^2\tau^2)) && \text{(S43)}\\
k_3 &= -2a\tau^4 \,. && \text{(S44)}
\end{aligned}$$

Interpreting the integrand in eq. (S38) as a sum of the three terms each proportional to k_i, we see that all terms have the same poles, where the term proportional to k_1 has an additional double pole at $\omega = 0$. The solutions of ω^2 to the equation

$$c_1 + c_2\omega^2 + c_3\omega^4 + \tau^4\omega^6 = \tau^4(\omega^2 - \omega_1^2)(\omega^2 - \omega_2^2)(\omega^2 - \omega_3^2) = 0\,, \tag{S45}$$

define the remaining poles, which we denote by $\pm\sqrt{\omega_i^2}$ and which we compute by numerically solving eq. (S45). Next we use the partial fraction decompositions

$$\begin{aligned}
\frac{1}{\omega^2\prod_{i=1}^3(\omega^2 - \omega_i^2)} &= -\frac{1}{\omega^2\prod_{i=1}^3\omega_i^2} + \sum_{i=1}^3 \frac{1}{\omega_i^2(\omega^2 - \omega_i^2)\prod_{j\neq i}(\omega_i^2 - \omega_j^2)} && \text{(S46)}\\
\frac{1}{\prod_{i=1}^3(\omega^2 - \omega_i^2)} &= \sum_{i=1}^3 \frac{1}{(\omega^2 - \omega_i^2)\prod_{j\neq i}(\omega_i^2 - \omega_j^2)} && \text{(S47)}\\
\frac{\omega^2}{\prod_{i=1}^3(\omega^2 - \omega_i^2)} &= \sum_{i=1}^3 \frac{\omega_i^2}{(\omega^2 - \omega_i^2)\prod_{j\neq i}(\omega_i^2 - \omega_j^2)} && \text{(S48)}
\end{aligned}$$

to rewrite the fraction of eq. (S38) as a sum of terms proportional to $(\omega^2 - \omega_i^2)^{-1}$ and one term proportional to ω^{-2}. Using the solution of the integrals

$$\begin{aligned}
\int_{-\infty}^{\infty} \frac{e^{i\omega t} - 1}{\omega^2 - \omega_i^2} d\omega &= \frac{\pi(e^{-\sqrt{-\omega_i^2}t} - 1)}{\sqrt{-\omega_i^2}} && \text{(S49)}\\
\int_{-\infty}^{\infty} \frac{e^{i\omega t} - 1}{\omega^2} d\omega &= -\pi t && \text{(S50)}
\end{aligned}$$

for $t > 0$ with the condition $\mathrm{Re}(\omega_i^2) < 0 \vee \mathrm{Im}(\omega_i^2) \neq 0 \wedge \mathrm{Im}(\sqrt{\omega_i^2}) \neq 0$, we can rewrite the integral of the MSD eq. (S38) as

$$C_{\mathrm{MSD}}(t) = \frac{B}{\tau^4}\left(\frac{k_1 t}{\omega_1^2\omega_2^2\omega_3^2} + \sum_{i=1}^3 \frac{e^{-\sqrt{-\omega_i^2}t} - 1}{\sqrt{-\omega_i^2}\prod_{j\neq i}(\omega_i^2 - \omega_j^2)}\left[\frac{k_1}{\omega_i^2} + k_2 + k_3\omega_i^2\right]\right)\,. \tag{S51}$$

The VACF can be computed from the MSD by using eq. (16) as

$$C_{vv}(t) = \frac{B}{2\tau^4}\left(\sum_{i=1}^3 \frac{\sqrt{-\omega_i^2}e^{-\sqrt{-\omega_i^2}t}}{\prod_{j\neq i}(\omega_i^2 - \omega_j^2)}\left[\frac{k_1}{\omega_i^2} + k_2 + k_3\omega_i^2\right]\right)\,. \tag{S52}$$

The calculation of the MSD for the friction kernel of eq. (19) proceeds in the same way as shown for the kernel eq. (29). The response function then takes the slightly different form

$$\tilde{\chi}(\omega) = \left(-\omega^2 + i\omega a + i\omega b\left(\frac{i\omega + \frac{2}{\tau}}{\Omega^2 + (i\omega + \frac{1}{\tau})^2}\right)\right)^{-1}\,. \tag{S53}$$

The integral eq. (S35) resulting in the MSD can be written in the same form as before shown in eq. (S38), where the constants c_1, c_2, k_1, k_2 take different values of

$$\begin{aligned}
c_1 &= (a + 2b\tau + a\Omega^2\tau^2)^2 && \text{(S54)}\\
c_2 &= (1 + 2(a^2 - 3b + \Omega^2)\tau^2 + (-2a^2\Omega^2 + (b + \Omega^2)^2)\tau^4) && \text{(S55)}\\
k_1 &= -2(1 + \Omega^2\tau^2)(a + 2b\tau + a\Omega^2\tau^2) && \text{(S56)}\\
k_2 &= 4a\tau^2(\Omega^2\tau^2 - 1)\,. && \text{(S57)}
\end{aligned}$$

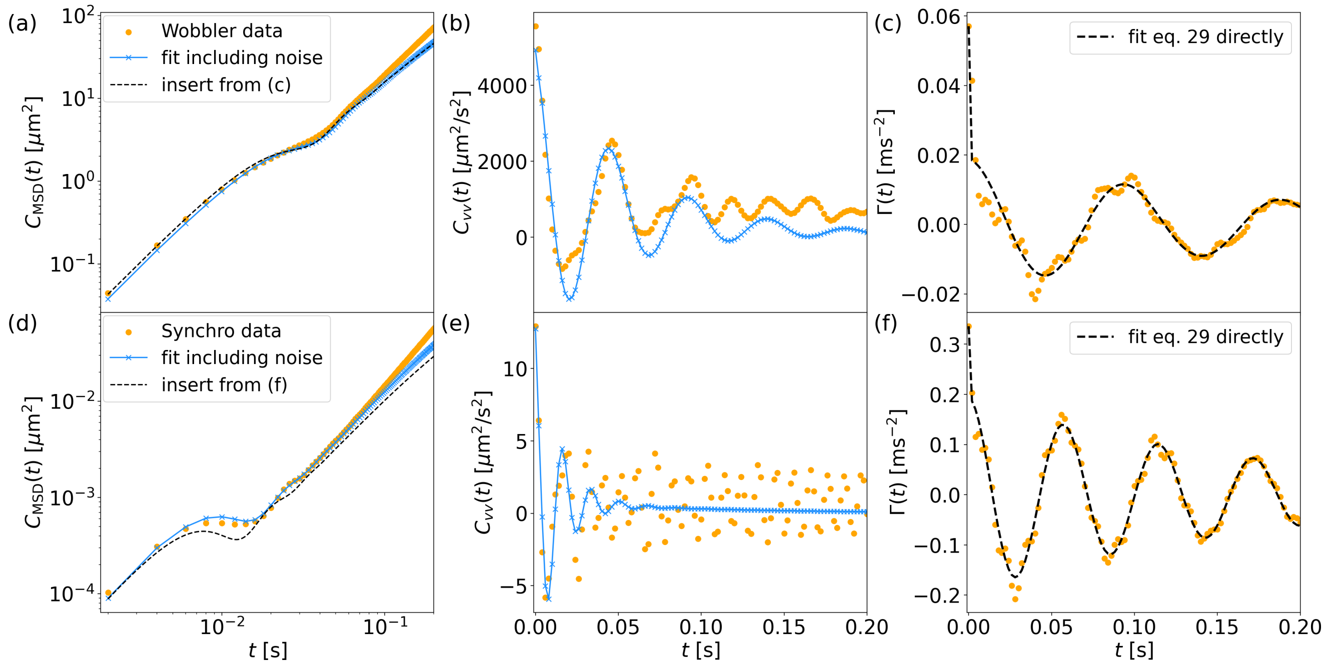

Figure S3: Two example cells for which the localization-noise fit to the VACF does not agree well with the data. The MSD $C_{\mathrm{MSD}}(t)$ (eq. (28)), VACF $C_{vv}(t)$ (eq. (25)) and friction kernel $\Gamma(t)$ extracted by eq. (8) of a single (a)-(c) wobbler and (d)-(f) synchro are displayed as orange dots. Blue crosses in the VACF figures represent the results from the localization noise fit according to eq. (17) and the blue crosses in the MSD figures result from inserting the fit parameters of the VACF into eq. (15). The black dashed line in (c) and (f) is the direct fit of eq. (29) to the extracted kernels, which is used to obtain parameters for the cell classification, see Methods Sec. 2.6. The dashed black lines in the MSD figures result from inserting the fitting result of the friction kernel (dashed lines in (c), (f) respectively) and the mean squared velocity $B = C_{vv}(0)$ into the analytical solution of the MSD eq. (S51).

Thus, the MSD of the GLE eq. (23) with the friction kernel eq. (19) can be described by the same form eq. (S51) as for the friction kernel of eq. (29), where only the constants c_i and k_i have slightly different values as seen by comparing eqs. (S39)-(S44) to eqs. (S54)-(S57).

VII Localization noise fit

The fitting procedure including a finite time step and localization noise described in Sec. 2.7 of the main text does sometimes not converge to results that agree well with the VACF of the data. In Fig. S3 we show two examples for which the fit including the localization noise (Methods eq. (17)) does not agree well with the VACF data. For 14 out of the 59 cells, the fits of the VACF using eq. (18) do not converge to a stable set of parameters. Still the direct fit of the friction kernel model eq. (29) agrees perfectly with the extracted friction kernels, which is why we use this direct fit to extract parameters, that are later used in the cluster analysis.

A typical size of the localization noise width resulting from the fits is $\sigma_{\mathrm{loc}} \approx 0.02\,\mu\mathrm{m}$. Considering the resolution of the microscope of roughly $\sim 0.5\,\mu\mathrm{m}$ and approximating the area of a cell as roughly $25\pi\,\mu\mathrm{m}^2$, one can estimate the number of pixels per cell as 100π. Thus, the error of the mean position of a cell evaluated by all pixels is estimated by $0.5\,\mu\mathrm{m}/\sqrt{100\pi} \approx 0.03\,\mu\mathrm{m}$, which is very close to the fitted localization noise width of $\sigma_{\mathrm{loc}} \approx 0.02\,\mu\mathrm{m}$. For a typical velocity around $v = 100\,\mu\mathrm{m/s}$, the displacement during one time step $\Delta = 0.002\,\mathrm{s}$ is $0.2\,\mu\mathrm{m}$. Therefore, the localization noise accounts for $\sim 10\%$ of the cell displacement. This relatively small localization noise explains why the direct fit of the model eq. (29) to the extracted friction kernels works so well and the inclusion of localization noise effects is not necessary for our data set.

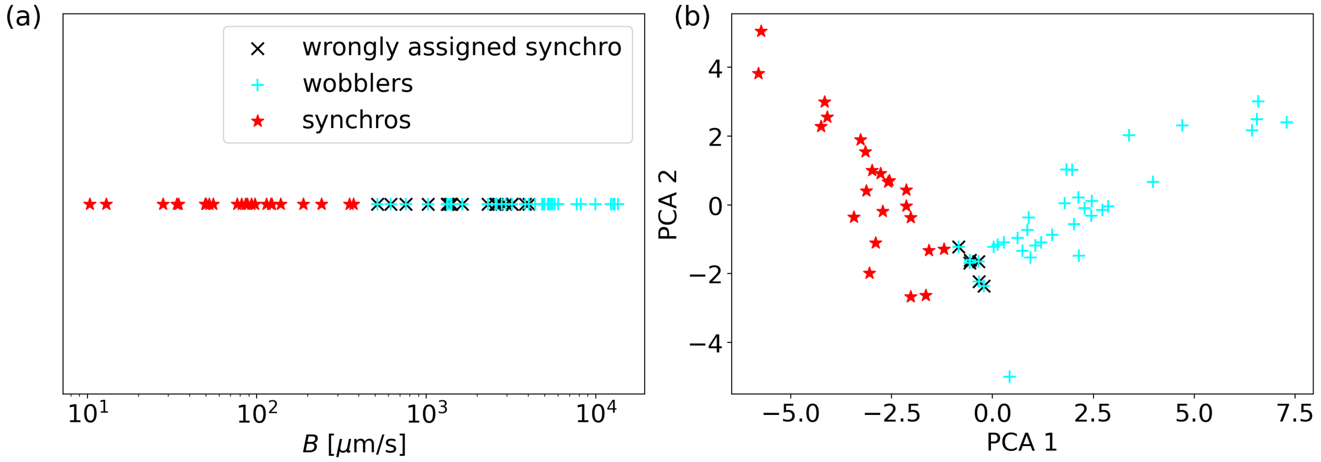

Figure S4: (a) Mean squared velocities B for all cells; a cluster analysis solely based on B leads to the wrong assignment of 18 wobblers as synchros, indicated by black crosses. (b) Projection of the five dimensional extracted parameters shown in Fig. 5 on the first two components of the PCA, which explain $61\,\%$ and $22\,\%$ of the total parameter variance, respectively. The black crosses indicate six wobbler cells that are wrongly assigned as synchros when applying the cluster analysis only to the shown two PCA components.

VIII Cluster analysis in lower dimensions

Since the wobblers are found to exhibit higher average speeds than the synchros, an intuitive and simple approach would be to distinguish the cells solely by their mean squared velocity B. Applying the cluster analysis described in Sec. 2.8 of the Methods in one dimension for B, results in a assignment agreeing only to $69\,\%$ with the full classification result, see Fig. S4a. Especially slow wobblers are wrongly assigned as synchros, as indicated in Fig. S4a by the black crosses.

As discussed in the main text, high-dimensional data sets are often analyzed with principal-component analysis (PCA), which determines the directions explaining most of the data variance. Applying a PCA to the five-dimensional parameter space of the extracted friction kernel parameters eq. (29) and B shown in Fig. 5, we find the first two components of the PCA to explain $83\,\%$ of the total parameter variance. This explanation of variance is not to be confused with the agreement of the clustering method with the assignment by visual inspection of the flagella. Here, we use the PCA tool implemented in *sklearn* for python. The normalized vectors of the shown two PCA components in the order $(a,\ b,\ \tau,\ \Omega,\ B)$ are given by $(-0.49,\ -0.29,\ 0.16,\ -0.01,\ 0.81)$ and $(0.63,\ 0.41,\ -0.5,\ 0.05,\ 0.43)$, respectively. This indicates, that no parameter alone can describe the complete variance and therefore no parameter alone can explain all the differences between synchros and wobblers. The parameters most important for the variance and discriminability of the single CR cells are a, b and B. However, the distinction into the two groups of wobblers and synchros by our cluster analysis (Sec. 2.8 in the Methods) works the best using the complete five dimensional set of parameters. Applying the cluster analysis to the first two PCA components results in an accuracy of $90\,\%$, as shown in Fig. S4b. Six wobblers are assigned to belong to the cluster of synchros (defined by the classification of (51) that agrees with our cluster analysis of the complete parameter set Fig. 5) as they lie in the transition area between the two clusters and are indicated by the black crosses in Fig. S4b.

IX Comparing friction kernel expressions eq. (29) and eq. (19)

The friction kernel of eq. (29) in the main text used to extract parameters from the data is very similar to the friction kernel eq. (19), which is derived in Sec. XIII from a system of harmonically coupled particles. The term $1/(\tau\Omega)$ in front of the sine in eq. (19) becomes small when the decay time τ is longer than the oscillation period $1/\Omega$. Several oscillations occur before the friction kernel has decayed for the CR data in Fig. 3 in the main text and Fig. S6, thus, the term $1/(\tau\Omega)$ is indeed small and the two friction kernel models have very similar shapes, as we show in Fig. S6. Here, we insert the extracted parameters from the friction kernel eq. (29) of two example cells into eq. (19), the deviations are seen to be very small.

10

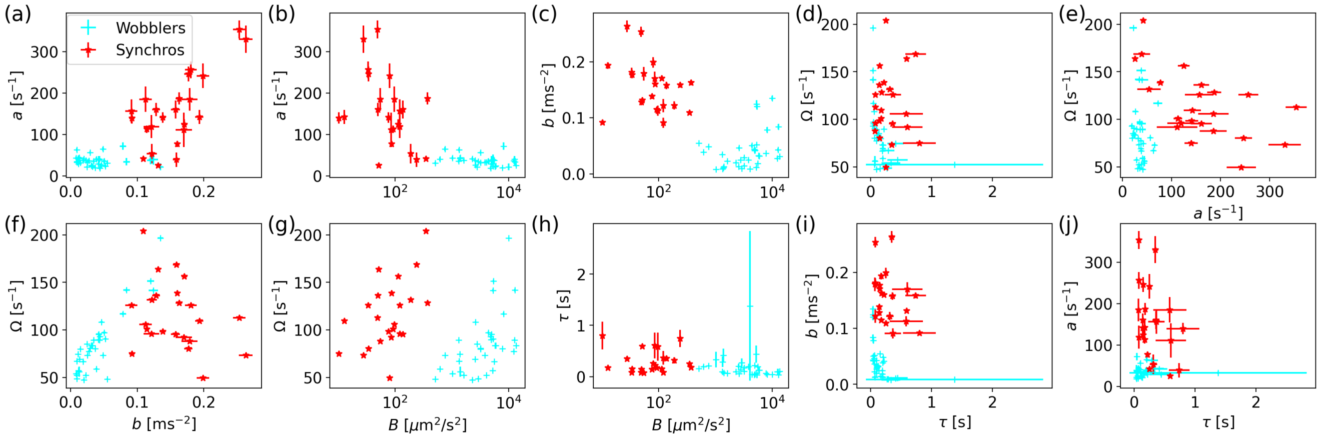

Figure S5: Same figure as in the main text Fig. 5 but with error estimates for the parameters, which we obtain from scipy's curve fit function as the square root of the diagonal elements of the approximate covariance around the optimal fit result. The errors of all parameters are in the range of a few percent, except for the decay time τ, where the highest error is in the same order as the parameter value itself.

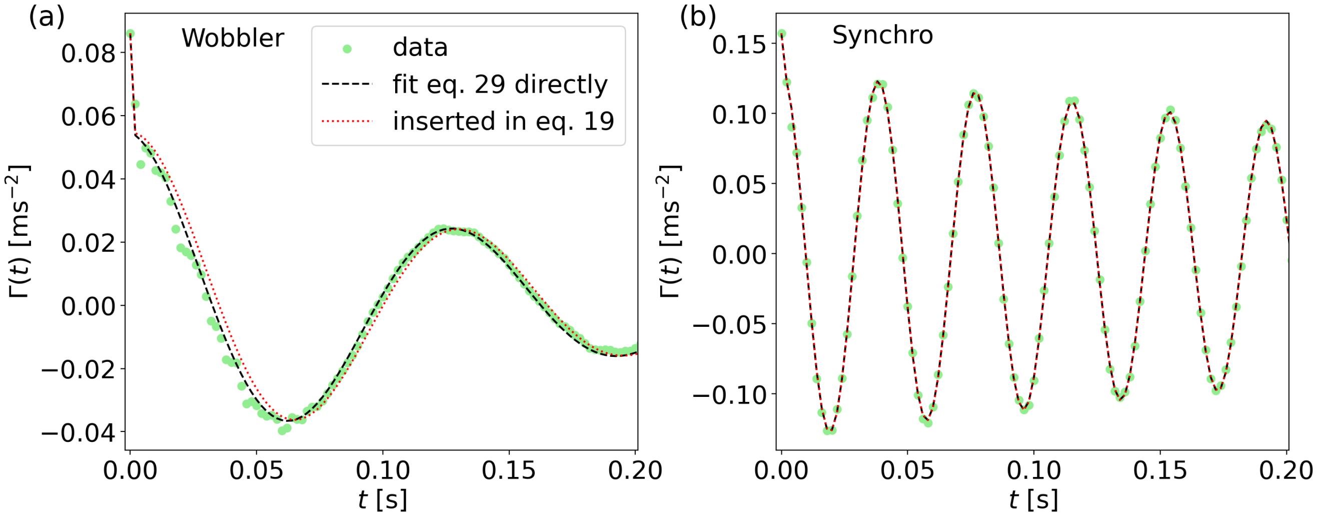

Figure S6: Friction kernel $\Gamma(t)$ extracted by eq. (8) of (a) a single wobbler and (b) single synchro shown as green data points. The black line shows the direct fit of eq. (29) to the data points and the red dotted line results from inserting the fitted parameters from the black dashed line into eq. (19) derived from the Hamiltonian eq. (20) in Sec. XIII. The agreement of the black dashed line and the red dotted line means that in the parameter range exhibited by the CR cells, the two kernels eq. (29) and eq. (19) describe the data equally well.

X Exemplary non-equilibrium model describing CR motion

The effective friction kernel $\Gamma(t)$ given by eq. (19) leads to the Fourier transform

$$\begin{aligned}\tilde{\Gamma}^+(\omega) &= \int_0^\infty \left(2a\delta(t) + be^{-t/\tau}\left(\cos(\Omega t) + \frac{1}{\tau\Omega}\sin(\Omega t)\right)\right) e^{-i\omega t} dt \\ &= a + b\left(\frac{i\omega + \frac{2}{\tau}}{\Omega^2 + (i\omega + \frac{1}{\tau})^2}\right) . \end{aligned} \tag{S58}$$

Inserting eq. (S58) into eq. (4) yields the Fourier-transformed VACF

$$\begin{aligned}\frac{\tilde{C}_{vv}(\omega)}{B} &= \Big(2a + 2b\tau + 4a\Omega^2\tau^2 + 2b\Omega^2\tau^3 + 2a\Omega^4\tau^4 + 4ib\tau^2\omega \\ &+ (4a\tau^2 + 2b\tau^3 - 4a\Omega^2\tau^4)\omega^2 + 2a\tau^4\omega^4\Big)/ \\ &\Big(a + 2b\tau + a\Omega^2\tau^2)^2 + (1 + 2(a^2 - 3b + \Omega^2)\tau^2 + (-2a^2\Omega^2 + (b + \Omega^2)^2)\tau^4)\omega^2 \\ &+ \tau^2(2 + (a^2 - 2(b + \Omega^2))\tau^2)\omega^4 + \tau^4\omega^6\Big).\end{aligned} \tag{S59}$$

In order to find a non-equilibrium model, that leads to the same VACF as the kernel in eq. (19), we assume a specific form of the velocity friction kernel

$$\Gamma_v(t) = 2a_v\delta(t) \tag{S60}$$

which leads to the Fourier transform $\tilde{\Gamma}_v^+(\omega) = a_v$. We insert this together with the result from eq. (S59) into eq. (5) and solve for $\tilde{\Gamma}_R(\omega)$. By Fourier back transformation the result can be written in the form

$$\Gamma_R(t) = \int_{-\infty}^{\infty} \frac{d\omega}{2\pi} e^{i\omega t} \frac{k_0 + k_1\omega + k_2\omega^2 + k_3\omega^3 + k_4\omega^4 + k_6\omega^6}{c_0 + c_1\omega^2 + c_2\omega^4 + c_3\omega^6} \tag{S61}$$

with the constants c_i and k_i being

$$\begin{aligned} c_0 &= (a + 2b\tau + a\Omega^2\tau^2)^2 \\ c_1 &= 1 + 2(a^2 - 3b + \Omega^2)\tau^2 + (-2a^2\Omega^2 + (b + \Omega^2)^2)\tau^4 \\ c_2 &= \tau^2(2 + (a^2 - 2(b + \Omega^2))\tau^2) \\ c_3 &= \tau^4 \\ k_0 &= 2aa_v^2 + 2a_v^2 b\tau + 4aa_v^2\Omega^2\tau^2 + 2a_v^2 b\Omega^2\tau^3 + 2aa_v^2\Omega^4\tau^4 \\ k_1 &= 4a_v^2 b\tau^2 \\ k_2 &= 2(b\tau(1 + a_v^2\tau^2 + \Omega^2\tau^2) + a((1 + \Omega^2\tau^2)^2 + a_v^2(2\tau^2 - 2\Omega^2\tau^4))) \\ k_3 &= 4b\tau^2 \\ k_4 &= 2\tau^2(b\tau + a(2 + a_v^2\tau^2 - 2\Omega^2\tau^2)) \\ k_6 &= 2a\tau^4\,. \end{aligned}$$

We solve this Fourier transform by performing the same steps as for the calculation of the integral (S38) derived in Sec. VI, where ω_i^2 are the roots of the denominator of eq. (S61) in ω^2, which we obtain numerically, and we additionally use

$$\int_{-\infty}^{\infty} \frac{\omega e^{i\omega t}}{\omega^2 - \omega_i^2} d\omega = i\pi e^{-\sqrt{-\omega_i^2}t}\,. \tag{S62}$$

The friction kernel in time domain is then retrieved as

$$\begin{aligned}\Gamma_R(t) = 2a\delta(t) + \frac{1}{2c_3}\sum_{i=1}^{3}\Bigg(&\frac{e^{-\sqrt{-\omega_i^2}t}}{\sqrt{-\omega_i^2}\prod_{j\neq i}(\omega_i^2 - \omega_j^2)}\left[k_0 + k_2\omega_i^2 + k_4(\omega_i^2)^2 + k_6(\omega_i^2)^3\right] \\ &- \frac{e^{-\sqrt{-\omega_i^2}t}}{\prod_{j\neq i}(\omega_i^2 - \omega_j^2)}\left[k_1 + k_3\omega_i^2\right]\Bigg).\end{aligned} \tag{S63}$$

This exemplary mapping shows, that a simple Markovian friction kernel given by eq. (S60) combined with a random force correlation function that contains additional oscillating components,given by eq. (S63), lead to the same correlation function as described by the GLE with the effective friction kernel given by eq. (19). We have thus derived one possible non-equilibrium model that describes CR cell motion.

Furthermore, this non-equilibrium model with $\Gamma_R(t)$ given by eq. (S63) and $\Gamma_v(t)$ given by eq. (S60) corresponds to the coupled system of differential equations

$$\dot{x}(t) = v(t) \tag{S64}$$

$$\dot{v}(t) = -a_v v(t) + \xi_x(t) + \sum_{i=1}^{3} F_i(t) \tag{S65}$$

$$\dot{F}_i(t) = -\frac{1}{\tau_i}\left(F_i(t) - \xi_i^R(t)\right) , \tag{S66}$$

with $\langle \xi_x(0)\xi_x(t)\rangle = 2aB\delta(t)$ and $\langle \xi_i^R(0)\xi_j^R(t)\rangle = 2a_i^R B\delta_{ij}\delta(t)$.
Solving eq. (S66) for $F_i(t)$ leads to

$$F_i(t) = -\frac{1}{\tau_i}\int_0^t e^{-(t-t')/\tau_i}\xi_i^R(t')dt' , \tag{S67}$$

which yields $\langle F_i(0)F_i(t)\rangle = B\frac{a_i^R}{\tau_i}e^{-t/\tau_i}$. Now defining the random force as $F_R(t) = \xi_x(t) + \sum_{i=1}^3 F_i(t)$, we can write eq. (S65) as the GLE eq. (23) with

$$\Gamma_R(t) = 2a\delta(t) + \sum_{i=1}^{3}\frac{a_i^R}{\tau_i}e^{-t/\tau_i} \tag{S68}$$

and $\Gamma_v(t) = 2a_v\delta(t)$. Thus, the system of eqs. (S64)-(S66) is equivalent to the GLE eq. (23) with $\Gamma_R(t)$ given by eq. (S63) and $\Gamma_v(t)$ given by eq. (S60), when the parameters are given by

$$\tau_i = \frac{1}{\sqrt{-\omega_i^2}} \tag{S69}$$

$$a_i^R = \frac{1}{-2c_3\omega_i^2\prod_{j\neq i}(\omega_i^2 - \omega_j^2)}\left[k_0 + k_2\omega_i^2 + k_4(\omega_i^2)^2 + k_6(\omega_i^2)^3 - \sqrt{-\omega_i^2}(k_1 + k_3\omega_i^2)\right] . \tag{S70}$$

XI Information on the cell orientation is contained in the cell-center trajectories

The mean direction of motion, exponentially weighted over the past trajectory, is defined by

$$\vec{V}(t) = \frac{1}{T}\int_{-\infty}^{t} e^{-(t-t')/T}\vec{v}(t')dt' \quad , \tag{S71}$$

where T is the exponential decay time and $\vec{v}(t) = (v_x(t), v_y(t))$ is the two-dimensional velocity (31). This running-average velocity vector $\vec{V}(t)$ points in the direction in which a cell has moved in the past, the running-average position follows as

$$\vec{R}(t) = \int_0^t \vec{V}(t')dt' . \tag{S72}$$

In Fig. S7a we show the trajectory of a synchro and its running average position $\vec{R}(t)$ for $T = 0.1$ s, $\vec{R}(t)$ resolves the mean direction of motion but not the back-and-forth motion on time scales below T. From the circular path of the trajectory it follows that the mean direction of motion changes, this is reflected in Fig. S7b by the change of the angle of $\vec{R}(t)$ with the x-axis (red data).

The orientation of cells is determined from the videos by fitting an ellipsoid to the cell perimeter and given by the main axis of the ellipsoid. Since CR cells move with their flagella in front, the orientation vector is defined to point towards the side where the flagella are anchored.

The cell orientation vector coincides very well with the direction of the running average velocity $\vec{V}(t)$, as shown in Fig. S7b. Therefore, the information on the orientation of the CR cells is included in the cell trajectory $(v_x(t), v_y(t))$ via eq. (S71). Our description of the CR motion by the GLE eq. (23) captures the correct behavior of the cell position and velocity over time, which is demonstrated by Figs. 4a-f. Since the cell orientational information is included in the trajectory, we conclude that the GLE model also correctly describes the CR orientational dynamics.

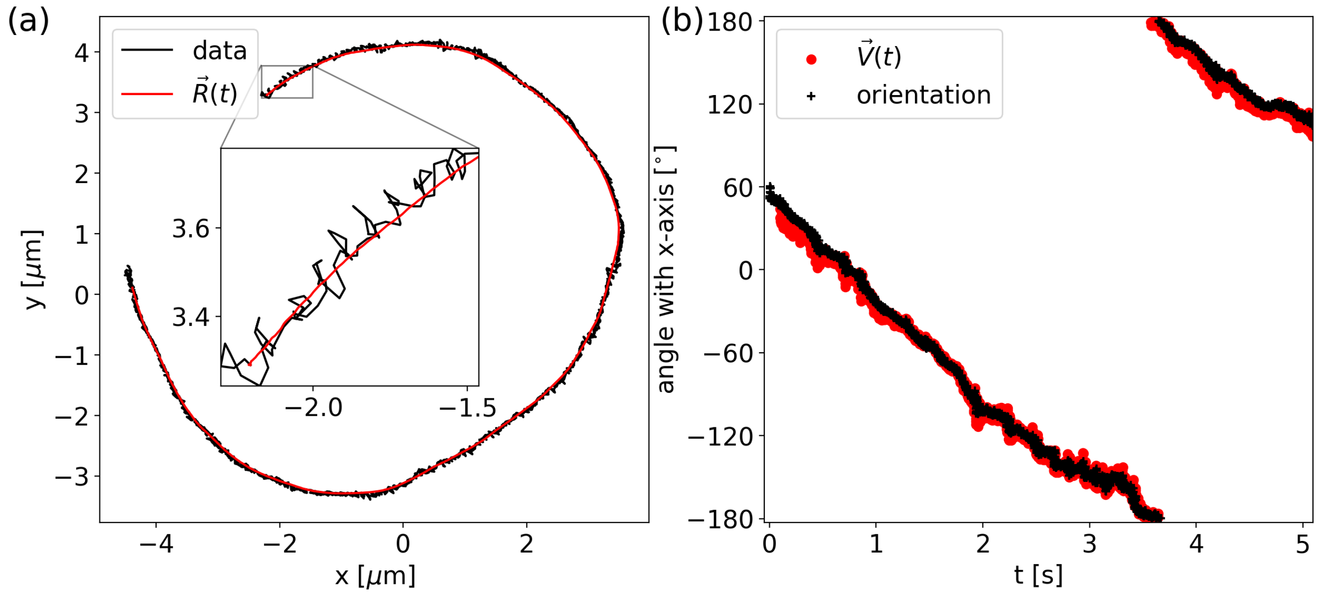

Figure S7: (a) Trajectory of a synchro shown in black, the running average position defined by eqs. (S71), (S72) with a decay time $T = 0.1\,\mathrm{s}$ is shown in red. The inset in the middle shows a zoom into the first $0.2\,\mathrm{s}$ of the trajectory. (b) The angle between the x-axis and the cell orientation directly extracted from video data is shown for the trajectory in (a) as black crosses. The angle between the x-axis and the running-average velocity $\vec{V}(t)$ determined by eq. (S71) is shown in red.

XII Effects of smoothing trajectory data

Since the localization noise modifies the VACF and thereby the friction kernel that is extracted from the data, it is important to smooth the data at a level that reduces the noise without loosing detail. For every data set, there is an optimal level of smoothing.

A natural way of smoothing data is by sequentially averaging over neighboring data points, which conserves the discretization time step. Here, we use an iterative averaging of the position data over two consecutive points according to

$$x_i^{n+1} = \frac{x_i^n + x_{i+1}^n}{2}\,, \tag{S73}$$

with n being the number of smoothing iterations. We show the effect of the smoothing level on the VACF and the localization noise fit in Fig. S8 and the effect on the friction kernel in Fig. S9. For high smoothing iterations, the cell speed is underestimated, which is reflected by a decreasing mean squared velocity B for increasing n in Fig. S8. At the same time, the fitted localization noise strength σ_{loc} decreases with increasing n. This is reflected by the smaller difference between the first two data points of the VACF.

The estimate of the localization noise strength for high smoothing iterations n is not reliable anymore because it becomes very small, this explains why it is not decreasing monotonically with growing n, see Fig. S8. Since the first smoothing iteration $n = 1$ simply adds two consecutive points according to eq. (S73), one in principle expects the localization noise width σ_{loc} to be halved compared to the non-smoothed data. This indeed one can see for the synchro shown in Fig. S8.

Since the smoothing of the data decreases the amplitude of the VACF, as seen in Fig. S8, it consequently decreases the amplitudes of the friction kernel parameters a and b and therefore at the same time it reduces the kernel frequency Ω, as Ω exhibits a complex relation with the frequency of the VACF, which is explained in detail in Sec. V. Moreover, we find the dip in the friction kernel at the first time step to decrease for higher smoothing iterations shown in Fig. (S9), which indicates that the dip in the friction kernel originates from the localization noise. This is explained by eq. (8), since the first point of the VACF is overestimated and the second point of the VACF is underestimated due to the localization noise, which then propagates into the friction kernel and leads to a dip at short times.

The VACF of the smoothed data is less influenced by the localization noise, nevertheless, with every smoothing iteration, the deviation of the smoothed VACF from the non-smoothed VACF becomes larger, as shown in Fig. S8. Thus, we

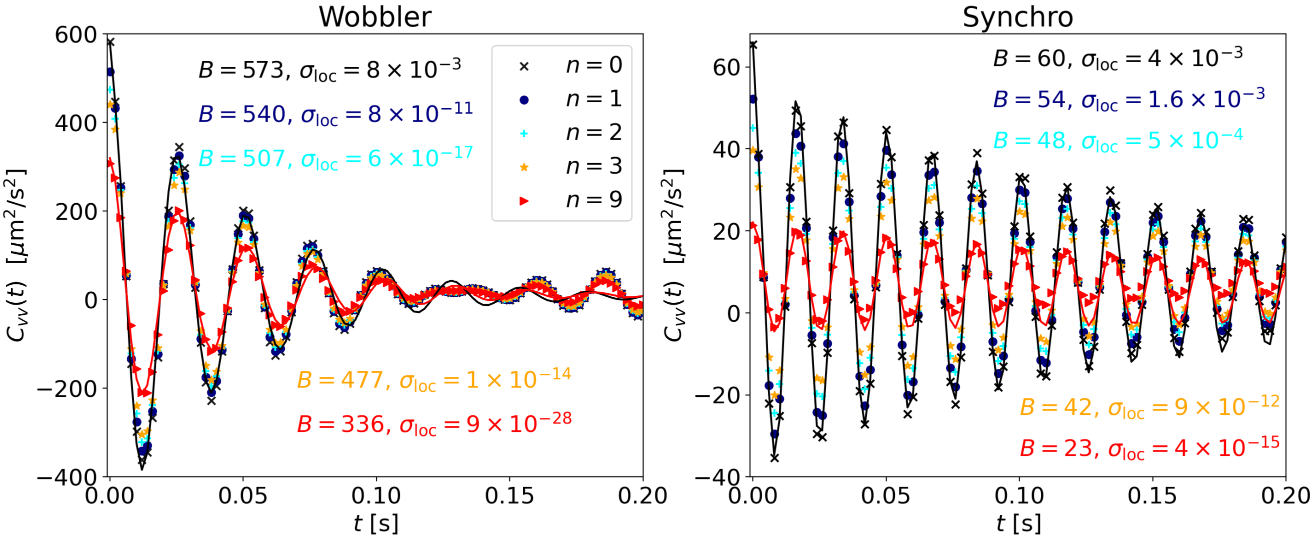

Figure S8: The VACF $C_{vv}(t)$ of a wobbler (left) and a synchro (right) is shown for different smoothing iterations n described by eq. (S73), where $n = 0$ denotes the original non-smoothed data. The mean-squared velocities B in units of $\mu\mathrm{m}^2/\mathrm{s}^2$ and the fitted localization noise width σ_{loc} in units of $\mu\mathrm{m}$ are both given in the color of the according smoothing iteration. The fitting result of the VACF including localization noise, described in Sec. VII, is shown as solid lines for $n = 0$ and $n = 9$ in the respective color.

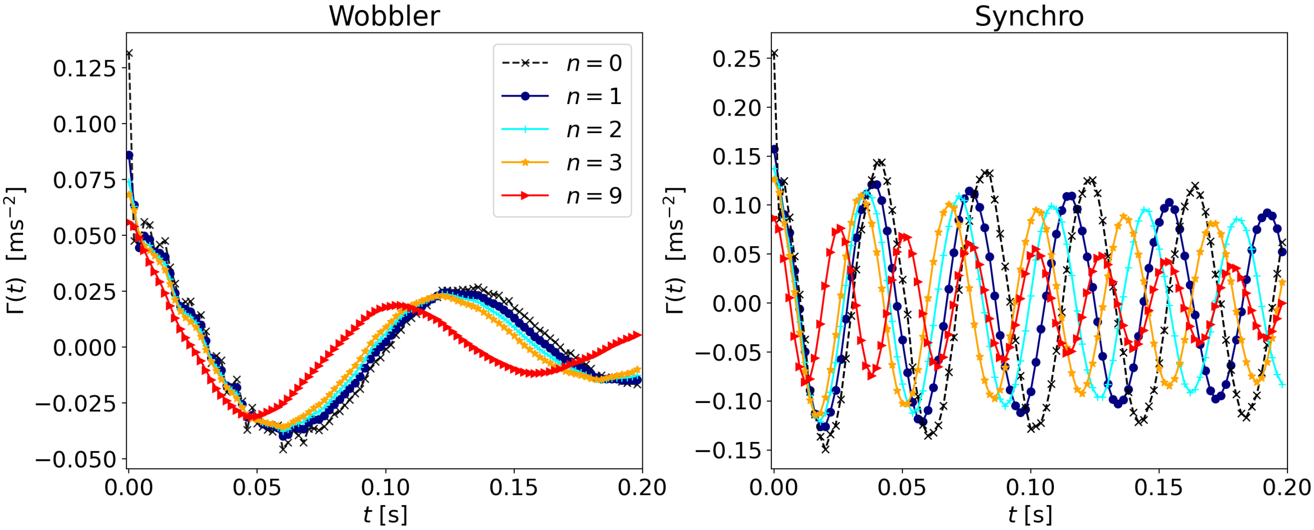

Figure S9: The friction kernel $\Gamma(t)$ extracted according to eq. (8) from data at different smoothing iterations described by eq. (S73) is given for a wobbler on the left and for a synchro on the right. The solid lines connecting the data points are guides to the eye.

choose $n = 1$ smoothing iterations for our data as a compromise between minimizing the effect of noise and keeping a good resolution that accurately describes the actual cell velocities.

XIII Markovian embedding of the friction kernel eq. (19)

Here we derive the equivalency of the system of coupled equations of motion

$$
\begin{aligned}
\dot{x}(t) &= v(t) && \text{(S74)}\\
m\dot{v}(t) &= -\gamma_x v + bm(y(t) - x(t)) + F_{Rx}(t) && \text{(S75)}\\
\dot{y}(t) &= v_y(t) && \text{(S76)}\\
m_y \dot{v}_y(t) &= -\gamma_y v_y + bm(x(t) - y(t)) + F_{Ry}(t)\,, && \text{(S77)}
\end{aligned}
$$

describing two harmonically coupled massive reaction coordinates in a heat bath based on the Hamiltonian eq. (20), to the GLE eq. (23) with the effective friction kernel of eq. (19) (61). Here, the random forces have zero mean $\langle F_i(t)\rangle = 0$ and are delta correlated $\langle F_i(0)F_j(t)\rangle = 2\gamma_i k_B T \delta_{ij}\delta(t)$. We start by finding the solution for $\vec{z}(t) = (y, \dot{y})^T$ to the subsystem eqs. (S76) and (S77), which is the solution to

$$
\dot{\vec{z}}(t) = A\vec{z}(t) + \begin{pmatrix} 0 \\ F_{Ry}(t)/m_y + \frac{bm}{m_y}x(t) \end{pmatrix} \tag{S78}
$$

with

$$
A = \begin{pmatrix} 0 & 1 \\ -\frac{bm}{m_y} & -\frac{\gamma_y}{m_y} \end{pmatrix} \tag{S79}
$$

and consequently

$$
A^{-1} = \begin{pmatrix} -\frac{\gamma_y}{bm} & -\frac{m_y}{bm} \\ 1 & 0 \end{pmatrix}\,. \tag{S80}
$$

One finds the solution to eq. (S78) as

$$
\vec{z}(t) = e^{A[t-t_0]}\vec{z}(t_0) + \int_{t_0}^{t} dt' e^{A[t-t']} \begin{pmatrix} 0 \\ F_{Ry}(t')/m_y + \frac{bm}{m_y}x(t') \end{pmatrix}\,. \tag{S81}
$$

Performing a partial integration on the integral in eq. (S81) for the term including $x(t')$ leads to

$$
\begin{aligned}
\vec{z}(t) = e^{A[t-t_0]}\vec{z}(t_0) &+ \int_{t_0}^{t} dt' A^{-1} e^{A[t-t']} \begin{pmatrix} 0 \\ \frac{bm}{m_y} v(t') \end{pmatrix} \\
&- \left[A^{-1} e^{A[t-t']} \begin{pmatrix} 0 \\ \frac{bm}{m_y} x(t') \end{pmatrix} \right]_{t_0}^{t}, \\
&+ \int_{t_0}^{t} dt' e^{A[t-t']} \begin{pmatrix} 0 \\ F_{Ry}(t')/m_y \end{pmatrix},
\end{aligned} \tag{S82}
$$

where the matrix exponential e^{At} can be expressed in terms of the Eigenvalues of A

$$
\lambda_{1,2} = -\frac{\gamma_y}{2m_y} \pm \omega_0 \tag{S83}
$$

with

$$
\omega_0 = \sqrt{\left(\frac{\gamma_y}{2m_y}\right)^2 - \frac{bm}{m_y}} \tag{S84}
$$

as

$$
\begin{aligned}
e^{At} &= \frac{1}{\lambda_2 - \lambda_1} \begin{pmatrix} \lambda_2 e^{\lambda_1 t} - \lambda_1 e^{\lambda_2 t} & e^{\lambda_2 t} - e^{\lambda_1 t} \\ \lambda_1\lambda_2(e^{\lambda_1 t} - e^{\lambda_2 t}) & \lambda_2 e^{\lambda_2 t} - \lambda_1 e^{\lambda_1 t} \end{pmatrix} \\
&= e^{-t\frac{\gamma_y}{2m_y}} \begin{pmatrix} \cosh(\omega_0 t) + \frac{\sinh(\omega_0 t)\gamma_y}{2m_y\omega_0} & \sinh(\omega_0 t)/\omega_0 \\ -\frac{\sinh(\omega_0 t) bm}{\omega_0 m_y} & \cosh(\omega_0 t) - \frac{\sinh(\omega_0 t)\gamma_y}{2m_y\omega_0} \end{pmatrix}
\end{aligned} \tag{S85}
$$

consequently leading to

$$
A^{-1}e^{At} = e^{-t\frac{\gamma_y}{2m_y}} \begin{pmatrix} -\frac{\cosh(\omega_0 t)\gamma_y}{bm} + \frac{\sinh(\omega_0 t)}{\omega_0}\left(1 - \frac{\gamma_y^2}{2bmm_y}\right) & -\frac{\sinh(\omega_0 t)\gamma_y}{2bm\omega_0} - \frac{\cosh(\omega_0 t)m_y}{bm} \\ \cosh(\omega_0 t) + \frac{\sinh(\omega_0 t)\gamma_y}{2m_y\omega_0} & \frac{\sinh(\omega_0 t)}{\omega_0} \end{pmatrix}\,. \tag{S86}
$$

16

The solution for $y(t)$ can now be found as the first element of the vector $\vec{z}(t)$ from eqs. (S82)-(S86) when assuming a stationary system, i.e. $t_0 \to -\infty$ as

$$\begin{aligned} y(t) = x(t) - \int_{-\infty}^{t} dt' v(t') e^{-\frac{(t-t')\gamma_y}{2m_y}} \left(\cosh(\omega_0(t-t')) + \frac{\gamma_y}{2m_y\omega_0} \sinh(\omega_0(t-t')) \right) \\ + \int_{-\infty}^{t} dt' F_{Ry}(t') e^{-\frac{(t-t')\gamma_y}{2m_y}} \frac{\sinh(\omega_0(t-t'))}{m_y\omega_0}. \end{aligned} \tag{S87}$$

Inserting eq. (S87) into eq. (S75), one recovers the GLE eq. (23) for $x(t)$

$$m\ddot{x}(t) = -\int_{-\infty}^{t} dt' \bar{\Gamma}(t-t') + \bar{F}_R(t), \tag{S88}$$

where the bars indicate multiplication by the mass $\Gamma(t) = \bar{\Gamma}(t)/m$, $F_R = \bar{F}_R/m$ and the friction kernel takes the oscillatory form

$$\Gamma(t) = \frac{\gamma_x}{m}\delta(t) + be^{-t\frac{\gamma_y}{2m_y}} \left(\cosh(\omega_0 t) + \frac{\gamma_y}{2m_y\omega_0} \sinh(\omega_0 t) \right) \tag{S89}$$

and the random force is explicitly given by

$$F_R(t) = \bar{F}_{Rx}/m + b\int_{-\infty}^{t} dt' \bar{F}_{Ry}(t') e^{-\frac{(t-t')\gamma_y}{2m_y}} \frac{\sinh(\omega_0(t-t'))}{m_y\omega_0}\,. \tag{S90}$$

Computing the random force correlation, one finds

$$\langle F_R(0) F_R(t) \rangle = B\Gamma(t)\,. \tag{S91}$$

The friction kernel of eq. (19) in the Methods is equivalent to the friction kernel of eq. (S89) with the parameters given by

$$\begin{aligned} a &= \frac{\gamma_x}{m} \\ \tau &= \frac{2m_y}{\gamma_y} \\ \Omega &= i\omega_0\,. \end{aligned}$$
