# Supplementary material for: Data-driven classification of individual cells by their non-Markovian motion
Source: Biophys J. 2024 Mar 21;123(10):1173–83. doi: 10.1016/j.bpj.2024.03.023 (PMC11140416; doi:10.1016/j.bpj.2024.03.023)
Supplement: Document S2. Article plus Supporting Material [file mmc4.pdf]

Biophysical Journal
Article

Biophysical Society

Data-driven classification of individual cells by their non-Markovian motion

Anton Klimek,[1] Debasmita Mondal,[3,5] Stephan Block,[2] Prerna Sharma,[3,4] and Roland R. Netz[1,*]
[1]Fachbereich Physik, Freie Universität Berlin, Berlin, Germany; [2]Institut für Chemie und Biochemie, Freie Universität Berlin, Berlin, Germany; [3]Department of Physics, Indian Institute of Science, Bangalore, India; [4]Department of Bioengineering, Indian Institute of Science, Bangalore, India; and [5]James Franck Institute, University of Chicago, Chicago, Illinois

ABSTRACT We present a method to differentiate organisms solely by their motion based on the generalized Langevin equation (GLE) and use it to distinguish two different swimming modes of strongly confined unicellular microalgae *Chlamydomonas reinhardtii*. The GLE is a general model for active or passive motion of organisms and particles that can be derived from a time-dependent general many-body Hamiltonian and in particular includes non-Markovian effects (i.e., the trajectory memory of its past). We extract all GLE parameters from individual cell trajectories and perform an unbiased cluster analysis to group them into different classes. For the specific cell population employed in the experiments, the GLE-based assignment into the two different swimming modes works perfectly, as checked by control experiments. The classification and sorting of single cells and organisms is important in different areas; our method, which is based on motion trajectories, offers wide-ranging applications in biology and medicine.

SIGNIFICANCE Classification of cells is a common task in biology and medicine. We introduce the framework to accomplish such classification based on cell-center trajectories. Our method is based on the systematic theory for the dynamics of coarse-grained variables and extracts the underlying parameters describing active and passive cell motion. We apply our methodology to confined unicellular microalgae that exhibit two different swimming modes and show that we can accurately distinguish the two populations solely based on their motion pattern. Our method can be applied to time-series data of general observables from unicellular and multicellular organisms. We anticipate numerous applications in biology and medicine that require the label-free distinction and analysis of individual cells and organisms.

INTRODUCTION

Classifying individual cells or organisms is a challenging task that has been approached in many different ways and has ample applications. Distinguishing different types of cancer cells (1,2), foodborne pathogens (3), sperm cells (4), or types of neurons (5) are just a few examples. Different techniques have been introduced to distinguish and classify organisms on the multi-cell down to the single-cell level. One approach involves markers that bind cell specifically (2,6,7). Since individual cells contain unique genetic and epigenetic information, it is also possible to distinguish cells by their specific DNA or RNA content. Indeed, biotechnological advances enable single-cell RNA sequencing (8,9), which can be used in combination with machine-learning approaches (10,11), to efficiently distinguish single cells. However, RNA sequencing, as well as usage of markers, requires cell perturbation or even destruction for data acquisition. In many cases, it is desirable to classify cells without perturbing them, which requires label-free techniques such as spectroscopic approaches (3,12) or microscopy (13). In this way, cell information can be extracted almost instantaneously (14) from living organisms (1). Spectroscopic and microscopic images can be processed using machine learning to classify cells; however, the outcomes can be hard to interpret and require massive training data. One way to simplify the processing of cell-image data is to reduce the parameter space. This can be achieved by feature selection (15) or by projection onto important parameters, such as by principal-component analysis (11). A prime feature of mobile cells is their positional trajectory, which is relatively easy to obtain in experiments and contains hidden information on the motion-generating processes within the cell (16–20). Machine-learning algorithms have been proposed to classify

Submitted January 19, 2024, and accepted for publication March 18, 2024.
*Correspondence: rnetz@physik.fu-berlin.de
Editor: Dimitrios Vavylonis.
https://doi.org/10.1016/j.bpj.2024.03.023
© 2024 Biophysical Society.
This is an open access article under the CC BY license (http://creativecommons.org/licenses/by/4.0/).

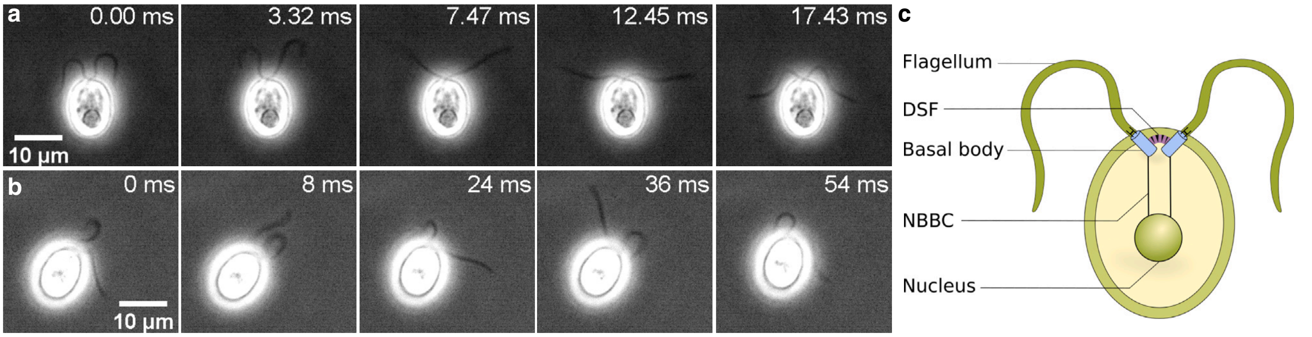

FIGURE 1 Unicellular microalgae microscopy. Sequences of phase-contrast microscopy images of CR algae exhibiting (*a*) synchro and (*b*) wobbler-type flagellar motion. The white halo around the cells is typical for phase-contrast microscopy (46). (*c*) Sketch of a CR cell: the distal striated fiber (DSF) connects the two basal bodies (47), which anchor the flagella and are connected to the nucleus by nuclear basal-body connectors (NBBCs) (45,48). To see this figure in color, go online.

trajectories (21) and some have been applied to single-cell trajectories (15). These approaches mostly focus on classification and not on the interpretation of the motion patterns. To yield a mechanistic interpretation, some specific model is usually assumed (22–27), which makes the interpretation model dependent. A general model that describes the motion of a particle in a complex environment, captures the stochasticity of its motion and can be derived from first principles is the GLE, which has been shown to accurately describe the motion of different cell types (28–32). In fact, the GLE is not an *ad hoc* model but can be derived from the underlying general many-body Hamiltonian (33–35). Living cells are intrinsically out of equilibrium (36), a fact that can be properly accounted for by the GLE used to describe the cell motion (37). In fact, there are many other models besides the GLE that have been successfully used to describe active and passive stochastic motion (38–42). The advantage of the GLE is that it makes minimal assumptions on the type of motion and encompasses many previously introduced models, such as the run-and-tumble model used to describe bacterial motion (43), as has been shown recently (44).

Here, we present a method to classify individual organisms based on the GLE parameters extracted from their motion trajectories. We apply our methods to experimental trajectories of individual unicellular biflagellate algae *Chlamydomonas reinhardtii* (CR) (45) and find two distinct groups of swimmers, which are illustrated in Fig. 1 *a* and *b*. In contrast to other existing methods for cell sorting and classification, our method requires only trajectories as input, does not need any training of a network, and avoids human bias in the selection of relevant features. Additionally, our approach allows us to interpret motion characteristics in terms of simple mechanistic models derived from the GLE parameters. In the case of CR cells, the data suggest some type of elastic coupling that presumably involves the anchoring of the flagella (45,47,48), as schematically depicted in Fig. 1 *c*, or a chemical feedback loop. Our approach is applicable to any kind of cell or organism motility data with sufficiently long trajectories and sufficiently fine temporal discretization if the coordinate describing the motion corresponds to a Gaussian process, as will be explained in detail further below.

MATERIALS AND METHODS

Cell growth and sample preparation

Wild-type CR cell cultures (strain, CC-1690) are grown in tris-acetate-phosphate (TAP + P) medium by alternating light:dark (12:12 h) cycles for 3 days. We collect the cell suspension in their actively growing phase (between third and sixth day of culture) 2–3 h after the beginning of the light cycle and re-suspend it in fresh TAP + P medium. After 30 to 40 min of equilibration to recover from the mechanosensitive shock during re-suspension (49), we inject the cells inside a rectangular quasi-2D microfluidic chamber of height 10 μm and area 18 $\times$ 6 mm. This chamber is assembled by using a glass slide and coverslip sandwiched with a 10 $-$ μm double-sided tape (Nitto Denko corporation) as spacer. The glass surfaces are pre-cleaned and coated with a polyacrylamide brush to suppress nonspecific adhesion of cell body and flagella (50). The chamber height is determined as 10.88 ± 0.68 μm across different samples (51).

Recording of trajectories

The cells in the chamber are placed under red light illumination (> 610 *nm*) to prevent phototaxis (52) and flagellar adhesion (53) of CR (51). We use high-speed video microscopy (Olympus IX83/IX73) at 500 frames per second with a 40$\times$ phase-contrast objective (Olympus, 0.65 NA, Plan N, PH2) connected to a metal oxide semiconductor (CMOS) camera (Phantom Miro C110, Vision Research, pixel size = 5.6 μm) for imaging the mid-plane between the confining glass plates. This setup enables us to simultaneously image cell position and flagellar shape. To capture very long trajectories to probe the long-time diffusive behavior in the supporting material, we use a 10$\times$ bright-field objective (Olympus, 0.25 NA, PlanC N) connected to a high-speed CMOS camera of higher pixel length (pco.1200hs, pixel size = 12 μm) at 50 frames per second. We determine cell trajectories by binarizing the image sequences with appropriate threshold parameters and tracking their centers using standard MATLAB routines (54).

Velocity autocorrelation function

Fourier transformation of Eqs. 23 and 24 leads to

$$\tilde{v}(\omega) = \frac{\tilde{F}_R(\omega)}{\tilde{\Gamma}_v^+(\omega) + i\omega} \quad \text{(Equation 1)}$$

with the single-sided Fourier transform defined as $\tilde{\Gamma}_v^+(\omega) = \int_0^\infty e^{-i\omega t}\Gamma_v(t)dt$ and

$$\langle \tilde{F}_R(\omega)\tilde{F}_R(\omega')\rangle = 2\pi B\delta(\omega+\omega')\tilde{\Gamma}_R(\omega') . \quad \text{(Equation 2)}$$

From the Fourier transform of the velocity autocorrelation function (VACF)

$$\begin{aligned}\tilde{C}_{vv}(\omega) &= \int_{-\infty}^{\infty} dte^{-i\omega t}\langle v(0)v(t)\rangle \\ &= \int_{-\infty}^{\infty} e^{-i\omega t}dt \int_{\infty}^{\infty} e^{i\omega t}\frac{d\omega}{2\pi}\int_{-\infty}^{\infty}\frac{d\omega}{2\pi}\langle \tilde{v}(\omega)\tilde{v}(\omega')\rangle\end{aligned} \quad \text{(Equation 3)}$$

we obtain by inserting Eqs. 1 and 2

$$\tilde{C}_{vv}(\omega) = \frac{B\tilde{\Gamma}_R(\omega)}{\left(\tilde{\Gamma}_v^+(\omega) + i\omega\right)\left(\tilde{\Gamma}_v^+(-\omega) - i\omega\right)} . \quad \text{(Equation 4)}$$

Equating the nonequilibrium and the surrogate VACF in Eqs. 26 and 27 leads to

$$\frac{\tilde{\Gamma}_R(\omega)}{\left(\tilde{\Gamma}_v^+(\omega) + i\omega\right)\left(\tilde{\Gamma}_v^+(-\omega) - i\omega\right)} = \frac{\tilde{\Gamma}(\omega)}{|\tilde{\Gamma}^+(\omega) + i\omega|^2} . \quad \text{(Equation 5)}$$

In the supporting material, we show that, for every correlation function $C_{vv}(t)$, we can determine a unique $\Gamma(t)$.

Memory kernel extraction

Multiplying the GLE Eq. 23 by $\dot{x}(t_0)$, averaging over the random force and integrating from t_0 to t leads to

$$(C_{vv}(t) - C_{vv}(0)) = -\int_0^t C_{vv}(s)G(t-s)ds, \quad \text{(Equation 6)}$$

where we used that $\langle \dot{x}(t_0)F_R(t)\rangle = 0$ (33–35), set $t_0 = 0$, and introduced the integral kernel

$$G(t) = \int_0^t \Gamma(s)ds . \quad \text{(Equation 7)}$$

To invert Eq. 6, we discretize it. Since $C_{vv}(t)$ is even but $G(t)$ is odd, we discretize $G(t)$ on half steps and $C_{vv}(t)$ on full steps and obtain (30)

$$G_{i+1/2} = \frac{2\left(C_{vv}^0 - C_{vv}^{i+1}\right)}{\Delta\left(C_{vv}^1 + C_{vv}^0\right)} - \sum_{j=1}^{i} G_{i-j+1/2}\frac{C_{vv}^{j+1} + C_{vv}^j}{C_{vv}^1 + C_{vv}^0}. \quad \text{(Equation 8)}$$

The kernel Γ_i is obtained by the discrete derivative $\Gamma_i = \frac{G_{i+1/2}+G_{i-1/2}}{\Delta}$ with the initial value $\Gamma_0 = 2G_{1/2}/\Delta$.

Two-point velocity distribution

A stationary Gaussian process is completely described by its two-point probability distribution as shown in the supporting material. Here we show that the joint and conditional velocity distributions only depend on the VACF. The joint probability to observe v_2 at time t_2 and v_1 at time t_1 can be written in terms of the velocity vector $\vec{v} = (v_1, v_2)^T$ as

$$p(v_2, t_2; v_1, t_1) = \frac{\exp\left(-\vec{v}^T\Sigma^{-1}(t_2-t_1)\vec{v}/2\right)}{\sqrt{2\pi|\Sigma(t_2-t_1)|}} \quad \text{(Equation 9)}$$

with $|\Sigma(t_2 - t_1)|$ denoting the determinant of the covariance matrix

$$\begin{aligned}\Sigma &= \begin{pmatrix}\langle v(t_1)v(t_1)\rangle & \langle v(t_1)v(t_2)\rangle \\ \langle v(t_1)v(t_2)\rangle & \langle v(t_2)v(t_2)\rangle\end{pmatrix} \\ &= \begin{pmatrix} C_{vv}(0) & C_{vv}(t_2-t_1) \\ C_{vv}(t_2-t_1) & C_{vv}(0)\end{pmatrix}.\end{aligned} \quad \text{(Equation 10)}$$

Using the normal velocity distribution

$$p(v) = \frac{\exp\left(\frac{-v^2}{2C_{vv}(0)}\right)}{\sqrt{2\pi C_{vv}(0)}}, \quad \text{(Equation 11)}$$

we obtain the conditional probability that $v(t_2) = v_2$ given $v(t_1) = v_1$, as

$$\begin{aligned}p(v_2, t_2|v_1, t_1) &= \frac{p(v_2, t_2; v_1, t_1)}{p(v_1, t_1)} \\ &= \frac{\exp\left(-\frac{[v_2 - v_1(C_{vv}(t_2-t_1))/C_{vv}(0)]^2}{\left(C_{vv}(0) - C_{vv}^2(t_2-t_1)/C_{vv}(0)\right)}\right)}{\sqrt{2\pi\left(C_{vv}(0) - C_{vv}^2(t_2-t_1)/C_{vv}(0)\right)}} .\end{aligned} \quad \text{(Equation 12)}$$

Thus, the joint and conditional distributions only depend on the VACF $C_{vv}(t)$.

Fitting of friction kernel

The extracted friction kernel of each individual cell is fitted to Eq. 29 by least-square minimization (using the curve fit function of python's *scipy*) of the first 0.2 s of the data, which allows to estimate the parameter standard deviation by the diagonal of the parameter covariance. The δ-peak in Eq. 29 leads to the initial kernel value $\Gamma_0 = 2a/\Delta + b$ where Δ denotes the discretization time (see section "memory kernel extraction"). Since single cells exhibit large variations of the friction kernels, we constrain a and b in Eq. 29 to be between 0.1 % and 99.9 % of Γ_0. The decay time τ is constrained to be between 0.05 and 3 s and the frequency Ω is constrained between 20 and 250 s^{-1}.

Discretized VACF including localization noise

To test whether the cell motion is actually described by the friction kernel (Eq. 29) and does not originate from the experimental finite time step or noise, we fit the experimental VACF of individual cells with an analytical model that accounts for finite time discretization and noise (30). Here, we explain the fitting procedure; the analytical expression for the

mean-squared displacement (MSD) using a friction kernel in the form of Eq. 29 is derived in the supporting material.

We denote discrete values of a function $f(t)$ as $f(i\Delta) = f_i = f^i$ and the discretization time step as Δ. After smoothing the data by averaging over consecutive positions to reduce the localization noise, as discussed in detail in the supporting material, the velocities at half time steps follow as

$$v_{i+\frac{1}{2}} = \frac{x_{i+1} - x_i}{\Delta}. \quad \text{(Equation 13)}$$

From the velocities, the VACF defined by Eq. 25 is calculated according to

$$C^i_{vv} = \frac{1}{N+1-i}\sum_{j=0}^{N-i} v_{j+\frac{1}{2}} v_{j+i+\frac{1}{2}}, \quad \text{(Equation 14)}$$

with N being the number of trajectory steps. To account for localization noise, we assume Gaussian uncorrelated noise of width σ_{loc} at every time step, which gives the noisy MSD as (30)

$$C^{\text{noise}}_{\text{MSD}}(t) = C^{\text{theo}}_{\text{MSD}}(t) + 2(1-\delta_{t0})\sigma^2_{\text{loc}}, \quad \text{(Equation 15)}$$

where $C^{\text{theo}}_{\text{MSD}}(t)$ is the theoretical expression for the model MSD given in the supporting material, Eq. S51, and δ_{t0} is the Kronecker delta reflecting the uncorrelated nature of the localization noise. Since the observed trajectories are sampled with a finite time step Δ, we discretize the relation

$$C_{vv}(t) = \frac{1}{2}\frac{d^2}{dt^2}C_{\text{MSD}}(t), \quad \text{(Equation 16)}$$

which leads to

$$C^{\text{fit}}_{vv}(i\Delta) = \frac{C^{\text{noise}}_{\text{MSD}}((i+1)\Delta) - 2C^{\text{noise}}_{\text{MSD}}(i\Delta) + C^{\text{noise}}_{\text{MSD}}((i-1)\Delta)}{2\Delta^2}. \quad \text{(Equation 17)}$$

Finally, fits are performed by minimizing the cost function

$$E_{\text{cost}} = \sum_{i=0}^{n}\left(C^{\text{exp}}_{vv}(i\Delta) - C^{\text{fit}}_{vv}(i\Delta)\right)^2 \quad \text{(Equation 18)}$$

with SciPy's least squares function in python and using Eq. 17 to determine $C^{\text{fit}}_{vv}(t)$ at discrete time points. As the MSD and VACF follow from the GLE Eq. 23 and the friction kernel (Eq. 29), the parameters to optimize are the kernel parameters a, b, τ, Ω, the mean-squared velocity B, and the localization noise width σ_{loc}. The data is fitted up to 0.2 s to disregard the noisy part of the VACF (see the supporting material for details). The cluster analysis is performed on the reduced parameter sets a, b, τ, Ω, B, which are obtained from the direct fit of Eq. 29 to the extracted kernel from the data.

Cluster analysis

The friction kernel in Eq. 29 contains four parameters; together with the mean-squared velocity B, each individual cell is characterized by five parameters. We perform an X-means cluster analysis (55), which is a generalization of the k-means algorithm (56). The k-means algorithm assigns unlabeled data to a predetermined number of k clusters by minimizing distances to the cluster centers. In the X-means algorithm, the number of clusters is not predetermined; we allow cluster numbers from 2 to 20. The algorithm starts with the minimal number of clusters and finds the cluster centers using k-means. It then splits every cluster into two subclusters whose centers are again determined by k-means. New subclusters are accepted if they improve the clustering quality accounting for the increased number of parameters. For this, we use the minimal noiseless description length criterion (57,58). We use an implementation of the X-means algorithm in Python (59) and use individual cell parameters as initial cluster centers (60). The X-means algorithm can converge to different final results depending on the initial cluster centers. Thus, we use all $59 \cdot 58/2$ possible combinations of initial cluster centers and use the result that occurs most often. We rescale each parameter by the median of its distribution.

Markovian embedding

A similar kernel to Eq. 29, namely

$$\Gamma(t) = 2a\delta(t) + be^{-t/\tau}\left(\cos(\Omega t) + \frac{1}{\tau\Omega}\sin(\Omega t)\right), \quad \text{(Equation 19)}$$

can be derived from a system of harmonically coupled degrees of freedom. In fact, Eq. 19 becomes equivalent to Eq. 29 if the oscillation period $1/\Omega$ is much smaller than the decay time τ, which is the case for the extracted algae kernels. The Hamiltonian describing the coupled degrees of freedom takes the form

$$H = \frac{m}{2}v^2 + \frac{m_y}{2}v_y^2 + \frac{K}{2}(x-y)^2, \quad \text{(Equation 20)}$$

where m_i are the effective masses of the two degrees of freedom and $K = bm$ is the harmonic coupling strength. In the presence of friction, quantified by friction coefficients γ_i, and coupling the degrees of freedom to a heat bath at temperature T, the coupled equations of motion are given by

$$\begin{aligned} \dot{x}(t) &= v(t) \\ m\dot{v}(t) &= -\gamma_x v + bm(y(t) - x(t)) + F_{Rx}(t) \\ \dot{y}(t) &= v_y(t) \\ m_y\dot{v}_y(t) &= -\gamma_y v_y + bm(x(t) - y(t)) + F_{Ry}(t), \end{aligned} \quad \text{(Equation 21)}$$

where $F_{Rx}(t)$ and $F_{Ry}(t)$ are random forces with zero mean and second moment $\langle F_{Ri}(0)F_{Rj}(t)\rangle = \delta_{ij}2\gamma_i k_B T\delta(t)$. In the supporting material, it is shown that the coupled equations of motion Eq. 21 are equivalent to a GLE in the form of Eq. 23 for the coordinate $x(t)$ with a memory kernel $\Gamma(t)$ given by Eq. 19 (61). The friction of the first degree of freedom leads to the δ-contribution of $\Gamma(t)$ and the harmonic coupling to the second degree of freedom leads to the oscillating exponentially decaying contribution. The parameters of Eq. 21 translate into the parameters of Eq. 19 as

$$\begin{aligned} a &= \frac{\gamma_x}{m} \\ \tau &= 2\frac{m_y}{\gamma_y} \\ \Omega &= \sqrt{\frac{bm}{m_y} - \frac{1}{\tau^2}}. \end{aligned} \quad \text{(Equation 22)}$$

RESULTS

Experimental trajectories

Our analysis is based on videos of 59 CR cells that are strongly confined between two glass plates separated by a distance similar to the cell diameter $\sim 10\ \mu$m, which resembles the natural habitat of CR in soil (45) and simplifies the recording of long two-dimensional trajectories, as the cells cannot move out of the image plane of the microscope objective (see sections "cell growth and sample preparation" and "recording of trajectories" for experimental

details). Videos that resolve the flagella motion are shown in the supporting material and reveal two different types of flagellar motion (51). In one type, the flagella move synchronously as in a breaststroke called "synchro" (Fig. 1 *a* and Video S1); in the other type, the flagella move asynchronously, which results in a wobbling cell motion called "wobbler" (Fig. 1 *b* and Video S2). The emergence of two different swimming modes reflects cell-size variation and constitutes the tactile cell response to the confining surfaces (51), where the synchros tend to exhibit slightly larger cell bodies and therefore are more strongly confined. The confining surfaces are coated with an anti-adhesive polymer brush to prevent sticking of cells to the surfaces. The strong confinement leads to cell-surface friction, which is fully accounted for by the GLE analysis. In fact, unconfined CR cells do not exhibit distinct synchro and wobbler swimming modes (51,52). Switching events between synchronous and asynchronous flagella motion are never observed and thus are negligible. We use the classification into synchros and wobblers based on the flagella motion in the high-resolution video data as a test of our classification method that is based on the cell-center trajectories.

Theoretical trajectory model

The experiments yield two-dimensional trajectories $x(t), y(t)$ for the cell center position, which we describe by the GLE

$$\ddot{x}(t) = -\int_{t_0}^{t} \Gamma_v(t-t')\dot{x}(t')dt' + F_R(t) \qquad \text{(Equation 23)}$$

with an identical equation for $y(t)$. Here, $\ddot{x}(t) = \dot{v}(t)$ denotes the acceleration of the cell position, $\Gamma_v(t)$ is a memory kernel that describes how the acceleration at time t depends on the cell velocity $\dot{x}(t') = v(t')$ at previous times and therefore accounts for non-Markovian friction effects, and $F_R(t)$ is a random force that describes interactions with the surrounding and within the interior of the cell. Since the experimental system is isotropic and homogeneous in space, no deterministic force term appears in the GLE. In fact, the GLE in Eq. 23 can be derived by projection at time t_0 from the underlying many-body Hamiltonian even in the presence of nonequilibrium effects, which obviously are present for living organisms (33–35,37).

If the cell motion can be described as a Gaussian process, which for CR cells is suggested by the fact that the single-cell velocity distributions are perfectly Gaussian, as will be demonstrated further below, the random force is a Gaussian process with correlations given by

$$\langle F_R(t)F_R(0)\rangle = B\Gamma_R(t), \qquad \text{(Equation 24)}$$

where $B = \langle v^2\rangle$ denotes the mean-squared cell velocity and the symmetric random-force kernel is denoted as $\Gamma_R(t)$. In this case, the equation of motion is linear and there is no coupling between the motion in x and y direction, and we thus average all cell-trajectory data over the two directions.

For an equilibrium system, the fluctuation dissipation theorem (FDT) predicts $\Gamma_R(t) = \Gamma_v(|t|)$ with the mean-squared velocity given by $B = k_BT/m$ according to the equipartition theorem, where m is the mass of the moving object and k_BT denotes the thermal energy (33,34). For living cells, both FDT and equipartition theorem do not hold in general and thus there is no a priori reason why $\Gamma_v(|t|)$ and $\Gamma_R(t)$ should be equal (36,62). Nevertheless, one can construct a surrogate model with an effective kernel $\Gamma(|t|) = \Gamma_R(t) = \Gamma_v(|t|)$ that exactly reproduces the dynamics described by the nonequilibrium GLE with $\Gamma_R(t) \neq \Gamma_v(|t|)$. This can be most easily seen by considering the VACF defined by

$$C_{vv}(t) = \langle v(0)v(t)\rangle, \qquad \text{(Equation 25)}$$

whose Fourier transform $\tilde{C}_{vv}(\omega) = \int_{-\infty}^{\infty} e^{-i\omega t}C_{vv}(t)dt$ follows from Eqs. 23 and 24 as (30)

$$\tilde{C}_{vv}(\omega) = \frac{B\tilde{\Gamma}_R(\omega)}{\left(\tilde{\Gamma}_v^+(\omega)+i\omega\right)\left(\tilde{\Gamma}_v^+(-\omega)-i\omega\right)}, \qquad \text{(Equation 26)}$$

where $\tilde{\Gamma}_v^+(\omega)$ denotes the single-sided Fourier transform of $\Gamma_v(t)$ (see section Velocity autocorrelation function for the derivation). The VACF of the surrogate model with $\Gamma(|t|) = \Gamma_R(t) = \Gamma_v(|t|)$ follows from Eq. 26 as

$$\tilde{C}_{vv}^{\text{sur}}(\omega) = \frac{B\tilde{\Gamma}(\omega)}{(\tilde{\Gamma}^+(\omega)+i\omega)(\tilde{\Gamma}^+(-\omega)-i\omega)}. \qquad \text{(Equation 27)}$$

For each combination of $\Gamma_R(t)$ and $\Gamma_v(t)$, there is a unique $\Gamma(t)$ that produces the same VACF; i.e., for which $\tilde{C}_{vv}^{\text{sur}}(\omega) = \tilde{C}_{vv}(\omega)$ holds (see the supporting material for the derivation) and which can be uniquely extracted from trajectories via the VACF (as shown in section "memory kernel extraction"). Since the VACF completely determines the dynamics of a Gaussian system, as shown in section "two-point velocity distribution" and in more detail in the supporting material, this implies that the extracted effective kernel $\Gamma(t)$ not only describes the VACF exactly but also characterizes the system completely (63). In fact, recent work, where the nonequilibrium GLE is derived from a suitably chosen time-dependent Hamiltonian, shows that, for Gaussian nonequilibrium observables, the condition $\Gamma_R(t) = \Gamma_v(|t|)$ is actually satisfied (37), in line with our method that is based on extracting an effective kernel $\Gamma(t)$.

Trajectories and velocity distributions

Due to the asynchronous flagella motion of the wobblers, the cells turn in the flagella-beating rhythm and exhibit

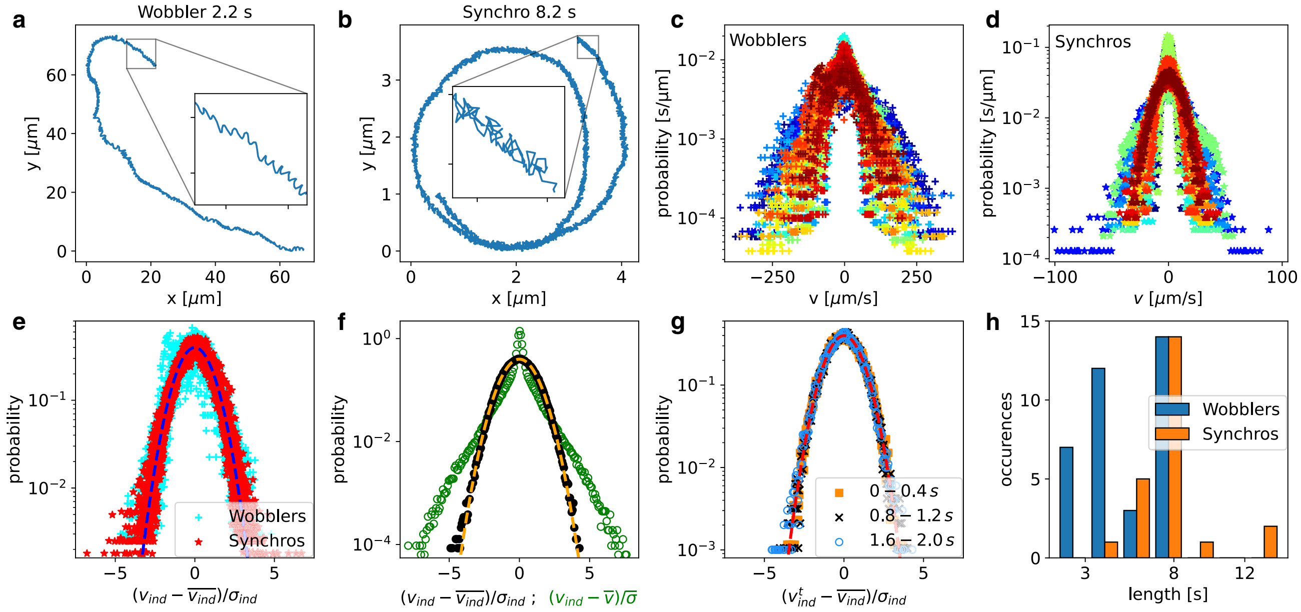

FIGURE 2 Cell-center trajectories. Exemplary cell-center trajectory (54) (*a*) of a wobbler of duration 2.2 s and (*b*) of a synchro of duration 8.2 s. The insets show trajectory fragments of duration 0.2 s each. Velocity distributions of (*c*) wobblers and (*d*) synchros, individual cells are distinguished by color. (*e*) Velocity distributions of individual cells rescaled by subtracting the mean velocity of individual cells $\overline{v_{\mathrm{ind}}}$ and dividing by their standard deviation σ_{ind} for wobblers (*cyan crosses*) and synchros (*red stars*). The dashed line is the normal distribution. (*f*) Mean velocity distribution averaged over all cells (wobblers and synchros). For the green circles, the cell velocities are rescaled by subtracting the ensemble mean velocity $\overline{v}$ and dividing by the ensemble standard deviation $\overline{\sigma}$; for the black circles, the cell velocities are rescaled by subtracting the mean velocity of individual cells $\overline{v_{\mathrm{ind}}}$ and dividing by their standard deviation σ_{ind} as in (*e*). The normal distribution is indicated by a dashed line. (*g*) Individually rescaled velocity distributions of all cells for three different time windows. (*h*) Distribution of recorded trajectory lengths for wobblers and synchros. To see this figure in color, go online.

monotonically forward-moving wiggly trajectories, as shown in Fig. 2 *a*. In contrast, the synchronous flagella beating of synchros leads to fast switching between forward and backward motion, as shown in Fig. 2 *b*. As a consequence, synchros exhibit much slower net-forward motion than wobblers, as seen in Fig. 2 *a* and *b*, where trajectories with total duration 2.2 s (wobbler) and 8.2 s (synchro) are compared. Synchros also exhibit a somewhat narrower instantaneous velocity distribution, as seen in Fig. 2 *c* and *d*. However, a differentiation of the two cell types solely based on their speed does not work, as we will show later.

Even though individual cells exhibit pronounced variations in their velocity distributions, as seen from the large spread in Fig. 2 *c* and *d*, their velocity distributions are Gaussian, as demonstrated in Fig. 2 *e*: When subtracting from the cell velocities the mean velocity of each individual cell and dividing by the corresponding velocity standard deviation, $(v_{\mathrm{ind}} - \overline{v_{\mathrm{ind}}})/\sigma_{\mathrm{ind}}$, all individual velocity distributions collapse onto the Gaussian (normal) distribution (dashed line in Fig. 2 *e*). In contrast, when subtracting from the cell velocities the cell-ensemble mean velocity and dividing by the cell-ensemble standard deviation, $(v_{\mathrm{ind}} - \overline{v})/\sigma$, the velocity distribution averaged over all cells deviates strongly from a Gaussian (green circles in Fig. 2 *f*). In contrast, the individually rescaled velocity distribution averaged over all cells (black dots) perfectly agrees with the Gaussian normal distribution (dashed line in Fig. 2 *f*). Thus, single cells exhibit perfectly Gaussian velocity distributions, which suggests that the GLE with Gaussian noise is appropriate to analyze experimental single-cell trajectories and that the condition $\Gamma_R(t) = \Gamma_v(|t|)$ holds (37).

The GLE in Eq. 23 features time-independent parameters and thus describes a stationary process. That the cell velocity distribution does not change over the observational time is demonstrated in Fig. 2 *g*, where velocity distributions in three consecutive time intervals are compared (again subtracting the individual cell mean velocities and dividing by the corresponding velocity deviations of the entire trajectory). This suggests that the motion of individual CR algae can indeed be modeled by the GLE in Eq. 23. In this context, it is to be noted that wobblers are relatively fast and tend to move out of the camera window more quickly than synchros, leading to slightly shorter wobbler trajectories, as shown in Fig. 2 *h*.

Trajectory analysis and friction-kernel extraction

Trajectories are standardly characterized by the VACF or by the MSD

$$C_{\mathrm{MSD}}(t) = \langle (x(0) - x(t))^2 \rangle . \qquad \text{(Equation 28)}$$

Although the VACF is simply the curvature of the MSD, $C_{vv}(t) = \frac{1}{2}\frac{d^2}{dt^2}C_{\mathrm{MSD}}(t)$, the MSD and the VACF highlight

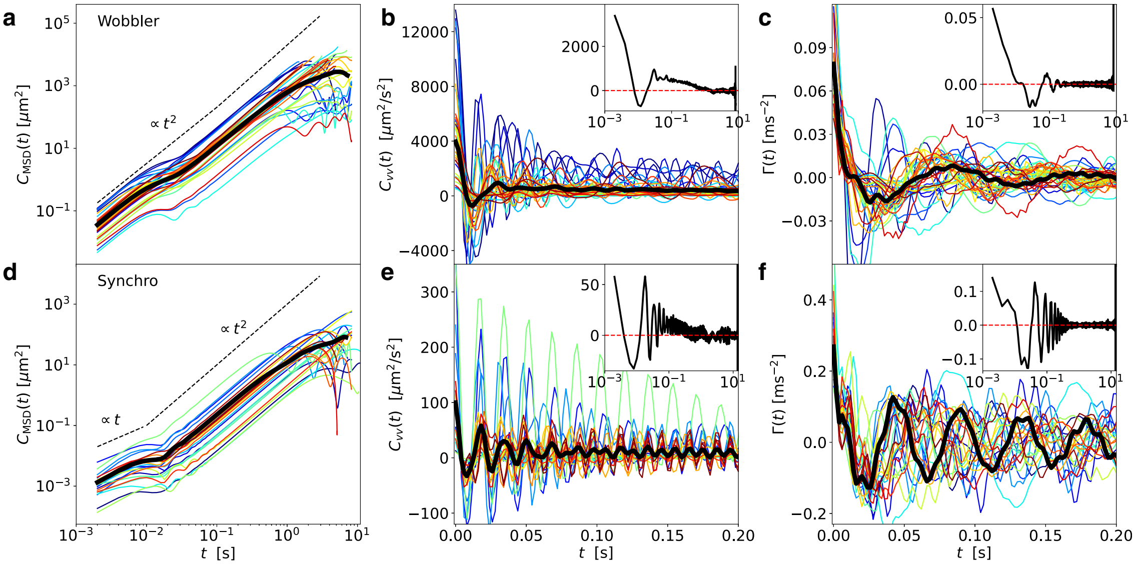

FIGURE 3 Results for the MSD, the VACF, and the friction kernel for wobblers and synchros. Results for the MSD, $C_{\rm MSD}(t)$ defined in Eq. 28; the VACF, $C_{vv}(t)$ defined in Eq. 24; and the friction kernel $\Gamma(t)$, extracted according to Eq. 8, for wobblers in (*a*)–(*c*) and synchros in (*d*)–(*f*). Different colors represent results for individual cells; the black lines in (*a*), (*b*), (*d*), and (*e*) denote the average over all cells. For the friction kernels, the black line is computed from the average VACF. The dashed lines in (*a*) and (*d*) indicate ballistic and diffusive scaling. Insets show the long-time behavior of the average quantities on a lin-log scale. To see this figure in color, go online.

different aspects of the trajectories. In fact, the different propulsion modes of wobblers and synchros lead to drastically different MSDs: the wobblers exhibit ballistic behavior $C_{\rm MSD}(t) \propto t^2$ on both short and long timescales (Fig. 3 *a*) with an intermediate crossover at $t \sim 0.02$ s. In contrast, the forward-backward motion of the synchros leads to short-time diffusive behavior $C_{\rm MSD}(t) \propto t$ up to $t \sim 0.01$ s followed by a long-time ballistic regime for $t > 0.02$ s (Fig. 3 *d*). The transition to the intermediate ballistic regime occurs for both synchros and wobblers around the flagella oscillation period of the order of $t \sim 0.02$ s; the MSD for shorter times is dominated by the flagella motion and for longer times by the ballistic net-forward motion. The transition to asymptotic diffusive behavior, expected for long times, is for the synchros observed for $t > 2s$ in extended low-resolution microscopy data, whereas wobblers stay in the ballistic regime for the entire observation time of tens of seconds (see the supporting material). As wobblers move faster than synchros, their absolute VACF values are higher compared to the synchros, as seen in Fig. 3 *b* and *e*. The synchros exhibit less variation of the VACF among individual cells, which leads to slowly decaying oscillations in the VACF averaged over all cells (black line in Fig. 3 *e* compared to Fig. 3 *b*). These oscillations reflect the flagella-beating cycle.

We extract effective friction kernels $\Gamma(t)$ from the VACF of individual cells, as described in section "memory kernel extraction." The results, shown as colored lines in Fig. 3 *c* and *f*, demonstrate that the algal motion deviates strongly from the simple persistent random walk model, which is widely used to describe the motion of cells (28,29,32) and which in the GLE formulation would correspond to $\Gamma(t)$ exhibiting a delta peak at $t = 0$ and otherwise being zero. The extracted memory kernels also reveal a substantially higher friction for synchros compared to wobblers, in line with the fact that synchros are larger and thus interact more strongly with the confining surfaces.

Comparing Fig. 3 *b* with Fig. 3 *c* or Fig. 3 *e* with Fig. 3 *f*, one notes that the oscillation period of the friction kernel is substantially longer than that of the VACF. The complex relation between the kernel and the VACF is discussed in the supporting material, where it is shown that the extracted values of the kernel decay time and oscillation amplitude achieve high directionality and speed of CR cells.

For both wobblers and synchros, the friction kernels exhibit an initial sharp peak followed by a decaying oscillation and are well described by

$$\Gamma(t) = 2a\delta(t) + be^{-t/\tau}\cos(\Omega t), \qquad \text{(Equation 29)}$$

with δ-peak amplitude a, oscillation amplitude b, exponential decay time τ, and oscillation frequency Ω. This is demonstrated in Fig. 4 *c* and *f* for one exemplary synchro and wobbler, where we compare the extracted memory kernels with fits according to Eq. 29, see section "fitting of friction kernel" for details. From these fits, we thus obtain four memory kernel parameters for each cell.

Before further analysis of the obtained individual cell parameters, we test whether the GLE Eq. 23 actually describes the cell motion. We thus compare the experimental MSD of a single wobbler and synchro in Fig. 4 *a* and *d* (orange dots) with the analytical prediction based on the GLE using the fit result for the friction kernel and the mean-squared velocity

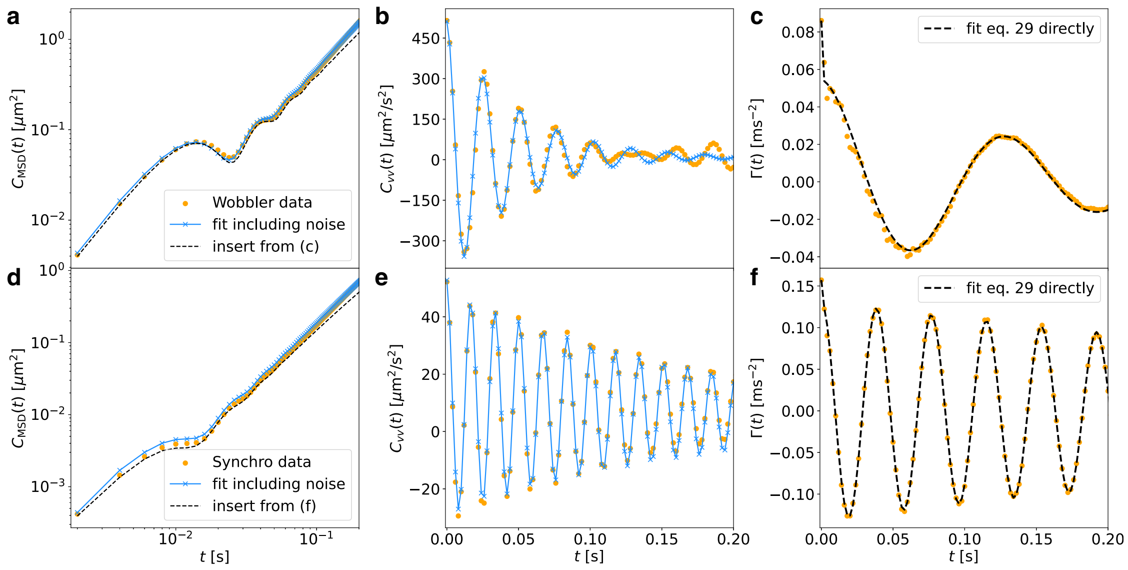

FIGURE 4 Check the accuracy of the GLE for a single wobbler and synchro. Results for the MSD, $C_{\mathrm{MSD}}(t)$; VACF, $C_{vv}(t)$; and friction kernel $\Gamma(t)$, of a single (*a–c*) wobbler and (*d–f*) synchro (*orange dots*). The black dashed lines in (*c*) and (*f*) denote fits of Eq. 29 to the extracted friction kernel determining the individual cell parameters shown in Fig. 5. The black dashed lines in (*a*) and (*d*) denote the analytical result Eq. S51 obtained from the friction-kernel fit in (*c*) and (*f*) and the mean-squared velocity $B = C_{vv}(0)$. Blue crosses in (*b*) and (e) denote a fit of the discretized expression for the VACF, including localization noise, (Eq. 17); blue crosses in (*a*) and (*d*) denote the corresponding prediction for the MSD, Eq. 15, using the same parameters as in (*b*) and (*e*); blue crosses are connected by blue straight lines. To see this figure in color, go online.

$B = C_{vv}(0)$ (black broken lines, the derivation of the analytical MSD expression is given in the supporting material). The agreement is very good, meaning that the memory extraction works well, which demonstrates that the GLE is an accurate model for the motion of organisms.

The MSD and VACF calculated from the GLE neglect the finite experimental recording time step of 0.002 s, and they also neglect the localization noise of the cell position, due to the finite spatial resolution of the microscopy images and the projection of a three dimensional object onto a two-dimensional point position (30) (see section "discretized VACF including localization noise" for details). The blue crosses in Fig. 4 *b* and *e* represent fits of the GLE-based analytical expression for the VACF, which includes localization noise and discretization effects, given in Eq. 17, to the experimental data. The blue crosses in Fig. 4 *a* and *d* show the corresponding MSD results with the same parameters according to Eq. 15. The agreement between experimental data and the discretized model is perfect, and the fitted localization noise strength, defined in Eq. 15, is of the order of $\sigma_{\mathrm{loc}} \sim 0.02\ \mu$m, similar to the pixel size, as expected (see discussion in the supporting material). This means that temporal and spatial discretization effects in the experimental data can be straightforwardly incorporated in the GLE model.

Clustering of single-cell parameters

The GLE Eq. 23 in conjunction with the random-force strength B defined in Eq. 24 and the effective friction kernel Eq. 29 has five parameters. This gives rise to 10 distinct two-dimensional projections, which are shown in Fig. 5. Each data point corresponds to a single cell. The parameters exhibit substantial spread among individual cells, but an unambiguous separation between wobblers and synchros, here colored in blue and red, is not obvious. As can already be seen in Fig. 3 *c* and *f*, the friction amplitudes a and b are larger for the synchros, whereas the mean-squared velocity B is larger for wobblers, which leads to a separation of the two populations in Fig. 5 *b* and *c*. Each flagellar beating cycle leads to a net forward cell motion, which is reflected by the positive correlation between memory oscillation frequency Ω and mean-squared velocity B in Fig. 5 *g* for each cell type. The uncertainty of the parameters in Fig. 5, estimated from the diagonal fit covariances, is rather low except for the decay time τ, as seen in Fig. S5 in the supporting material. Since the parameter τ contributes only marginally to the clustering confidence, as demonstrated by the results in Figs. 5 and S4, we conclude that our cluster analysis is not affected by parameter uncertainties.

We perform an unbiased cluster analysis using the X-means algorithm (55), which is a general version of the k-means algorithm (56) that self-consistently determines the optimal number of clusters (details are given in section "cluster analysis"). Applying this unbiased cluster analysis to the single-cell parameters in five-dimensional space, we obtain two distinct groups, which perfectly coincide with the assignment into wobblers and synchros from visual analysis of the flagellar motion in the video data (51). This means that we can classify the cells by just knowing their center of mass trajectories with an accuracy of 100 %. In comparison,

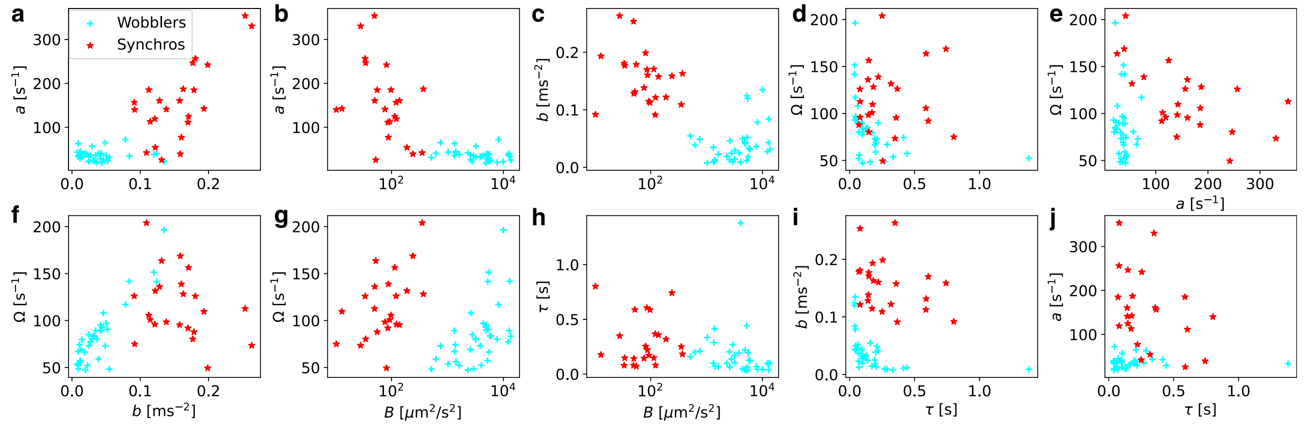

FIGURE 5 Scatter correlation plots of individual CR cell parameters. These consist of the friction-kernel parameters (a, b, τ, Ω) defined in Eq. 29 and the mean-squared velocity B. All parameters except B are presented on a linear scale. Synchros are shown in red and wobblers in cyan according to our cluster analysis, which perfectly matches a categorization based on the visual analysis of flagella motion. Error estimates of the extracted parameters are omitted here for clarity and shown in Fig. S5. To see this figure in color, go online.

a cluster analysis solely based on the mean-squared velocity B leads to an accuracy of only 69 %, whereas a cluster analysis using the first two principal-component analysis components leads to an accuracy of 90 % (see the supporting material). Using only the four friction-kernel parameters for a cluster analysis without the mean-squared velocity B still reaches an accuracy of 92 %. This clearly demonstrates that the GLE model, which parameterizes cell motion based on friction-kernel parameters in Eq. 29 together with the mean-squared velocity B, allows for accurate classification of cells based only on their motion.

DISCUSSION AND CONCLUSIONS

We demonstrate that the rather complex motion of individual CR algae can be accurately described by the GLE Eq. 23 and extract all GLE model parameters for individual algae from their cell-center trajectories in a data-driven manner. Based on the extracted GLE parameters, we detect two distinct algae classes by an unbiased cluster analysis; this unsupervised clustering result is confirmed by comparison with a categorization based on visual inspection of the flagella beating patterns. Our method is applicable to any kind of cells and even higher organisms if the motion is a Gaussian process. Cell and animal motion often exhibits aging effects (64), which in the GLE framework would show up as very slowly decaying contributions to the memory function. To accurately extract slowly decaying memory functions from data, very long trajectories would be needed. Since the GLE Eq. 23 is not restricted to positional degrees of freedom, our approach can be applied to any observable (for instance, cell extension or deformation). As the only inputs needed are trajectories, our method requires minimal interaction with the organisms and can be easily used as a stand-alone tool or to improve existing machine-learning algorithms for cell classification.

Additionally, our approach allows for a mechanistic interpretation of cell-motion characteristics. In fact, a friction-kernel model that is very similar to Eq. 29 and describes the data equally well can be derived from the equation of motion of two elastically coupled objects (see section "markovian embedding" and the supporting material for details). Without further experimental input, our approach does not reveal what these objects are, so we can only speculate that the elastic coupling between the cell body and the flagella, which presumably involves the connection between the flagellar basal bodies by the distal striated fiber (schematically shown in Fig. 1 *c*), causes the slowly decaying oscillations in the memory kernel. This seems in line with previous models for CR algae motion and flagella synchronization (65,66). Alternatively, the kernel oscillations could also be caused by some chemical or hydrodynamic feedback loop. Clearly, more experiments that resolve the relative motion of the cell center and the flagella are needed to resolve these issues.

Our extraction of GLE parameters from cell trajectories yields a single effective memory kernel and does not allow detection of the nonequilibrium character of cell motion, in agreement with recent general arguments (63). Conversely, based on an explicit nonequilibrium model for cell motion, it is rather straightforward to derive the GLE Eq. 23 and the functional form of the extracted kernel (Eq. 29), as shown in the supporting material. A similar GLE model can also be derived for motion in a confining potential to describe confined neurons (5) or bacteria moving in mucus (67) (see the supporting material for details).

As a final note, we mention that the angular orientation of CR algae can be accurately extracted from their cell-center trajectory, as shown in the supporting material. Thus, the orientational cell dynamics is included in our GLE model.

In summary, our approach allows for cell classification by positional cell-center trajectories or any other kind of

time-series data and at the same time for interpretation of the motion pattern in terms of intracellular interactions. We anticipate numerous applications in biology and medicine that require the label-free distinction of individual cells and organisms.

SUPPORTING MATERIAL

Supporting Material can be found online at https://doi.org/10.1016/j.bpj.2024.03.023.

AUTHOR CONTRIBUTIONS

D.M. and P.S. designed and performed the experiments. S.B. contributed analytic tools and analyzed data. A.K. and R.R.N. designed the models, analyzed the experimental data, and wrote the manuscript.

ACKNOWLEDGMENTS

We acknowledge funding by the Deutsche Forschungsgemeinschaft (DFG) through grant CRC 1449 Dynamic Hydrogels at Biointerfaces, project ID 431232613, project A03, by the ERC Advanced Grant NoMaMemo no. 835117, and by the Infosys Foundation. D.M. and P.S. acknowledge support from the DBT/Wellcome Trust India Alliance Fellowship (grant number IA/I/16/1/502356) awarded to P.S.

DECLARATION OF INTERESTS

The authors declare no competing interests.

REFERENCES

1. Gessert, N., M. Bengs, …, D. B. Ellebrecht. 2019. Deep transfer learning methods for colon cancer classification in confocal laser microscopy images. *Int. J. Comput. Assist. Radiol. Surg.* 14:1837–1845.
2. Teng, M. W. L., S. F. Ngiow, …, M. J. Smyth. 2015. Classifying cancers based on T-cell infiltration and PD-L1. *Cancer Res.* 75:2139–2145.
3. Kang, R., B. Park, …, K. Chen. 2020. Single-cell classification of foodborne pathogens using hyperspectral microscope imaging coupled with deep learning frameworks. *Sensor. Actuator. B Chem.* 309, 127789.
4. Davis, R. O., and R. J. Siemers. 1995. Derivation and reliability of kinematic measures of sperm motion. *Reprod. Fertil. Dev.* 7:857–869.
5. Zeng, H., and J. R. Sanes. 2017. Neuronal cell-type classification: challenges, opportunities and the path forward. *Nat. Rev. Neurosci.* 18:530–546.
6. Zhang, L., F. Wang, …, Y. Zhu. 2020. Classifying Cell Types with DNA-Encoded Ligand–Receptor Interactions on the Cell Membrane. *Nano Lett.* 20:3521–3527.
7. Sanz, I., C. Wei, …, F. E.-H. Lee. 2019. Challenges and opportunities for consistent classification of human B cell and plasma cell populations. *Front. Immunol.* 10:2458.
8. Saliba, A.-E., A. J. Westermann, …, J. Vogel. 2014. Single-cell RNA-seq: advances and future challenges. *Nucleic Acids Res.* 42:8845–8860.
9. Kolodziejczyk, A. A., J. K. Kim, …, S. A. Teichmann. 2015. The technology and biology of single-cell RNA sequencing. *Mol. Cell.* 58:610–620.
10. Papalexi, E., and R. Satija. 2018. Single-cell RNA sequencing to explore immune cell heterogeneity. *Nat. Rev. Immunol.* 18:35–45.
11. Qi, R., A. Ma, …, Q. Zou. 2020. Clustering and classification methods for single-cell RNA-sequencing data. *Briefings Bioinf.* 21:1196–1208.
12. Chen, C. L., A. Mahjoubfar, …, B. Jalali. 2016. Deep learning in label-free cell classification. *Sci. Rep.* 6:21471.
13. Shen, S., K. Syal, …, S. Wang. 2015. Note: An automated image analysis method for high-throughput classification of surface-bound bacterial cell motions. *Rev. Sci. Instrum.* 86, 126104.
14. Liu, X., and Y.-T. Chang. 2022. Fluorescent probe strategy for live cell distinction. *Chem. Soc. Rev.* 51:1573–1591.
15. Sbalzarini, I. F., J. Theriot, and P. Koumoutsakos. 2002. Machine learning for biological trajectory classification applications. *In* Studying Turbulence Using Numerical Simulation Databases-IX: Proceedings of the 2002 Summer Program.
16. Boedeker, H. U., C. Beta, …, E. Bodenschatz. 2010. Quantitative analysis of random ameboid motion. *Europhys. Lett.* 90, 28005.
17. Pohl, O., M. Hintsche, …, H. Stark. 2017. Inferring the chemotactic strategy of P. putida and E. coli using modified Kramers-Moyal coefficients. *PLoS Comput. Biol.* 13, e1005329.
18. Maiuri, P., J.-F. Rupprecht, …, R. Voituriez. 2015. Actin flows mediate a universal coupling between cell speed and cell persistence. *Cell.* 161:374–386.
19. Heuzé, M. L., P. Vargas, …, A. M. non-Lennon-Duménil. 2013. Migration of dendritic cells: physical principles, molecular mechanisms, and functional implications. *Immunol. Rev.* 256:240–254.
20. Liu, Y.-J., M. Le Berre, …, M. Piel. 2015. Confinement and low adhesion induce fast amoeboid migration of slow mesenchymal cells. *Cell.* 160:659–672.
21. da Silva, C. L., L. M. Petry, and V. Bogorny. 2019. A survey and comparison of trajectory classification methods. *In* 2019 8th Brazilian Conference on Intelligent Systems (BRACIS) IEEE, pp. 788–793.
22. Amselem, G., M. Theves, …, C. Beta. 2012. A stochastic description of Dictyostelium chemotaxis. *PLoS One.* 7, e37213.
23. Selmeczi, D., L. Li, …, H. Flyvbjerg. 2008. Cell motility as random motion: A review: Cell motility as random motion. *Eur. Phys. J. Spec. Top.* 157:1–15.
24. Selmeczi, D., S. Mosler, …, H. Flyvbjerg. 2005. Cell motility as persistent random motion: theories from experiments. *Biophys. J.* 89:912–931.
25. Codling, E. A., M. J. Plank, and S. Benhamou. 2008. Random walk models in biology. *J. R. Soc. Interface.* 5:813–834.
26. Pedersen, J. N., L. Li, …, H. Flyvbjerg. 2016. How to connect time-lapse recorded trajectories of motile microorganisms with dynamical models in continuous time. *Phys. Rev. E.* 94, 062401.
27. Dieterich, P., R. Klages, …, A. Schwab. 2008. Anomalous dynamics of cell migration. *Proc. Natl. Acad. Sci. USA.* 105:459–463.
28. Gail, M. H., and C. W. Boone. 1970. The locomotion of mouse fibroblasts in tissue culture. *Biophys. J.* 10:980–993.
29. Wright, A., Y.-H. Li, and C. Zhu. 2008. The differential effect of endothelial cell factors on in vitro motility of malignant and non-malignant cells. *Ann. Biomed. Eng.* 36:958–969.
30. Mitterwallner, B. G., C. Schreiber, …, R. R. Netz. 2020. Non-Markovian data-driven modeling of single-cell motility. *Phys. Rev. E.* 101, 032408. https://doi.org/10.1103/PhysRevE.101.032408.
31. Li, L., E. C. Cox, and H. Flyvbjerg. 2011. 'Dicty dynamics': Dictyostelium motility as persistent random motion. *Phys. Biol.* 8, 046006.
32. Li, L., S. F. Nørrelykke, and E. C. Cox. 2008. Persistent cell motion in the absence of external signals: a search strategy for eukaryotic cells. *PLoS One.* 3, e2093.
33. Mori, H. 1965. Transport, Collective Motion, and Brownian Motion. *Prog. Theor. Phys.* 33:423–455. https://doi.org/10.1143/PTP.33.423.
34. Zwanzig, R. 1961. Memory effects in irreversible thermodynamics. *Phys. Rev.* 124:983–992.
35. Ayaz, C., L. Scalfi, …, R. R. Netz. 2022. Generalized Langevin equation with a nonlinear potential of mean force and nonlinear memory

friction from a hybrid projection scheme. *Phys. Rev. E.* 105, 054138. https://doi.org/10.1103/PhysRevE.105.054138.

36. Mizuno, D., C. Tardin, …, F. C. MacKintosh. 2007. Nonequilibrium mechanics of active cytoskeletal networks. *Science.* 315:370–373.
37. Netz, R. R. 2023. Derivation of the non-equilibrium generalized Langevin equation from a generic time-dependent Hamiltonian. Preprint at arXiv. https://doi.org/10.48550/arXiv.2310.00748.
38. Bechinger, C., R. Di Leonardo, …, G. Volpe. 2016. Active particles in complex and crowded environments. *Rev. Mod. Phys.* 88, 045006.
39. Viswanathan, G. M., M. G. Da Luz, …, H. E. Stanley. 2011. The Physics of Foraging: An Introduction to Random Searches and Biological Encounters. Cambridge University Press.
40. Romanczuk, P., M. Bär, …, L. Schimansky-Geier. 2012. Active Brownian particles: From individual to collective stochastic dynamics. *Eur. Phys. J. Spec. Top.* 202:1–162.
41. Ramaswamy, S. 2010. The mechanics and statistics of active matter. *Annu. Rev. Condens. Matter Phys.* 1:323–345.
42. Brockmann, D., L. Hufnagel, and T. Geisel. 2006. The scaling laws of human travel. *Nature.* 439:462–465.
43. Tailleur, J., and M. E. Cates. 2008. Statistical mechanics of interacting run-and-tumble bacteria. *Phys. Rev. Lett.* 100, 218103.
44. Mitterwallner, B. G., L. Lavacchi, and R. R. Netz. 2020. Negative friction memory induces persistent motion. *Eur. Phys. J. E Soft Matter.* 43:67.
45. Jeanneret, R., M. Contino, and M. Polin. 2016. A brief introduction to the model microswimmer Chlamydomonas reinhardtii. *Eur. Phys. J. Spec. Top.* 225:2141–2156. https://doi.org/10.1140/epjst/e2016-60065-3.
46. Nguyen, T. H., M. Kandel, …, G. Popescu. 2017. Halo-free phase contrast microscopy. *Sci. Rep.* 7:44034.
47. Dutcher, S. K., and E. T. O'Toole. 2016. The basal bodies of Chlamydomonas reinhardtii. *Cilia.* 5:18.
48. Wan, K. Y., and R. E. Goldstein. 2016. Coordinated beating of algal flagella is mediated by basal coupling. *Proc. Natl. Acad. Sci. USA.* 113:E2784–E2793.
49. Wakabayashi, K.-i., T. Ide, and R. Kamiya. 2009. Calcium-dependent flagellar motility activation in Chlamydomonas reinhardtii in response to mechanical agitation. *Cell Motil Cytoskeleton.* 66:736–742. https://doi.org/10.1002/cm.20402.
50. Mondal, D., R. Adhikari, and P. Sharma. 2020. Internal friction controls active ciliary oscillations near the instability threshold. *Sci. Adv.* 6, eabb0503. https://doi.org/10.1126/sciadv.abb0503.
51. Mondal, D., A. G. Prabhune, …, P. Sharma. 2021. Strong confinement of active microalgae leads to inversion of vortex flow and enhanced mixing. *Elife.* 10, e67663. https://doi.org/10.7554/eLife.67663.
52. Goldstein, R. E. 2015. Green Algae as Model Organisms for Biological Fluid Dynamics. *Annu. Rev. Fluid Mech.* 47:343–375. https://doi.org/10.1146/annurev-fluid-010313-141426.
53. Kreis, C. T., M. Le Blay, …, O. Bäumchen. 2018. Adhesion of Chlamydomonas microalgae to surfaces is switchable by light. *Nat. Phys.* 14:45–49. https://doi.org/10.1038/nphys4258.
54. Blair, D., and E. Dufresne. 2008. The Matlab Particle Tracking Code Repository. Particle-tracking code. http://site.physics.georgetown.edu/matlab/code.html.
55. Pelleg, D., and A. W. Moore. 2000. X-means: Extending k-means with efficient estimation of the number of clusters. *Icml.* 1:727–734.
56. Bock, H.-H. 2007. Clustering methods: a history of k-means algorithms. *In* Selected Contributions in Data Analysis and Classification, pp. 161–172.
57. Beheshti, S., and M. A. Dahleh. 2005. A new information-theoretic approach to signal denoising and best basis selection. *IEEE Trans. Signal Process.* 53:3613–3624.
58. Shahbaba, M., and S. Beheshti. 2012. Improving x-means clustering with mndl. *In* 2012 11th International Conference on Information Science, Signal Processing and Their Applications (ISSPA) IEEE, pp. 1298–1302.
59. Novikov, A. 2019. PyClustering: Data Mining Library. *J. Open Source Softw.* 4:1230. https://doi.org/10.21105/joss.01230.
60. Celebi, M. E., H. A. Kingravi, and P. A. Vela. 2013. A comparative study of efficient initialization methods for the k-means clustering algorithm. *Expert Syst. Appl.* 40:200–210.
61. Brünig, F. N., O. Geburtig, …, R. R. Netz. 2022. Time-dependent friction effects on vibrational infrared frequencies and line shapes of liquid water. *J. Phys. Chem. B.* 126:1579–1589.
62. Netz, R. R. 2018. Fluctuation-dissipation relation and stationary distribution of an exactly solvable many-particle model for active biomatter far from equilibrium. *J. Chem. Phys.* 148, 185101.
63. Netz, R. R. 2023. Multi-point distribution for Gaussian non-equilibrium non-Markovian observables. https://arxiv.org/abs/2310.08886.
64. Metzler, R., J.-H. Jeon, …, E. Barkai. 2014. Anomalous diffusion models and their properties: non-stationarity, non-ergodicity, and ageing at the centenary of single particle tracking. *Phys. Chem. Chem. Phys.* 16:24128–24164.
65. Friedrich, B. M., and F. Jülicher. 2012. Flagellar synchronization independent of hydrodynamic interactions. *Phys. Rev. Lett.* 109, 138102.
66. Quaranta, G., M.-E. Aubin-Tam, and D. Tam. 2015. Hydrodynamics versus intracellular coupling in the synchronization of eukaryotic flagella. *Phys. Rev. Lett.* 115, 238101.
67. Wang, B. X., C. M. Wu, and K. Ribbeck. 2021. Home, sweet home: how mucus accommodates our microbiota. *FEBS J.* 288:1789–1799.

Biophysical Journal, Volume *123*

Supplemental information

Data-driven classification of individual cells by their non-Markovian motion

Anton Klimek, Debasmita Mondal, Stephan Block, Prerna Sharma, and Roland R. Netz

Supplementary information: Data-driven classification of single cells by their non-Markovian motion

Contents

I Video captions **2**

II Effective kernel follows uniquely from correlation functions **2**

III Green's function is given in terms of positional two-point correlation function **2**

IV Long-time MSDs **4**

V Relation between the oscillation periods of the friction kernel and the VACF **5**

VI Derivation of the analytical expression for the MSD **6**

VII Localization noise fit **9**

VIII Cluster analysis in lower dimensions **10**

IX Comparing friction kernel expressions eq. (29) **and eq.** (19) **10**

X Exemplary non-equilibrium model describing CR motion **11**

XI Information on the cell orientation is contained in the cell-center trajectories **13**

XII Effects of smoothing trajectory data **14**

XIII Markovian embedding of the friction kernel eq. (19) **16**

I Video captions

Video 1.: A representative synchro. High-speed video microscopy at 500 frames per second obtained by phase-contrast imaging of a synchro CR cell showing the planar and synchronous breaststroke motion of the flagella. Between t $\sim 330 - 390\,ms$ the synchronous beat of the flagella exhibits a phase slip, meaning the synchronicity of the flagella is disturbed in that short time interval.
Video 2.: A representative wobbler. High-speed video microscopy at 500 frames per second obtained by phase-contrast imaging of a CR cell which paddles the flagella in an asynchronous and irregular manner resulting in the wobbling motion of the cell body.

II Effective kernel follows uniquely from correlation functions

We can write the GLE with an arbitrary potential $U(x(t))$ as

$$\ddot{x}(t) = -\nabla U(x(t)) - \int_{t_0}^{t} \Gamma_v(t-t')\dot{x}(t')dt' + F_R(t)\,. \tag{S1}$$

Multiplying eq. (S1) by $v(t_0)$, inserting $\Gamma_v(t) = \Gamma(t)$ and averaging the entire equation leads to the Volterra equation

$$\frac{d}{dt}C_{vv}(t) = -C_{v\nabla U}(t) - \int_{0}^{t} \Gamma(t-s)C_{vv}(s)ds\,, \tag{S2}$$

using that the random force is not correlated with the initial velocity at the projection time, i.e. $\langle v(t_0)F_R(t)\rangle = 0$ and setting $t_0 = 0$. Volterra equations are well-behaved and for given functions $\Gamma(t)$ and $C_{v\nabla U}(t)$ one can find a solution of eq. (S2) in terms of $C_{vv}(t)$. Inversely, for given correlation functions $C_{vv}(t)$ and $C_{v\nabla U}(t)$ one can solve eq. (S2) in terms of the friction kernel $\Gamma(t)$ by Laplace transformation. With the definition of the Laplace transform of a function $f(t)$

$$\hat{f}(q) = \int_{0}^{\infty} f(t)e^{-qt}dt, \tag{S3}$$

the unique solution for $\Gamma(t)$ in Laplace space is given by

$$\hat{\Gamma}(q) = -\frac{1}{\hat{C}_{vv}(q)}\left(q\hat{C}_{vv}(q) - C_{vv}(0) + \hat{C}_{v\nabla U}(q)\right)\,. \tag{S4}$$

The existence of a unique friction kernel for any given input correlation functions $C_{vv}(t)$ and $C_{v\nabla U}(t)$ assures that one can always find an effective friction kernel $\Gamma(t)$ that, when employed in the GLE, reproduces the two-point correlation functions. Thus, every non-equilibrium model described by eq. (23) with $\Gamma_R(t) \neq \Gamma_v(|t|)$ can be mapped on an effective model with $\Gamma(|t|) = \Gamma_R(t) = \Gamma_v(|t|)$, because the Green's function, which completely describes a Gaussian process, is solely given in terms of the positional two-point correlation function, see SI Sec. III.

III Green's function is given in terms of positional two-point correlation function

In the following we map a non-equilibrium process described by the GLE eq. (S1) with the random force fluctuations given by eq. (24) and a harmonic potential $U(x) = Kx^2/2$ on an effective model with an effective friction kernel $\Gamma(t)$ fulfilling $\langle F_R(0)F_R(t)\rangle = k_BT\Gamma(|t|)$ and an effective harmonic potential $U_{\text{eff}}(x) = K_{\text{eff}}x^2/2$. For this we show that the Green's function for the non-equilibrium system eqs. (S1), (24) is given in terms of the positional autocorrelation function.

We define the average over the random force by the path integral

$$\langle X\rangle = \mathcal{N}\int \mathcal{D}\tilde{F}_R(\cdot)X\exp\left(-\frac{1}{4\pi B}\int_{-\infty}^{\infty}d\omega\int_{-\infty}^{\infty}d\omega'\frac{\tilde{F}_R(\omega)\delta(\omega+\omega')\tilde{F}_R(\omega')}{\tilde{\Gamma}_R(\omega)}\right)\,. \tag{S5}$$

We also define the generating functional as

$$\begin{aligned}\mathcal{Z}[\tilde{h}] =& \mathcal{N}\int \mathcal{D}\tilde{F}_R(\cdot)\exp\left(-\frac{1}{4\pi B}\int_{-\infty}^{\infty}d\omega\frac{\tilde{F}_R(\omega)\tilde{F}_R(-\omega)}{\tilde{\Gamma}_R(\omega)} + \int_{-\infty}^{\infty}d\omega\tilde{h}(-\omega)\tilde{F}_R(\omega)\right)\\ =& \exp\left(2\pi B\int_{-\infty}^{\infty}d\omega\tilde{h}(\omega)\tilde{h}(-\omega)\tilde{\Gamma}_R(\omega)\right)\end{aligned} \tag{S6}$$

where $\tilde{h}(\omega)$ is a generating field. Equation (S6) has the functional derivatives

$$\frac{\delta \mathcal{Z}[\tilde{h}]}{\delta \tilde{h}(\omega)} \,|_{\tilde{h}=0} = \langle \tilde{F}_R(\omega) \rangle = 0 \tag{S7}$$

$$\frac{\delta^2 \mathcal{Z}[\tilde{h}]}{\delta \tilde{h}(\omega) \delta \tilde{h}(\omega')} \,|_{\tilde{h}=0} = \langle \tilde{F}_R(\omega) \tilde{F}_R(\omega') \rangle = 2\pi B \delta(\omega + \omega') \tilde{\Gamma}_R(\omega) \,, \tag{S8}$$

where $\tilde{\Gamma}_R(\omega) = \tilde{\Gamma}_R(-\omega)$. For harmonic potential $U(x) = Kx^2/2$ we can Fourier-transform the GLE eq. (S1) by which we obtain the form of eq. (S28) with the response function given by

$$\tilde{\chi}(\omega) = \left(K - \omega^2 + i\omega \tilde{\Gamma}_v^+(\omega) \right)^{-1} . \tag{S9}$$

The Green's function can be written as

$$G(x, t \mid x', t') = \frac{P(x, t; x', t')}{P(x', t')}, \tag{S10}$$

which is the ratio of the joint two-point probability distribution to find x at t and x' at t' and the base probability to find x' at t'. Those probabilities can be expressed as

$$P(x', t') = \langle \delta(x' - x(t')) \rangle \,, \; P(x, t; x', t') = \langle \delta(x' - x(t')) \delta(x - x(t)) \rangle \,. \tag{S11}$$

From eq. (S28) we can write the solution of the GLE eq. (S1) using the linear response function $\chi(t)$ as

$$x(t) = \int_{-\infty}^{\infty} \frac{d\omega}{2\pi} e^{i\omega t} \tilde{\chi}(\omega) \tilde{F}_R(\omega). \tag{S12}$$

Inserting this result into the probability distributions eq. (S11) we obtain

$$P(x', t') = \int_{-\infty}^{\infty} \frac{dp}{2\pi} e^{-ipx'} \left\langle \exp\left(ip \int_{-\infty}^{\infty} \frac{d\omega}{2\pi} \tilde{\chi}(\omega) \tilde{F}_R(\omega) e^{i\omega t'} \right) \right\rangle \tag{S13}$$

and

$$P(x, t; x', t') = \int_{-\infty}^{\infty} \frac{dp}{2\pi} \int_{-\infty}^{\infty} \frac{dq}{2\pi} e^{-iqx - ipx'} \Bigg\langle \exp\Bigg(iq \int_{-\infty}^{\infty} \frac{d\omega}{2\pi} \tilde{\chi}(\omega) \tilde{F}_R(\omega) e^{i\omega t} + ip \int_{-\infty}^{\infty} \frac{d\omega}{2\pi} \tilde{\chi}(\omega) \tilde{F}_R(\omega) e^{i\omega t'} \Bigg) \Bigg\rangle. \tag{S14}$$

By comparing eqs. (S13), (S14) to eq. (S6) we find the generating field $\tilde{h}(\omega)$ for the base probability $P(x', t')$

$$\tilde{h}_b(-\omega) = \frac{ip}{2\pi} \tilde{\chi}(\omega) e^{i\omega t'} \tag{S15}$$

and for the joint probability $P(x, t; x', t')$ we find

$$\tilde{h}_j(-\omega) = \frac{i}{2\pi} \tilde{\chi}(\omega) \left(q e^{i\omega t} + p e^{i\omega t'} \right) , \tag{S16}$$

where the subscript b stands for the base probability and j for the joint probability. Using that the position correlation function can be written as

$$C_{xx}(t - t') = B \int_{-\infty}^{\infty} \frac{d\omega}{2\pi} \tilde{\chi}(\omega) \tilde{\Gamma}_R(\omega) \tilde{\chi}(-\omega) e^{i\omega(t-t')}, \tag{S17}$$

we arrive at the Gaussian form

$$\begin{aligned} P(x', t') &= \int_{-\infty}^{\infty} \frac{dp}{2\pi} \exp\left(-C_{xx}(0) p^2 - ipx' \right) \\ &= \sqrt{\frac{1}{2\pi C_{xx}(0)}} \exp\left(-\frac{x'^2}{2C_{xx}(0)} \right) \end{aligned} \tag{S18}$$

and

$$P(x,t;x',0) = \int_{-\infty}^{\infty} \frac{dp}{2\pi} \int_{-\infty}^{\infty} \frac{dq}{2\pi} \exp\left(-\frac{q^2+p^2}{2} C_{xx}(0) - qpC_{xx}(t) - iqx - ipx'\right)$$
$$= 2\sqrt{\frac{1}{2(C_{xx}(0)^2 - C_{xx}(t)^2)}} \exp\left(-\frac{(x'+x)^2}{4(C_{xx}(0)+C_{xx}(t))} - \frac{(x'-x)^2}{4(C_{xx}(0)-C_{xx}(t))}\right). \tag{S19}$$

For simplicity we set $t' = 0$ as the position correlation function only depends on the time difference $t - t'$. Inserting eqs. (S18) (S19) into eq. (S10), we finally obtain the position Green's function as a Gaussian

$$G(x,t \mid x',0) = \frac{\exp(-\frac{(x-\mu)^2}{2\sigma^2})}{\sqrt{2\pi\sigma^2}} \tag{S20}$$

with mean and standard deviation

$$\mu = \mu(x',t) = \frac{C_{xx}(t)}{C_{xx}(0)} x' \,, \; \sigma^2 = \sigma^2(t) = C_{xx}(0)\left(1 - \frac{C_{xx}(t)^2}{C_{xx}(0)^2}\right). \tag{S21}$$

Thus the Green's function is entirely described in terms of the two-point positional correlation function $C_{xx}(t)$, note that $C_{vv}(t) = -\frac{d^2}{dt^2} C_{xx}(t)$.

We next calculate the effective harmonic potential strength K_{eff}. The condition to find an effective description of the GLE eq. (S1) arises from setting eq. (S17) equal to its effective version

$$\tilde{\chi}(\omega)\tilde{\Gamma}_R(\omega)\tilde{\chi}(-\omega) = \tilde{\chi}_{\text{eff}}(\omega)\tilde{\Gamma}(\omega)\tilde{\chi}_{\text{eff}}(-\omega)\,, \tag{S22}$$

which assures that the effective model has the same Green's function as the non-equilibrium model, as shown by eqs. (S20) and (S21). Here, $\tilde{\chi}_{\text{eff}}(\omega)$ is the effective response function and $\Gamma(t)$ is the effective kernel. Inserting eq. (S9) into eq. (S22) leads to

$$\frac{\tilde{\Gamma}_R(\omega)}{(K - \omega^2 + i\omega\tilde{\Gamma}_v^+(\omega))(K - \omega^2 - i\omega\tilde{\Gamma}_v^+(-\omega))} = \frac{\tilde{\Gamma}(\omega)}{(K_{\text{eff}} - \omega^2 + i\omega\tilde{\Gamma}^+(\omega))(K_{\text{eff}} - \omega^2 - i\omega\tilde{\Gamma}^+(-\omega))}. \tag{S23}$$

Defining the potential of mean force for the effective system according to

$$U_{\text{eff}}(x) = -B\ln(p(x)) = K_{\text{eff}} x^2/2, \tag{S24}$$

with $p(x)$ being the probability to observe x, and comparing eq. (S24) to the long time limit of the non-equilibrium distribution eq. (S20), namely

$$p(x) = \lim_{t\to\infty} G(x,t \mid x',0) = \frac{\exp(-\frac{x^2}{2C_{xx}(0)})}{\sqrt{2\pi C_{xx}(0)}}\,, \tag{S25}$$

we find the effective harmonic potential strength to be

$$K_{\text{eff}} = \frac{B}{C_{xx}(0)}\,. \tag{S26}$$

Note that the effective potential will in general deviate from the potential in the non-equilibrium GLE, i.e. $K_{\text{eff}} \neq K$. The present calculation can also be done for a general multi-point distribution (63).

IV Long-time MSDs

The trajectories analyzed in the main text (examples shown in Figs. 2a,b) are recorded with a 40 fold magnification at 500 frames per second, which leads to trajectories of lengths up to $14\,\text{s}$ (Fig. 2h). Here, we analyze a different data set to resolve the long time behavior of the MSD of the CR cells, which we obtain by using a lower magnification (tenfold) and longer time step of $0.02\,\text{s}$. A lower magnification results in a larger observation window, which in turn allows recording longer trajectories. The decreased localization precision and the longer time step do not allow to observe the details of the oscillatory features of the motion. Hence, we do not apply the cluster analysis relying on the extraction of the oscillating friction kernels,

but rather use an approximate distinction by the mean squared velocities B. We assign cells with $B > 110\,\mu\mathrm{m}^2/\mathrm{s}^2$ to be putative wobblers and cells with $B < 35\,\mu\mathrm{m}^2/\mathrm{s}^2$ to be putative synchros. In this way, we avoid the assignment of cells with intermediate mean squared velocities B, for which this approximate classification scheme is unreliable. For the slow cells we additionally exclude cells for which the MSD stays below $1\,\mu\mathrm{m}^2$ during the entire trajectory. These cells are most likely stuck or have some defects prohibiting them to move. This distinction into putative wobblers and synchros depends on our choice of the upper and lower bounds for B, which we choose according to the high resolution fastest synchro and slowest wobbler when using only every tenth step of the high resolution data, by which we obtain velocities with a time step of $0.02\,\mathrm{s}$. In Fig. S1 we demonstrate that the present approximate classification leads to extended MSDs (black lines) that match closely the MSDs shown in the main text based on the high-resolution videos (gray lines). The extended MSDs for synchros exhibit a transition from the ballistic to the long time diffusive regime at a time of about $t \approx 2$s, whereas for the wobblers the ballistic regime extends over more than ten seconds. The prefactor of the MSD in the diffusive regimes, i.e. the diffusivity D, is for the synchros in Fig. S1 given by $D = 9\,\mu\mathrm{m}^2/\mathrm{s}$. This transition to the long time diffusive regime is also indicated by the exemplary trajectories in Fig. S1c, where the synchro shows no directed motion on the time scale of the entire trajectory whereas the wobbler trajectory is still rather directed over the entire trajectory. Moreover, the higher speed of the wobblers is again reflected in higher MSDs for wobblers, as demonstrated in Fig. S1d.

V Relation between the oscillation periods of the friction kernel and the VACF

In Fig. 4 in the main text one can note a subtle difference between the oscillation period of the friction kernel and the oscillation period of the VACF when comparing Fig. 4b to 4c or Fig. 4e to 4f. Since we derive an analytical expression for the VACF in eq. (S52) for the friction kernel given in eq. (29), we can evaluate the dependence of the VACF oscillation frequency on the kernel frequency Ω. We are interested in the dominant oscillatory behavior at short times, therefore we consider the pole ω_i from eq. (S52) with the smallest real part, where we call this real part ω_{vv}. In Fig. S2a we show the dependence of ω_{vv} on Ω for different b and find the VACF frequency ω_{vv} to be constant for small kernel frequencies Ω followed by a transition to a regime where ω_{vv} equals Ω above a threshold value given by $\Omega_t = \omega_{vv}(\Omega \to 0)$. This threshold value depends on the kernel parameters a, b, τ and has the asymptotic behavior

$$\Omega_t = \begin{cases} \sqrt{b} & \text{, for } b > \max(a^2/4, \tau^{-2}/4) \\ a/2 & \text{, for } b < a^2/4 \wedge \tau > a^{-1} \\ \tau^{-1}/2 & \text{, for } b < a^2/4 \wedge \tau < a^{-1} \,, \end{cases} \tag{S27}$$

which follows from Figs. S2a-c.

Since the median kernel frequency of the CR cells is of the order $\Omega \sim 100\,\tau^{-1}$ and the oscillation amplitude is of the order $b \sim 10^4\tau^{-2}$, the data lies in the transition regime between constant VACF frequency ω_{vv} and the linear regime $\omega_{vv} = \Omega$, as can be seen in Fig. S2a. From Fig. S2a we infer that the VACF frequency is always greater or equal to the kernel frequency, i.e. $\omega_{vv} \geq \Omega$. Moreover, the oscillation amplitudes of the CR cells usually fulfill $b > a^2/4$ and the inverse decay time τ^{-1} is small compared to the other kernel parameters, such that $a > \tau^{-1}$ and $b > \tau^{-2}/4$ are always fulfilled. Therefore, the cells exhibit parameters for which $b > \max(a^2/4, \tau^{-2}/4)$ holds. Eq. (S27) thus tells us that the threshold frequency is given by $\Omega_t = \sqrt{b}$. Combining this knowledge with $\omega_{vv} \geq \Omega$ leads to $\omega_{vv} \geq \sqrt{b}$. Since the CR data lies in the transition regime between $\omega_{vv} = \sqrt{b}$ and $\omega_{vv} = \Omega$, the VACF frequency ω_{vv} is strongly influenced by the oscillation amplitude b. In order to further estimate the exhibited parameter range, the reader is referred to Fig. 5 of the main text, which includes all parameters of all cells.

The fact that the VACF frequency ω_{vv} increases with increasing kernel oscillation amplitude b can be interpreted in the context of the Markovian embedding model in Sec. XIII, where the coupling strength of the harmonically coupled reaction coordinates is proportional to the oscillation amplitude b, as shown in eqs. (S75), (S77), which leads to faster oscillations for higher b. Since every oscillation leads to a net forward motion of the cell, it leads to fast net forward motion for cells if they exhibit a large kernel oscillation amplitude b compared to the squared inverse decay time τ^{-2} and the squared δ-amplitude a^2, which is the case in our data set (see Fig. 5). By realizing that the decay time τ is related to how long a cell 'remembers' its past trajectory, one can conclude that a fast decay of the oscillation would lead to a cell not being able to maintain its direction for a long time. Thus, it is useful to achieve fast net forward motion if the decay time is longer than the oscillation period, i.e. if $\tau\Omega > 1$. By net forward motion we mean directed motion on relevant time scales, since of course in the long-time limit the cells exhibit diffusion as shown in Fig. S1. Both criteria, the oscillation amplitude being large, i.e. $b > \max(\tau^{-2}, a^2)$ and the decay time being long compared to the oscillation period, i.e. $\tau\Omega > 1$, are fulfilled by the CR cell parameters shown in Fig. 5, which means that CR cells exhibit a range of kernel parameters that is suited to achieve fast net forward motion.

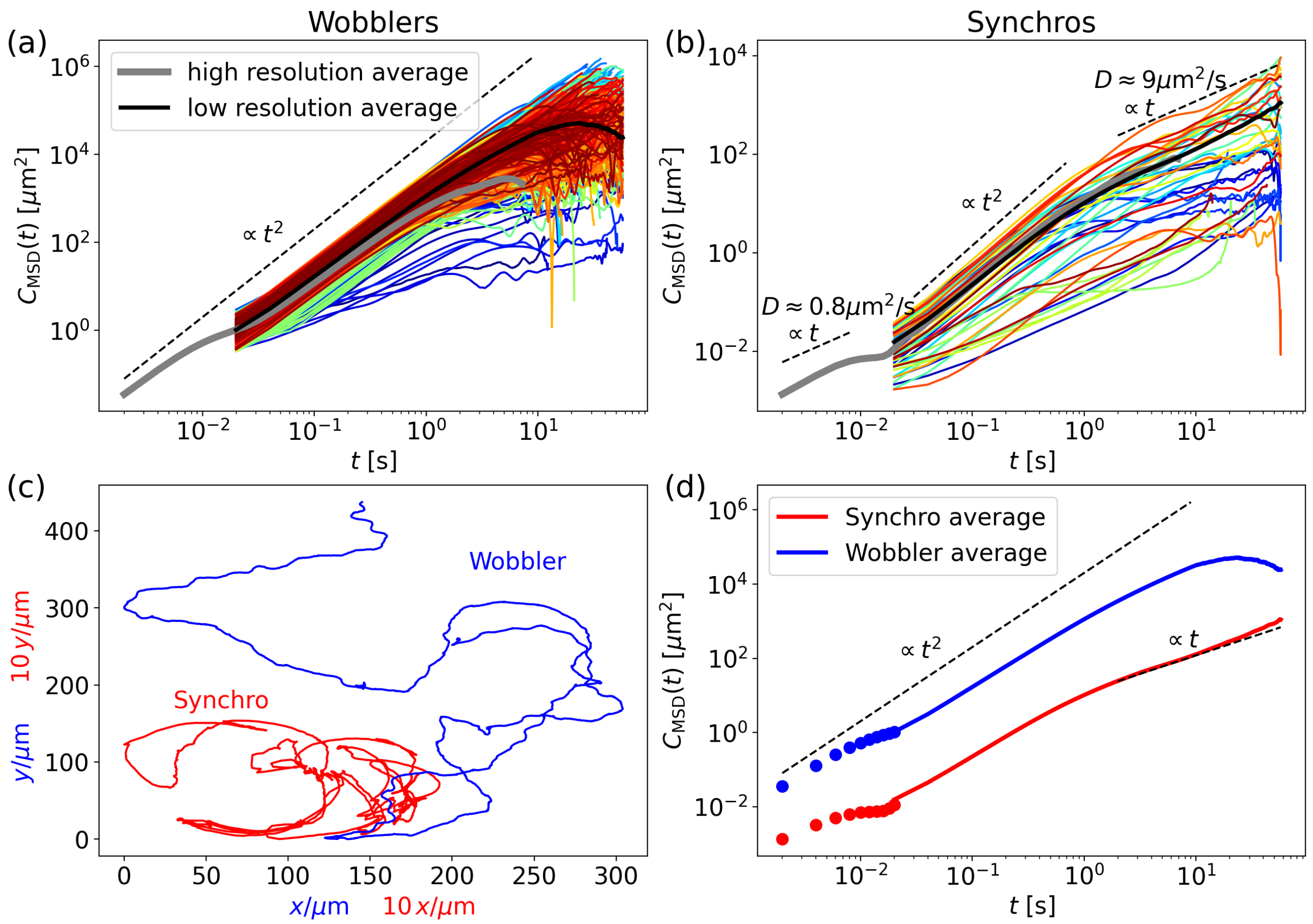

Figure S1: MSDs of individual cells (colored lines) from low-resolution data with 10x magnification and a time step of $\Delta = 0.02\,\mathrm{s}$ of (a) wobblers and (b) synchros according to an approximate classification scheme explained in the text. The average of the low-resolution MSD data is shown in black, the grey line represents the average of the MSD from Fig. 3 in the main text with higher temporal and spatial resolution of 40x and $\Delta = 0.002\,\mathrm{s}$. (c) Representative trajectories of the low resolution data of a wobbler in blue of length $57.1\,\mathrm{s}$ and of a synchro in red of length $50.9\,\mathrm{s}$, where the synchro positions are multiplied by a factor of ten to show them on the same scale with the wobbler trajectory. (d) The concatenated average MSD of short times from high resolution data as points and long times from low resolution data as solid lines for synchros in red and wobblers in blue.

VI Derivation of the analytical expression for the MSD

In Fourier space the GLE eq. (23) can be written as

$$\tilde{x}(\omega) = \tilde{\chi}(\omega)\tilde{F}_R(\omega)\,, \tag{S28}$$

where $\tilde{\chi}(\omega)$ is the Fourier transform of the position response function, which takes the form

$$\tilde{\chi}(\omega) = \left(-\omega^2 + i\omega\tilde{\Gamma}_v^+(\omega)\right)^{-1} \tag{S29}$$

with $\tilde{\Gamma}_v^+(\omega)$ being the half sided Fourier transform of the friction kernel defined via

$$\tilde{\Gamma}_v^+(\omega) = \int_0^\infty e^{-i\omega t}\Gamma_v(t)dt\,. \tag{S30}$$

The Fourier transform of the position correlation function $C_{xx}(t) = \langle x(0)x(t)\rangle$ can be written as

$$\tilde{C}_{xx}(\omega) = B\tilde{\chi}(\omega)\tilde{\Gamma}_R(\omega)\tilde{\chi}(-\omega)\,, \tag{S31}$$

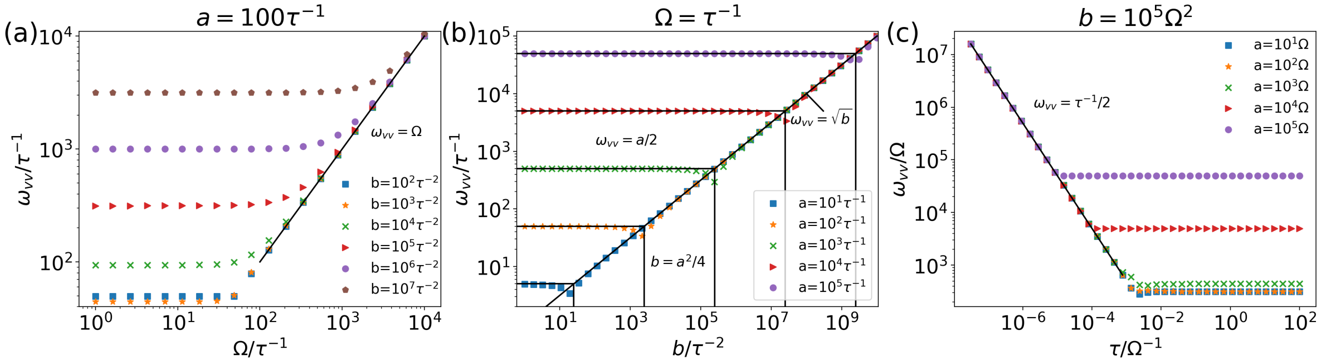

Figure S2: (a) The dependence of the VACF frequency ω_{vv} on the kernel frequency Ω of the kernel eq. (29) is shown for different kernel oscillation amplitudes b and fixed δ-peak amplitude $a = 100\tau^{-1}$. The black line indicates the linear behavior $\omega_{vv} = \Omega$. (b) The dependence of the VACF frequency ω_{vv} on the kernel oscillation amplitude b is shown for varying kernel δ-peak amplitude a and $\Omega = \tau^{-1}$, which represents the low kernel frequency plateau value shown in (a) for small Ω. The horizontal lines represent $\omega_{vv} = a/2$ for the respective values of a, the diagonal line represents $\omega_{vv} = \sqrt{b}$ and the vertical lines represent the intersection of the other lines, which mark a minimum in the parameter b positioned at $b = a^2/4$. (c) The dependence of the VACF frequency ω_{vv} on the kernel decay time τ shown for different δ-peak amplitudes a and fixed $b = 10^5\Omega^2$. The regime shown for τ/Ω^{-1} represents the low kernel frequency plateau value shown in (a) for small $\Omega\tau < 100$. The black line indicates the scaling $\omega_{vv} = \tau^{-1}/2$.

where we made use of

$$\begin{aligned}\tilde{C}_{xx}(\omega) &= \int_{-\infty}^{\infty} dt e^{-i\omega t}\langle x(0)x(t)\rangle \\ &= \int_{-\infty}^{\infty} e^{-i\omega t}dt \int_{\infty}^{\infty} e^{i\omega t}\frac{d\omega}{2\pi}\int_{-\infty}^{\infty}\frac{d\omega'}{2\pi}\langle\tilde{x}(\omega)\tilde{x}(\omega')\rangle\end{aligned} \tag{S32}$$

and

$$\langle \tilde{F}_R(\omega)\tilde{F}_R(\omega')\rangle = 2\pi B\delta(\omega+\omega')\tilde{\Gamma}_R(\omega')\,. \tag{S33}$$

Equation S31 can be rewritten as

$$\tilde{C}_{xx}(\omega) = \frac{B}{i\omega}\left(\tilde{\chi}(\omega) - \tilde{\chi}(-\omega)\right)\frac{\tilde{\Gamma}_R(\omega)}{\tilde{\Gamma}_v(\omega)}\,, \tag{S34}$$

which leads to the MSD

$$\begin{aligned}C_{\mathrm{MSD}}(t) &= 2(C_{xx}(0) - C_{xx}(t)) \\ &= B\int_{-\infty}^{\infty}\frac{d\omega}{\pi}\frac{e^{i\omega t}-1}{i\omega}\left(\tilde{\chi}(\omega)-\tilde{\chi}(-\omega)\right)\frac{\tilde{\Gamma}_R(\omega)}{\tilde{\Gamma}_v(\omega)}\,.\end{aligned} \tag{S35}$$

For the decaying oscillation model friction kernel of eq. (29) and using the effective form $\Gamma(|t|) = \Gamma_R(t) = \Gamma_v(|t|)$ one obtains the half-sided Fourier transform of the kernel as

$$\tilde{\Gamma}^{+}(\omega) = \int_0^{\infty}\left(2a\delta(t) + be^{-t/\tau}\cos(\Omega t)\right)e^{-i\omega t}dt = a + b\left(\frac{i\omega+\frac{1}{\tau}}{\Omega^2+(i\omega+\frac{1}{\tau})^2}\right)\,. \tag{S36}$$

Inserting the result of eq. (S36) into eq. (S29) we find the response function

$$\tilde{\chi}(\omega) = \left(-\omega^2 + i\omega a + i\omega b\left(\frac{i\omega+\frac{1}{\tau}}{\Omega^2+(i\omega+\frac{1}{\tau})^2}\right)\right)^{-1}\,, \tag{S37}$$

which by inserting into eq. (S35) leads to

$$C_{\mathrm{MSD}}(t) = B\int_{-\infty}^{\infty}\frac{d\omega}{\pi}\frac{(e^{i\omega t}-1)(k_1+k_2\omega^2+k_3\omega^4)}{\omega^2(c_1+c_2\omega^2+c_3\omega^4+\tau^4\omega^6)}\,, \tag{S38}$$

where the constants c_i and k_i are given by

$$\begin{aligned} c_1 &= (a + b\tau + a\Omega^2\tau^2)^2 && \text{(S39)} \\ c_2 &= (1 + 2(a^2 - b + \Omega^2)\tau^2 + 2ab\tau^3 + (-2a^2\Omega^2 + (b + \Omega^2)^2)\tau^4) && \text{(S40)} \\ c_3 &= \tau^2(2 + (a^2 - 2(b + \Omega^2))\tau^2) && \text{(S41)} \\ k_1 &= -2(1 + \Omega^2\tau^2)(a + b\tau + a\Omega^2\tau^2) && \text{(S42)} \\ k_2 &= -2\tau^2(b\tau + a(2 - 2\Omega^2\tau^2)) && \text{(S43)} \\ k_3 &= -2a\tau^4 \,. && \text{(S44)} \end{aligned}$$

Interpreting the integrand in eq. (S38) as a sum of the three terms each proportional to k_i, we see that all terms have the same poles, where the term proportional to k_1 has an additional double pole at $\omega = 0$. The solutions of ω^2 to the equation

$$c_1 + c_2\omega^2 + c_3\omega^4 + \tau^4\omega^6 = \tau^4(\omega^2 - \omega_1^2)(\omega^2 - \omega_2^2)(\omega^2 - \omega_3^2) = 0\,, \tag{S45}$$

define the remaining poles, which we denote by $\pm\sqrt{\omega_i^2}$ and which we compute by numerically solving eq. (S45). Next we use the partial fraction decompositions

$$\begin{aligned} \frac{1}{\omega^2 \prod_{i=1}^3(\omega^2 - \omega_i^2)} &= -\frac{1}{\omega^2 \prod_{i=1}^3 \omega_i^2} + \sum_{i=1}^3 \frac{1}{\omega_i^2(\omega^2 - \omega_i^2)\prod_{j\neq i}(\omega_i^2 - \omega_j^2)} && \text{(S46)} \\ \frac{1}{\prod_{i=1}^3(\omega^2 - \omega_i^2)} &= \sum_{i=1}^3 \frac{1}{(\omega^2 - \omega_i^2)\prod_{j\neq i}(\omega_i^2 - \omega_j^2)} && \text{(S47)} \\ \frac{\omega^2}{\prod_{i=1}^3(\omega^2 - \omega_i^2)} &= \sum_{i=1}^3 \frac{\omega_i^2}{(\omega^2 - \omega_i^2)\prod_{j\neq i}(\omega_i^2 - \omega_j^2)} && \text{(S48)} \end{aligned}$$

to rewrite the fraction of eq. (S38) as a sum of terms proportional to $(\omega^2 - \omega_i^2)^{-1}$ and one term proportional to ω^{-2}. Using the solution of the integrals

$$\begin{aligned} \int_{-\infty}^{\infty} \frac{e^{i\omega t} - 1}{\omega^2 - \omega_i^2} d\omega &= \frac{\pi(e^{-\sqrt{-\omega_i^2}t} - 1)}{\sqrt{-\omega_i^2}} && \text{(S49)} \\ \int_{-\infty}^{\infty} \frac{e^{i\omega t} - 1}{\omega^2} d\omega &= -\pi t && \text{(S50)} \end{aligned}$$

for $t > 0$ with the condition $\text{Re}(\omega_i^2) < 0 \vee \text{Im}(\omega_i^2) \neq 0 \wedge \text{Im}(\sqrt{\omega_i^2}) \neq 0$, we can rewrite the integral of the MSD eq. (S38) as

$$C_{\text{MSD}}(t) = \frac{B}{\tau^4}\left(\frac{k_1 t}{\omega_1^2\omega_2^2\omega_3^2} + \sum_{i=1}^3 \frac{e^{-\sqrt{-\omega_i^2}t} - 1}{\sqrt{-\omega_i^2}\prod_{j\neq i}(\omega_i^2 - \omega_j^2)}\left[\frac{k_1}{\omega_i^2} + k_2 + k_3\omega_i^2\right]\right)\,. \tag{S51}$$

The VACF can be computed from the MSD by using eq. (16) as

$$C_{vv}(t) = \frac{B}{2\tau^4}\left(\sum_{i=1}^3 \frac{\sqrt{-\omega_i^2}e^{-\sqrt{-\omega_i^2}t}}{\prod_{j\neq i}(\omega_i^2 - \omega_j^2)}\left[\frac{k_1}{\omega_i^2} + k_2 + k_3\omega_i^2\right]\right)\,. \tag{S52}$$

The calculation of the MSD for the friction kernel of eq. (19) proceeds in the same way as shown for the kernel eq. (29). The response function then takes the slightly different form

$$\tilde{\chi}(\omega) = \left(-\omega^2 + i\omega a + i\omega b\left(\frac{i\omega + \frac{2}{\tau}}{\Omega^2 + (i\omega + \frac{1}{\tau})^2}\right)\right)^{-1}\,. \tag{S53}$$

The integral eq. (S35) resulting in the MSD can be written in the same form as before shown in eq. (S38), where the constants c_1, c_2, k_1, k_2 take different values of

$$\begin{aligned} c_1 &= (a + 2b\tau + a\Omega^2\tau^2)^2 && \text{(S54)} \\ c_2 &= (1 + 2(a^2 - 3b + \Omega^2)\tau^2 + (-2a^2\Omega^2 + (b + \Omega^2)^2)\tau^4) && \text{(S55)} \\ k_1 &= -2(1 + \Omega^2\tau^2)(a + 2b\tau + a\Omega^2\tau^2) && \text{(S56)} \\ k_2 &= 4a\tau^2(\Omega^2\tau^2 - 1)\,. && \text{(S57)} \end{aligned}$$

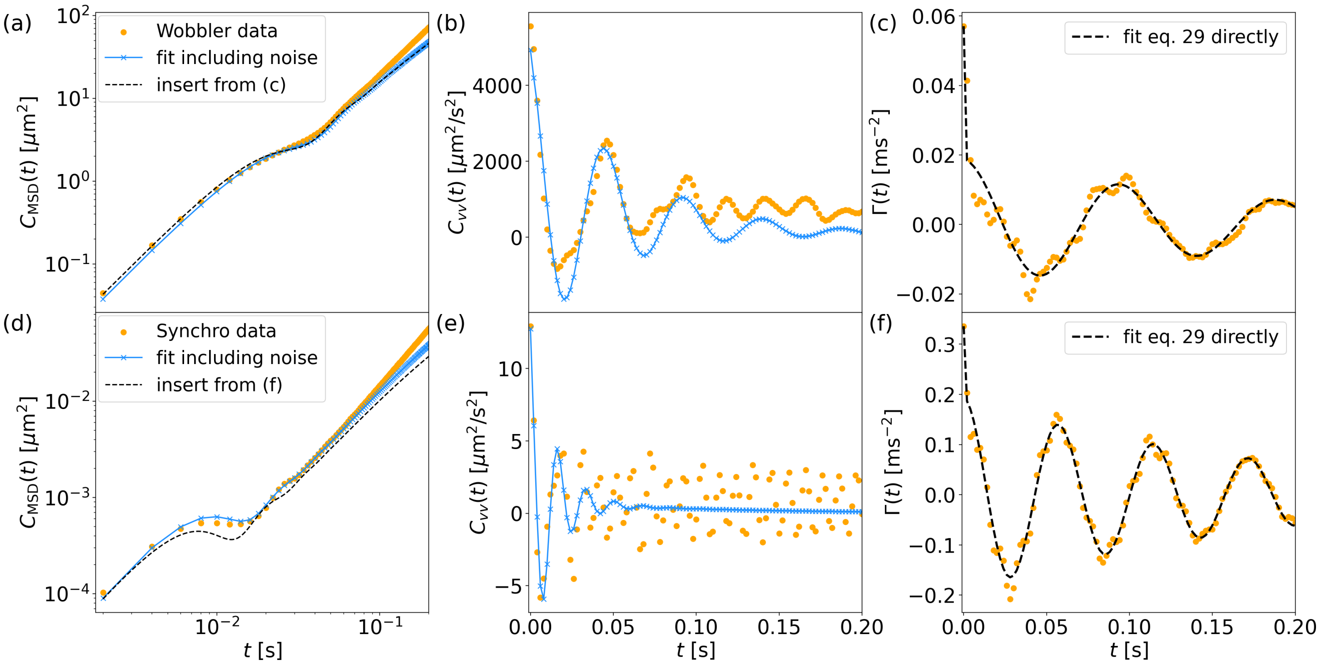

Figure S3: Two example cells for which the localization-noise fit to the VACF does not agree well with the data. The MSD $C_{\mathrm{MSD}}(t)$ (eq. (28)), VACF $C_{vv}(t)$ (eq. (25)) and friction kernel $\Gamma(t)$ extracted by eq. (8) of a single (a)-(c) wobbler and (d)-(f) synchro are displayed as orange dots. Blue crosses in the VACF figures represent the results from the localization noise fit according to eq. (17) and the blue crosses in the MSD figures result from inserting the fit parameters of the VACF into eq. (15). The black dashed line in (c) and (f) is the direct fit of eq. (29) to the extracted kernels, which is used to obtain parameters for the cell classification, see Methods Sec. 2.6. The dashed black lines in the MSD figures result from inserting the fitting result of the friction kernel (dashed lines in (c), (f) respectively) and the mean squared velocity $B = C_{vv}(0)$ into the analytical solution of the MSD eq. (S51).

Thus, the MSD of the GLE eq. (23) with the friction kernel eq. (19) can be described by the same form eq. (S51) as for the friction kernel of eq. (29), where only the constants c_i and k_i have slightly different values as seen by comparing eqs. (S39)-(S44) to eqs. (S54)-(S57).

VII Localization noise fit

The fitting procedure including a finite time step and localization noise described in Sec. 2.7 of the main text does sometimes not converge to results that agree well with the VACF of the data. In Fig. S3 we show two examples for which the fit including the localization noise (Methods eq. (17)) does not agree well with the VACF data. For 14 out of the 59 cells, the fits of the VACF using eq. (18) do not converge to a stable set of parameters. Still the direct fit of the friction kernel model eq. (29) agrees perfectly with the extracted friction kernels, which is why we use this direct fit to extract parameters, that are later used in the cluster analysis.

A typical size of the localization noise width resulting from the fits is $\sigma_{\mathrm{loc}} \approx 0.02\,\mu\mathrm{m}$. Considering the resolution of the microscope of roughly $\sim 0.5\,\mu\mathrm{m}$ and approximating the area of a cell as roughly $25\pi\,\mu\mathrm{m}^2$, one can estimate the number of pixels per cell as 100π. Thus, the error of the mean position of a cell evaluated by all pixels is estimated by $0.5\,\mu\mathrm{m}/\sqrt{100\pi} \approx 0.03\,\mu\mathrm{m}$, which is very close to the fitted localization noise width of $\sigma_{\mathrm{loc}} \approx 0.02\,\mu\mathrm{m}$. For a typical velocity around $v = 100\,\mu\mathrm{m/s}$, the displacement during one time step $\Delta = 0.002\,\mathrm{s}$ is $0.2\,\mu\mathrm{m}$. Therefore, the localization noise accounts for $\sim 10\%$ of the cell displacement. This relatively small localization noise explains why the direct fit of the model eq. (29) to the extracted friction kernels works so well and the inclusion of localization noise effects is not necessary for our data set.

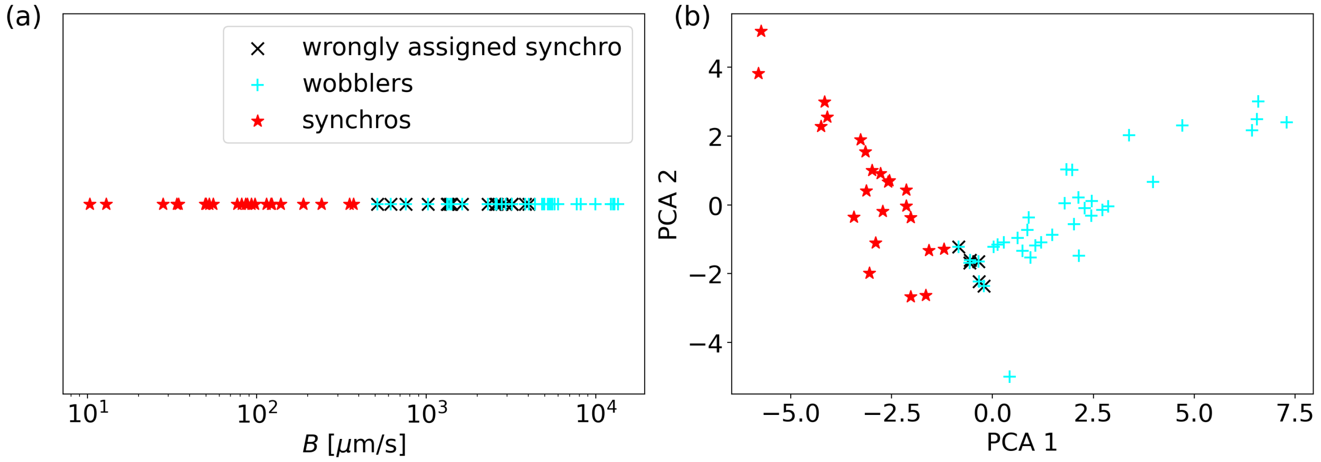

Figure S4: (a) Mean squared velocities B for all cells; a cluster analysis solely based on B leads to the wrong assignment of 18 wobblers as synchros, indicated by black crosses. (b) Projection of the five dimensional extracted parameters shown in Fig. 5 on the first two components of the PCA, which explain $61\,\%$ and $22\,\%$ of the total parameter variance, respectively. The black crosses indicate six wobbler cells that are wrongly assigned as synchros when applying the cluster analysis only to the shown two PCA components.

VIII Cluster analysis in lower dimensions

Since the wobblers are found to exhibit higher average speeds than the synchros, an intuitive and simple approach would be to distinguish the cells solely by their mean squared velocity B. Applying the cluster analysis described in Sec. 2.8 of the Methods in one dimension for B, results in a assignment agreeing only to $69\,\%$ with the full classification result, see Fig. S4a. Especially slow wobblers are wrongly assigned as synchros, as indicated in Fig. S4a by the black crosses.

As discussed in the main text, high-dimensional data sets are often analyzed with principal-component analysis (PCA), which determines the directions explaining most of the data variance. Applying a PCA to the five-dimensional parameter space of the extracted friction kernel parameters eq. (29) and B shown in Fig. 5, we find the first two components of the PCA to explain $83\,\%$ of the total parameter variance. This explanation of variance is not to be confused with the agreement of the clustering method with the assignment by visual inspection of the flagella. Here, we use the PCA tool implemented in *sklearn* for python. The normalized vectors of the shown two PCA components in the order $(a,\ b,\ \tau,\ \Omega,\ B)$ are given by $(-0.49,\ -0.29,\ 0.16,\ -0.01,\ 0.81)$ and $(0.63,\ 0.41,\ -0.5,\ 0.05,\ 0.43)$, respectively. This indicates, that no parameter alone can describe the complete variance and therefore no parameter alone can explain all the differences between synchros and wobblers. The parameters most important for the variance and discriminability of the single CR cells are a, b and B. However, the distinction into the two groups of wobblers and synchros by our cluster analysis (Sec. 2.8 in the Methods) works the best using the complete five dimensional set of parameters. Applying the cluster analysis to the first two PCA components results in an accuracy of $90\,\%$, as shown in Fig. S4b. Six wobblers are assigned to belong to the cluster of synchros (defined by the classification of (51) that agrees with our cluster analysis of the complete parameter set Fig. 5) as they lie in the transition area between the two clusters and are indicated by the black crosses in Fig. S4b.

IX Comparing friction kernel expressions eq. (29) and eq. (19)

The friction kernel of eq. (29) in the main text used to extract parameters from the data is very similar to the friction kernel eq. (19), which is derived in Sec. XIII from a system of harmonically coupled particles. The term $1/(\tau\Omega)$ in front of the sine in eq. (19) becomes small when the decay time τ is longer than the oscillation period $1/\Omega$. Several oscillations occur before the friction kernel has decayed for the CR data in Fig. 3 in the main text and Fig. S6, thus, the term $1/(\tau\Omega)$ is indeed small and the two friction kernel models have very similar shapes, as we show in Fig. S6. Here, we insert the extracted parameters from the friction kernel eq. (29) of two example cells into eq. (19), the deviations are seen to be very small.

10

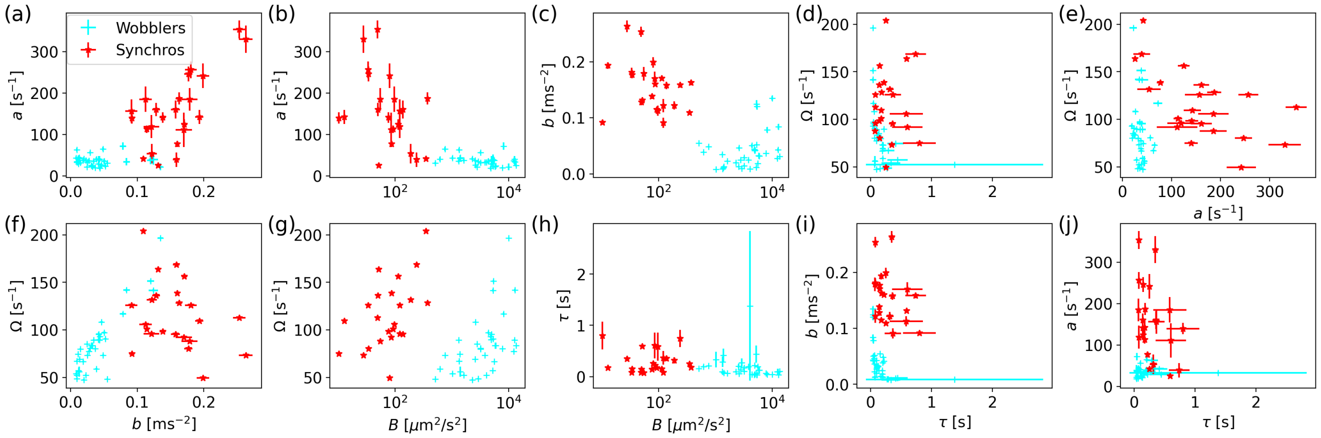

Figure S5: Same figure as in the main text Fig. 5 but with error estimates for the parameters, which we obtain from scipy's curve fit function as the square root of the diagonal elements of the approximate covariance around the optimal fit result. The errors of all parameters are in the range of a few percent, except for the decay time τ, where the highest error is in the same order as the parameter value itself.

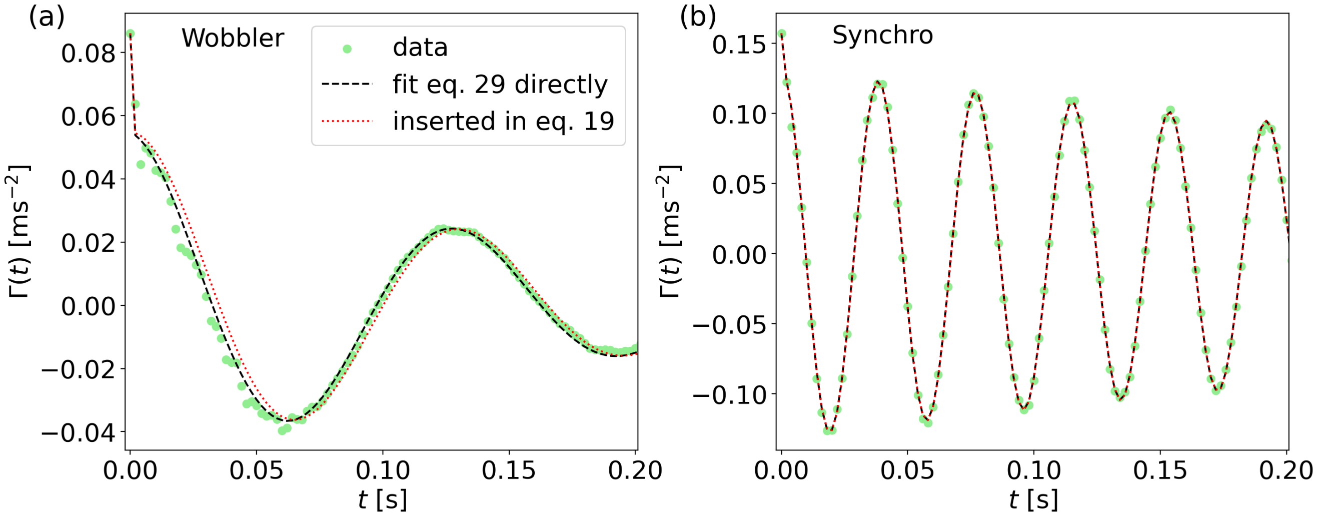

Figure S6: Friction kernel $\Gamma(t)$ extracted by eq. (8) of (a) a single wobbler and (b) single synchro shown as green data points. The black line shows the direct fit of eq. (29) to the data points and the red dotted line results from inserting the fitted parameters from the black dashed line into eq. (19) derived from the Hamiltonian eq. (20) in Sec. XIII. The agreement of the black dashed line and the red dotted line means that in the parameter range exhibited by the CR cells, the two kernels eq. (29) and eq. (19) describe the data equally well.

X Exemplary non-equilibrium model describing CR motion

The effective friction kernel $\Gamma(t)$ given by eq. (19) leads to the Fourier transform

$$\begin{aligned}\tilde{\Gamma}^{+}(\omega) &= \int_0^\infty \left(2a\delta(t) + be^{-t/\tau}\left(\cos(\Omega t) + \frac{1}{\tau\Omega}\sin(\Omega t)\right)\right)e^{-i\omega t}dt \\ &= a + b\left(\frac{i\omega + \frac{2}{\tau}}{\Omega^2 + (i\omega + \frac{1}{\tau})^2}\right) . \qquad \text{(S58)}\end{aligned}$$

Inserting eq. (S58) into eq. (4) yields the Fourier-transformed VACF

$$\begin{aligned}\frac{\tilde{C}_{vv}(\omega)}{B} &= \Big(2a + 2b\tau + 4a\Omega^2\tau^2 + 2b\Omega^2\tau^3 + 2a\Omega^4\tau^4 + 4ib\tau^2\omega \\ &+ (4a\tau^2 + 2b\tau^3 - 4a\Omega^2\tau^4)\omega^2 + 2a\tau^4\omega^4\Big)/ \\ &\Big(a + 2b\tau + a\Omega^2\tau^2)^2 + (1 + 2(a^2 - 3b + \Omega^2)\tau^2 + (-2a^2\Omega^2 + (b + \Omega^2)^2)\tau^4)\omega^2 \\ &+ \tau^2(2 + (a^2 - 2(b + \Omega^2))\tau^2)\omega^4 + \tau^4\omega^6\Big).\end{aligned} \tag{S59}$$

In order to find a non-equilibrium model, that leads to the same VACF as the kernel in eq. (19), we assume a specific form of the velocity friction kernel

$$\Gamma_v(t) = 2a_v\delta(t) \tag{S60}$$

which leads to the Fourier transform $\tilde{\Gamma}_v^+(\omega) = a_v$. We insert this together with the result from eq. (S59) into eq. (5) and solve for $\tilde{\Gamma}_R(\omega)$. By Fourier back transformation the result can be written in the form

$$\Gamma_R(t) = \int_{-\infty}^{\infty} \frac{d\omega}{2\pi} e^{i\omega t} \frac{k_0 + k_1\omega + k_2\omega^2 + k_3\omega^3 + k_4\omega^4 + k_6\omega^6}{c_0 + c_1\omega^2 + c_2\omega^4 + c_3\omega^6} \tag{S61}$$

with the constants c_i and k_i being

$$\begin{aligned}c_0 &= (a + 2b\tau + a\Omega^2\tau^2)^2 \\ c_1 &= 1 + 2(a^2 - 3b + \Omega^2)\tau^2 + (-2a^2\Omega^2 + (b + \Omega^2)^2)\tau^4 \\ c_2 &= \tau^2(2 + (a^2 - 2(b + \Omega^2))\tau^2) \\ c_3 &= \tau^4 \\ k_0 &= 2aa_v^2 + 2a_v^2b\tau + 4aa_v^2\Omega^2\tau^2 + 2a_v^2b\Omega^2\tau^3 + 2aa_v^2\Omega^4\tau^4 \\ k_1 &= 4a_v^2b\tau^2 \\ k_2 &= 2(b\tau(1 + a_v^2\tau^2 + \Omega^2\tau^2) + a((1 + \Omega^2\tau^2)^2 + a_v^2(2\tau^2 - 2\Omega^2\tau^4))) \\ k_3 &= 4b\tau^2 \\ k_4 &= 2\tau^2(b\tau + a(2 + a_v^2\tau^2 - 2\Omega^2\tau^2)) \\ k_6 &= 2a\tau^4\,.\end{aligned}$$

We solve this Fourier transform by performing the same steps as for the calculation of the integral (S38) derived in Sec. VI, where ω_i^2 are the roots of the denominator of eq. (S61) in ω^2, which we obtain numerically, and we additionally use

$$\int_{-\infty}^{\infty} \frac{\omega e^{i\omega t}}{\omega^2 - \omega_i^2} d\omega = i\pi e^{-\sqrt{-\omega_i^2}t}\,. \tag{S62}$$

The friction kernel in time domain is then retrieved as

$$\begin{aligned}\Gamma_R(t) = 2a\delta(t) + \frac{1}{2c_3}\sum_{i=1}^{3}\Bigg(&\frac{e^{-\sqrt{-\omega_i^2}t}}{\sqrt{-\omega_i^2}\prod_{j\neq i}(\omega_i^2 - \omega_j^2)}\left[k_0 + k_2\omega_i^2 + k_4(\omega_i^2)^2 + k_6(\omega_i^2)^3\right] \\ &- \frac{e^{-\sqrt{-\omega_i^2}t}}{\prod_{j\neq i}(\omega_i^2 - \omega_j^2)}\left[k_1 + k_3\omega_i^2\right]\Bigg).\end{aligned} \tag{S63}$$

This exemplary mapping shows, that a simple Markovian friction kernel given by eq. (S60) combined with a random force correlation function that contains additional oscillating components,given by eq. (S63), lead to the same correlation function as described by the GLE with the effective friction kernel given by eq. (19). We have thus derived one possible non-equilibrium model that describes CR cell motion.

Furthermore, this non-equilibrium model with $\Gamma_R(t)$ given by eq. (S63) and $\Gamma_v(t)$ given by eq. (S60) corresponds to the coupled system of differential equations

$$\dot{x}(t) = v(t) \tag{S64}$$

$$\dot{v}(t) = -a_v v(t) + \xi_x(t) + \sum_{i=1}^{3} F_i(t) \tag{S65}$$

$$\dot{F}_i(t) = -\frac{1}{\tau_i}\left(F_i(t) - \xi_i^R(t)\right) , \tag{S66}$$

with $\langle \xi_x(0)\xi_x(t)\rangle = 2aB\delta(t)$ and $\langle \xi_i^R(0)\xi_j^R(t)\rangle = 2a_i^R B\delta_{ij}\delta(t)$.
Solving eq. (S66) for $F_i(t)$ leads to

$$F_i(t) = -\frac{1}{\tau_i}\int_0^t e^{-(t-t')/\tau_i}\xi_i^R(t')dt' , \tag{S67}$$

which yields $\langle F_i(0)F_i(t)\rangle = B\frac{a_i^R}{\tau_i}e^{-t/\tau_i}$. Now defining the random force as $F_R(t) = \xi_x(t) + \sum_{i=1}^{3} F_i(t)$, we can write eq. (S65) as the GLE eq. (23) with

$$\Gamma_R(t) = 2a\delta(t) + \sum_{i=1}^{3}\frac{a_i^R}{\tau_i}e^{-t/\tau_i} \tag{S68}$$

and $\Gamma_v(t) = 2a_v\delta(t)$. Thus, the system of eqs. (S64)-(S66) is equivalent to the GLE eq. (23) with $\Gamma_R(t)$ given by eq. (S63) and $\Gamma_v(t)$ given by eq. (S60), when the parameters are given by

$$\tau_i = \frac{1}{\sqrt{-\omega_i^2}} \tag{S69}$$

$$a_i^R = \frac{1}{-2c_3\omega_i^2\prod_{j\neq i}(\omega_i^2-\omega_j^2)}\left[k_0 + k_2\omega_i^2 + k_4(\omega_i^2)^2 + k_6(\omega_i^2)^3 - \sqrt{-\omega_i^2}(k_1 + k_3\omega_i^2)\right] . \tag{S70}$$

XI Information on the cell orientation is contained in the cell-center trajectories

The mean direction of motion, exponentially weighted over the past trajectory, is defined by

$$\vec{V}(t) = \frac{1}{T}\int_{-\infty}^{t} e^{-(t-t')/T}\vec{v}(t')dt' \quad , \tag{S71}$$

where T is the exponential decay time and $\vec{v}(t) = (v_x(t), v_y(t))$ is the two-dimensional velocity (31). This running-average velocity vector $\vec{V}(t)$ points in the direction in which a cell has moved in the past, the running-average position follows as

$$\vec{R}(t) = \int_0^t \vec{V}(t')dt' . \tag{S72}$$

In Fig. S7a we show the trajectory of a synchro and its running average position $\vec{R}(t)$ for $T = 0.1\,\mathrm{s}$, $\vec{R}(t)$ resolves the mean direction of motion but not the back-and-forth motion on time scales below T. From the circular path of the trajectory it follows that the mean direction of motion changes, this is reflected in Fig. S7b by the change of the angle of $\vec{R}(t)$ with the x-axis (red data).

The orientation of cells is determined from the videos by fitting an ellipsoid to the cell perimeter and given by the main axis of the ellipsoid. Since CR cells move with their flagella in front, the orientation vector is defined to point towards the side where the flagella are anchored.

The cell orientation vector coincides very well with the direction of the running average velocity $\vec{V}(t)$, as shown in Fig. S7b. Therefore, the information on the orientation of the CR cells is included in the cell trajectory $(v_x(t), v_y(t))$ via eq. (S71). Our description of the CR motion by the GLE eq. (23) captures the correct behavior of the cell position and velocity over time, which is demonstrated by Figs. 4a-f. Since the cell orientational information is included in the trajectory, we conclude that the GLE model also correctly describes the CR orientational dynamics.

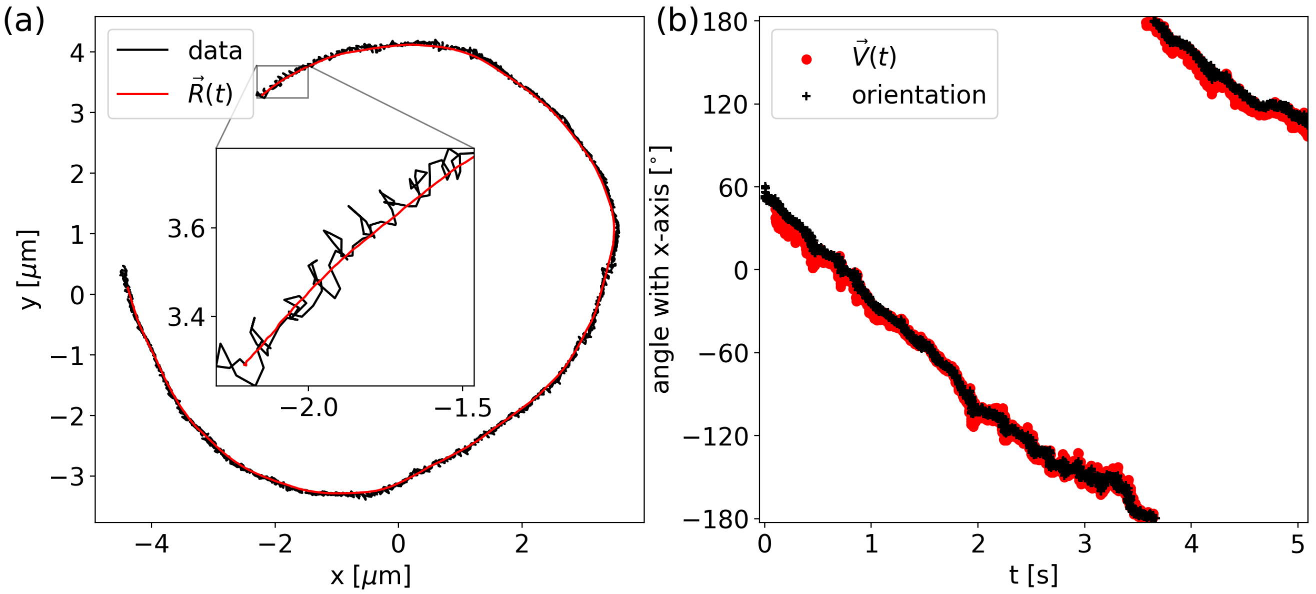

Figure S7: (a) Trajectory of a synchro shown in black, the running average position defined by eqs. (S71), (S72) with a decay time $T = 0.1\,\mathrm{s}$ is shown in red. The inset in the middle shows a zoom into the first $0.2\,\mathrm{s}$ of the trajectory. (b) The angle between the x-axis and the cell orientation directly extracted from video data is shown for the trajectory in (a) as black crosses. The angle between the x-axis and the running-average velocity $\vec{V}(t)$ determined by eq. (S71) is shown in red.

XII Effects of smoothing trajectory data

Since the localization noise modifies the VACF and thereby the friction kernel that is extracted from the data, it is important to smooth the data at a level that reduces the noise without loosing detail. For every data set, there is an optimal level of smoothing.

A natural way of smoothing data is by sequentially averaging over neighboring data points, which conserves the discretization time step. Here, we use an iterative averaging of the position data over two consecutive points according to

$$x_i^{n+1} = \frac{x_i^n + x_{i+1}^n}{2}, \qquad \text{(S73)}$$

with n being the number of smoothing iterations. We show the effect of the smoothing level on the VACF and the localization noise fit in Fig. S8 and the effect on the friction kernel in Fig. S9. For high smoothing iterations, the cell speed is underestimated, which is reflected by a decreasing mean squared velocity B for increasing n in Fig. S8. At the same time, the fitted localization noise strength σ_{loc} decreases with increasing n. This is reflected by the smaller difference between the first two data points of the VACF.

The estimate of the localization noise strength for high smoothing iterations n is not reliable anymore because it becomes very small, this explains why it is not decreasing monotonically with growing n, see Fig. S8. Since the first smoothing iteration $n = 1$ simply adds two consecutive points according to eq. (S73), one in principle expects the localization noise width σ_{loc} to be halved compared to the non-smoothed data. This indeed one can see for the synchro shown in Fig. S8.

Since the smoothing of the data decreases the amplitude of the VACF, as seen in Fig. S8, it consequently decreases the amplitudes of the friction kernel parameters a and b and therefore at the same time it reduces the kernel frequency Ω, as Ω exhibits a complex relation with the frequency of the VACF, which is explained in detail in Sec. V. Moreover, we find the dip in the friction kernel at the first time step to decrease for higher smoothing iterations shown in Fig. (S9), which indicates that the dip in the friction kernel originates from the localization noise. This is explained by eq. (8), since the first point of the VACF is overestimated and the second point of the VACF is underestimated due to the localization noise, which then propagates into the friction kernel and leads to a dip at short times.

The VACF of the smoothed data is less influenced by the localization noise, nevertheless, with every smoothing iteration, the deviation of the smoothed VACF from the non-smoothed VACF becomes larger, as shown in Fig. S8. Thus, we

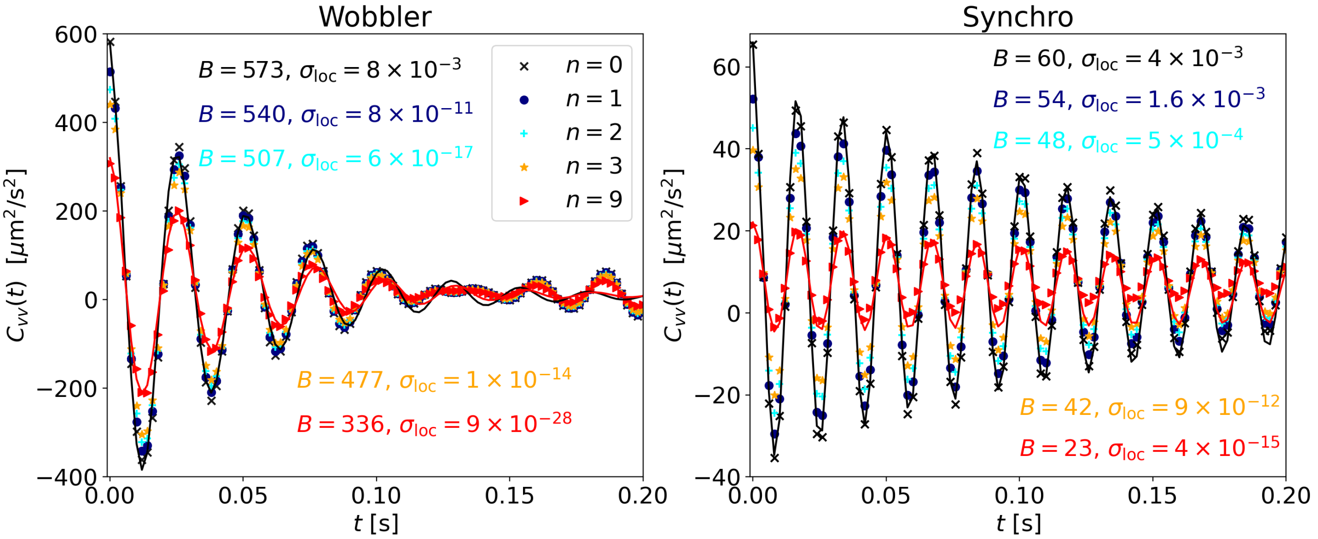

Figure S8: The VACF $C_{vv}(t)$ of a wobbler (left) and a synchro (right) is shown for different smoothing iterations n described by eq. (S73), where $n = 0$ denotes the original non-smoothed data. The mean-squared velocities B in units of $\mu\mathrm{m}^2/\mathrm{s}^2$ and the fitted localization noise width $\sigma_{\rm loc}$ in units of $\mu\mathrm{m}$ are both given in the color of the according smoothing iteration. The fitting result of the VACF including localization noise, described in Sec. VII, is shown as solid lines for $n = 0$ and $n = 9$ in the respective color.

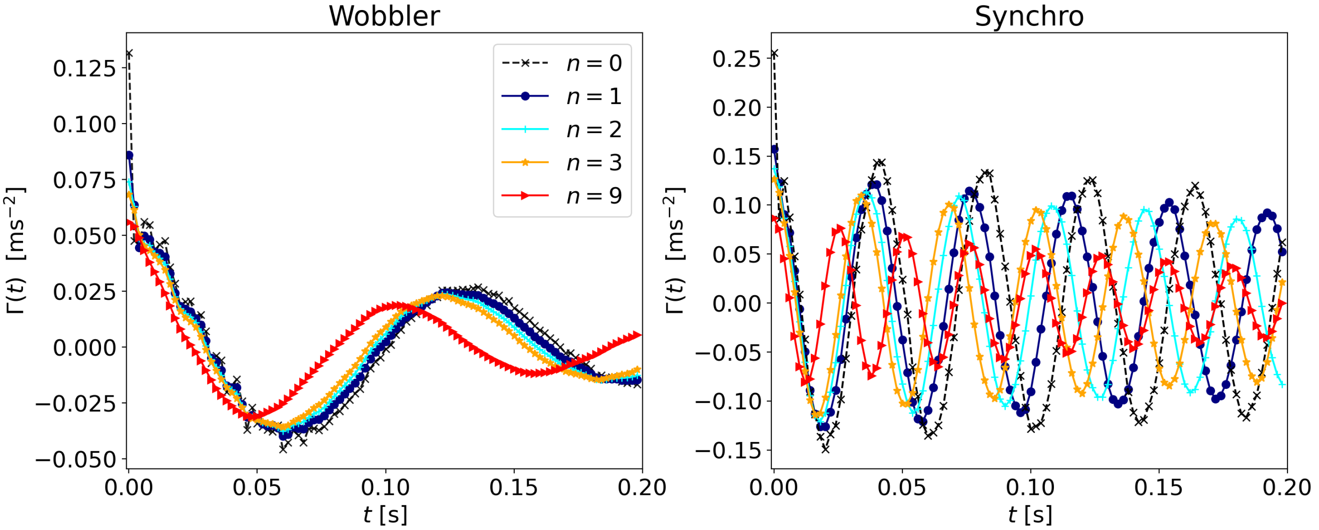

Figure S9: The friction kernel $\Gamma(t)$ extracted according to eq. (8) from data at different smoothing iterations described by eq. (S73) is given for a wobbler on the left and for a synchro on the right. The solid lines connecting the data points are guides to the eye.

choose $n = 1$ smoothing iterations for our data as a compromise between minimizing the effect of noise and keeping a good resolution that accurately describes the actual cell velocities.

XIII Markovian embedding of the friction kernel eq. (19)

Here we derive the equivalency of the system of coupled equations of motion

$$
\begin{aligned}
\dot{x}(t) &= v(t) && \text{(S74)}\\
m\dot{v}(t) &= -\gamma_x v + bm(y(t) - x(t)) + F_{Rx}(t) && \text{(S75)}\\
\dot{y}(t) &= v_y(t) && \text{(S76)}\\
m_y\dot{v}_y(t) &= -\gamma_y v_y + bm(x(t) - y(t)) + F_{Ry}(t)\,, && \text{(S77)}
\end{aligned}
$$

describing two harmonically coupled massive reaction coordinates in a heat bath based on the Hamiltonian eq. (20), to the GLE eq. (23) with the effective friction kernel of eq. (19) (61). Here, the random forces have zero mean $\langle F_i(t)\rangle = 0$ and are delta correlated $\langle F_i(0)F_j(t)\rangle = 2\gamma_i k_B T\delta_{ij}\delta(t)$. We start by finding the solution for $\vec{z}(t) = (y, \dot{y})^T$ to the subsystem eqs. (S76) and (S77), which is the solution to

$$
\dot{\vec{z}}(t) = A\vec{z}(t) + \begin{pmatrix} 0 \\ F_{Ry}(t)/m_y + \frac{bm}{m_y}x(t) \end{pmatrix} \tag{S78}
$$

with

$$
A = \begin{pmatrix} 0 & 1 \\ -\frac{bm}{m_y} & -\frac{\gamma_y}{m_y} \end{pmatrix} \tag{S79}
$$

and consequently

$$
A^{-1} = \begin{pmatrix} -\frac{\gamma_y}{bm} & -\frac{m_y}{bm} \\ 1 & 0 \end{pmatrix}\,. \tag{S80}
$$

One finds the solution to eq. (S78) as

$$
\vec{z}(t) = e^{A[t-t_0]}\vec{z}(t_0) + \int_{t_0}^{t} dt' e^{A[t-t']} \begin{pmatrix} 0 \\ F_{Ry}(t')/m_y + \frac{bm}{m_y}x(t') \end{pmatrix}\,. \tag{S81}
$$

Performing a partial integration on the integral in eq. (S81) for the term including $x(t')$ leads to

$$
\begin{aligned}
\vec{z}(t) = e^{A[t-t_0]}\vec{z}(t_0) &+ \int_{t_0}^{t} dt' A^{-1} e^{A[t-t']} \begin{pmatrix} 0 \\ \frac{bm}{m_y}v(t') \end{pmatrix} \\
&- \left[A^{-1}e^{A[t-t']}\begin{pmatrix} 0 \\ \frac{bm}{m_y}x(t') \end{pmatrix}\right]_{t_0}^{t}\,, \\
&+ \int_{t_0}^{t} dt' e^{A[t-t']} \begin{pmatrix} 0 \\ F_{Ry}(t')/m_y \end{pmatrix}\,,
\end{aligned} \tag{S82}
$$

where the matrix exponential e^{At} can be expressed in terms of the Eigenvalues of A

$$
\lambda_{1,2} = -\frac{\gamma_y}{2m_y} \pm \omega_0 \tag{S83}
$$

with

$$
\omega_0 = \sqrt{\left(\frac{\gamma_y}{2m_y}\right)^2 - \frac{bm}{m_y}} \tag{S84}
$$

as

$$
\begin{aligned}
e^{At} &= \frac{1}{\lambda_2 - \lambda_1}\begin{pmatrix} \lambda_2 e^{\lambda_1 t} - \lambda_1 e^{\lambda_2 t} & e^{\lambda_2 t} - e^{\lambda_1 t} \\ \lambda_1\lambda_2(e^{\lambda_1 t} - e^{\lambda_2 t}) & \lambda_2 e^{\lambda_2 t} - \lambda_1 e^{\lambda_1 t} \end{pmatrix} \\
&= e^{-t\frac{\gamma_y}{2m_y}}\begin{pmatrix} \cosh(\omega_0 t) + \frac{\sinh(\omega_0 t)\gamma_y}{2m_y\omega_0} & \sinh(\omega_0 t)/\omega_0 \\ -\frac{\sinh(\omega_0 t)bm}{\omega_0 m_y} & \cosh(\omega_0 t) - \frac{\sinh(\omega_0 t)\gamma_y}{2m_y\omega_0} \end{pmatrix}
\end{aligned} \tag{S85}
$$

consequently leading to

$$
A^{-1}e^{At} = e^{-t\frac{\gamma_y}{2m_y}}\begin{pmatrix} -\frac{\cosh(\omega_0 t)\gamma_y}{bm} + \frac{\sinh(\omega_0 t)}{\omega_0}(1 - \frac{\gamma_y^2}{2bmm_y}) & -\frac{\sinh(\omega_0 t)\gamma_y}{2bm\omega_0} - \frac{\cosh(\omega_0 t)m_y}{bm} \\ \cosh(\omega_0 t) + \frac{\sinh(\omega_0 t)\gamma_y}{2m_y\omega_0} & \frac{\sinh(\omega_0 t)}{\omega_0} \end{pmatrix}\,. \tag{S86}
$$

16

The solution for $y(t)$ can now be found as the first element of the vector $\vec{z}(t)$ from eqs. (S82)-(S86) when assuming a stationary system, i.e. $t_0 \to -\infty$ as

$$\begin{aligned} y(t) = x(t) - \int_{-\infty}^{t} dt' v(t') e^{-\frac{(t-t')\gamma_y}{2m_y}} \left(\cosh(\omega_0(t-t')) + \frac{\gamma_y}{2m_y\omega_0} \sinh(\omega_0(t-t')) \right) \\ + \int_{-\infty}^{t} dt' F_{Ry}(t') e^{-\frac{(t-t')\gamma_y}{2m_y}} \frac{\sinh(\omega_0(t-t'))}{m_y\omega_0}. \end{aligned} \tag{S87}$$

Inserting eq. (S87) into eq. (S75), one recovers the GLE eq. (23) for $x(t)$

$$m\ddot{x}(t) = -\int_{-\infty}^{t} dt' \bar{\Gamma}(t-t') + \bar{F}_R(t), \tag{S88}$$

where the bars indicate multiplication by the mass $\Gamma(t) = \bar{\Gamma}(t)/m$, $F_R = \bar{F}_R/m$ and the friction kernel takes the oscillatory form

$$\Gamma(t) = \frac{\gamma_x}{m}\delta(t) + be^{-t\frac{\gamma_y}{2m_y}} \left(\cosh(\omega_0 t) + \frac{\gamma_y}{2m_y\omega_0} \sinh(\omega_0 t) \right) \tag{S89}$$

and the random force is explicitly given by

$$F_R(t) = \bar{F}_{Rx}/m + b \int_{-\infty}^{t} dt' \bar{F}_{Ry}(t') e^{-\frac{(t-t')\gamma_y}{2m_y}} \frac{\sinh(\omega_0(t-t'))}{m_y\omega_0}\,. \tag{S90}$$

Computing the random force correlation, one finds

$$\langle F_R(0) F_R(t) \rangle = B\Gamma(t)\,. \tag{S91}$$

The friction kernel of eq. (19) in the Methods is equivalent to the friction kernel of eq. (S89) with the parameters given by

$$\begin{aligned} a &= \frac{\gamma_x}{m} \\ \tau &= \frac{2m_y}{\gamma_y} \\ \Omega &= i\omega_0\,. \end{aligned}$$
